# Serendipity-Driven Telescoped Synthesis of 2‑Aryl Glycidic Esters from Aldehydes

**DOI:** 10.1021/acs.orglett.5c05362

**Published:** 2026-01-19

**Authors:** Vincenzo Battaglia, Isaac G. Sonsona, Sara Meninno, Carlo Crescenzi, Alessandra Lattanzi

**Affiliations:** † Dipartimento di Chimica e Biologia “A. Zambelli”, 19028 Università di Salerno, Via Giovanni Paolo II 132, 84084 Fisciano, Italy; ‡ Dipartimento di Farmacia, Università di Salerno, Via Giovanni Paolo II 132, 84084 Fisciano, Italy

## Abstract

A first general and practical method for the synthesis of valuable 2-(hetero)­aryl glycidic ethyl esters has been developed using commercially available reagents and a catalyst. A telescoped three-step seven-reaction process, based on a Knoevenagel/nitro-Michael/hydroxylation/double elimination/epoxidation/esterification sequence, provides the epoxides in up to 73% overall yield. This one-pot protocol features (i) aldehydes as versatile feedstocks, among the reagents used in the process; (ii) all steps proceeding at room temperature in benign solvents; and (iii) scalability of the reaction up to 5 mmol. The epoxides are elaborated to obtain new attractive β-amino α-hydroxy esters, bearing a quaternary stereocenter, including tryptamine- and morpholine-based esters.

## Graphical abstract

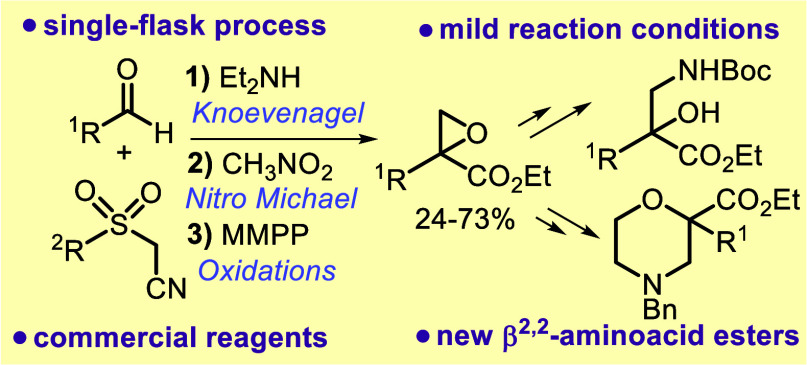

Glycidic esters belong to a class of epoxides with important applications in the synthesis of drugs^1^ and are industrially relevant in epoxide resin production.^2^ Their ring-opening reactions have been fruitfully explored for the synthesis of some representative pharmaceuticals such as anticancer adjuvant bestatin,^3^ antihypertensive agent diltiazem,^4^ or antitumor drug paclitaxel.^5^ A literature survey showed a focus on the synthesis of 3-aryl-substituted glycidic esters as targets,^6^ whereas methods for obtaining 2-aryl glycidic esters have not been developed. A typical oxidative approach for their synthesis involves a stepwise process with a first preparation of the α-aryl acrylates, starting from arylacetic acids or esters (Scheme 1a,b). The olefination step is then carried out with formaldehyde in DMF under reflux^7^ (Scheme 1a). Alternatively, α-oxidation is necessary to obtain the α-keto ester, before performing a Wittig reaction under controlled conditions at −78 °C (Scheme 1b).^8^ A single-pot synthesis of atropates has been recently reported starting from terminal alkynes in a palladium/ligand-catalyzed hydroesterification using carbon monoxide under high pressure at room temperature (Scheme 1c).^9^ The epoxidation of the alkene is the final step generally carried out with peroxyacids at temperatures higher than ambient temperature.^10^ Being interested in the synthesis of amino acid derivatives,^11^ we designed a rapid approach for obtaining the β-nitro ester precursors of β-amino acids, through a one-pot Knoevenagel/nitro-Michael reaction followed by the α-hydroxylation of the adduct with magnesium monoperoxyphthalate (MMPP) in EtOH^12^ (Scheme 2). Intermediate **I** would have undergone elimination of HCN to form a α-ketosulfone and then been attacked *in situ* by the alcohol to give the β-nitro ester.^13^ In preliminary experiments, the formation of the β-nitro ester was accompanied by the presence of the 2-aryl glycidic ester as a side product. This serendipitous result and the lack of a straightforward access to versatile 2-aryl glycidic esters were instrumental in the development of a method for their synthesis.

**Scheme 1:**
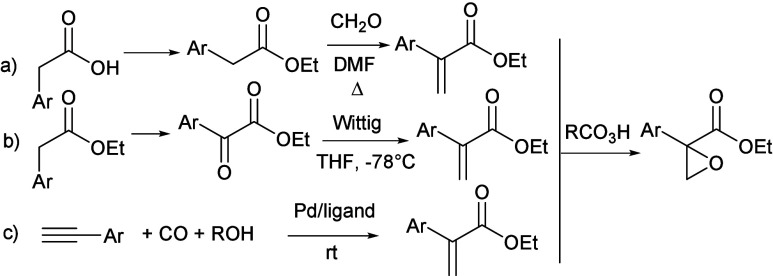
Common Stepwise Approaches to 2-Aryl Glycidic Esters

**Scheme 2:**
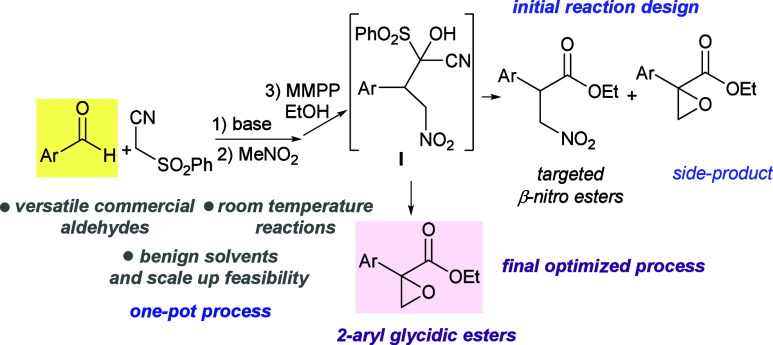
Synthetic Sequence Leading to Unanticipated 2-Aryl Glycidic Esters

Herein, we illustrate a mild one-pot protocol, developed using commercial reagents and a catalyst, including aldehydes as key feedstocks and benign solvents, which enables us to obtain 2-aryl glycidic esters in good to high overall yields.

The approach can be scaled up to 5 mmol, and the epoxides elaborated to be transformed into new useful β-amino-α-hydroxy ester derivatives.

At the outset, the diethyl amine-catalyzed Knoevenagel reaction between aromatic aldehydes and phenylsulfonyl acetonitrile in EtOH at room temperature was chosen as the most effective and convenient protocol to obtain the alkenes, given the usage of a readily available base and ethanol as the solvent.^14^ Pleasingly, the nitro Michael reaction with model alkene **1a**, performed under the same conditions, proceeded rapidly to give adduct **2a** in 91% yield (Scheme 3).

**Scheme 3:**
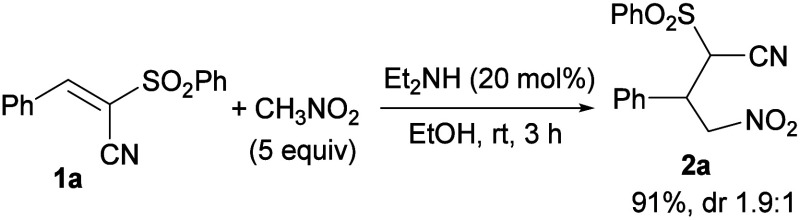
Nitro Michael Reaction of Alkene **1a** Catalyzed by Diethyl Amine in Ethanol

The reaction outcome met our expectations for being used as the second step of the one-pot sequence. Having secured the effective formation of adduct **2a**, the α-hydroxylation step, initially designed for the synthesis of β-nitro methyl ester (Scheme 2), was performed under the previously reported conditions.^12^ The α-hydroxy intermediate, according to the literature,^13^ would have led to the β-nitro ester (Table 1). Under typical conditions,^15^ adduct **2a** reacted in MeOH, affording expected ester **4a′** (entry 1). Moreover, 2-phenyl glycidic methyl ester **3a′** was surprisingly detected, together with traces of α-phenyl acrylate **5a′** (entry 1). As anticipated, we became motivated to optimize the reaction conditions with the thought of developing a general one-pot method to access 2-aryl glycidic esters. Increasing the amount of MMPP with K_2_CO_3_ as the base provided a better conversion to nitro ester **4a′** (entry 2). Interestingly, epoxide **3a** was obtained as the major product when EtOH was employed (entry 3). The use of an excess of MMPP slightly improved the selectivity of the reaction mixture (entry 4). Remarkably, the reaction performed in a THF/EtOH solvent mixture^16^ enabled to reach higher and selective conversion to epoxide **3a**, as only traces of products **4a** and **5a** were detected (entry 5). Common bases were then evaluated under these conditions (entries 6–9). Being that the reaction mixtures were not homogeneous, a clear trend was not observed between base strength and the yield obtained. NaOH and K_2_CO_3_ proved to be the most effective, with the former providing the highest conversion and selectivity (entry 9). Finally, THF was successfully replaced by a greener alternative, 2-methyl tetrahydrofuran (2-MeTHF) (entry 10). Further optimization did not improve the reaction outcome (Table S1).

**Table 1:**
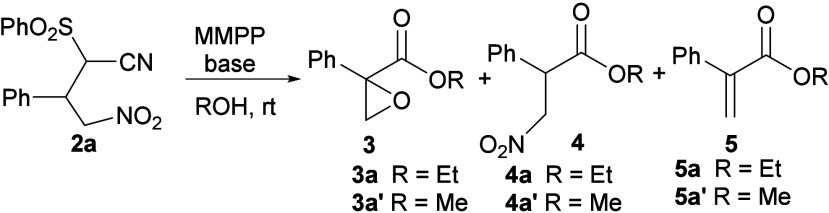
Optimization of the One-Pot Oxidation of **2a** with MMPP^a^

entry	base (equiv)	equiv of MMPP	ROH	*t* (h)	yield of **3**/**4**/**5** (%)^b^
1^c^	Li_2_CO_3_ (0.7)	1	MeOH	20	14/44/3
2	K_2_CO_3_ (1.5)	1.5	MeOH	3	15/54/3
3	Li_2_CO_3_ (1.5)	1	EtOH	2	43/25/3
4	Li_2_CO_3_ (1.5)	2	EtOH	2	48/20/-
5^d^	Li_2_CO_3_ (1.5)	2	EtOH	24	70/4/2
6^d^	K_2_CO_3_ (1.5)	2	EtOH	24	73/4/2
7^d^	Cs_2_CO_3_ (1.5)	2	EtOH	24	58/2/1
8^d^	LiOH (1.5)	2	EtOH	24	71/5/3
9^d^	NaOH (1.5)	2	EtOH	24	74/2/2
10^e^	NaOH (1.5)	2	EtOH	24	73/3/2

a Reaction conditions: compound **2a** (0.1 mmol), base (0.07–0.15 mmol, 0.7–1.5 equiv), and MMPP (0.1–0.2 mmol, 1–2 equiv) in anhydrous MeOH or EtOH (1 mL).

b Yield determined by ^1^H NMR analysis of the crude reaction mixture using tetrachloroethane as an internal standard.

c With 0.7 mL of anhydrous MeOH.

d A 4:1 THF/EtOH mixture (2.5 mL) was used as the solvent.

e A 4:1 2-MeTHF/EtOH mixture (2.5 mL) was used as the solvent.

Under the optimized conditions, the one-pot synthesis of racemic 2-aryl glycidic ethyl esters **3** was next studied (Scheme 4). The epoxides were generally obtained in good to high overall yields. Model 2-phenyl glycidic ethyl ester **3a** was isolated in 61% yield, attesting to the fact that the diethyl amine-catalyzed Knoevenagel/nitro-Michael reactions proceeded in high yields, upon comparison of this result with the conversion observed for epoxide **3a** in the MMPP oxidation sequence (73% yield, entry 10 of Table 1). *para*-Electron-donating groups or halogen atoms at different positions of the phenyl ring were well tolerated as epoxides **3b**–**h** were isolated in 37–55% yields. Epoxides **3i**–**l**, phenyl substituted with *para*- and *ortho*-electron-withdrawing groups, were recovered in very good and up to 73% yields. Inferior conversions are observed for epoxides derived from more sterically demanding *ortho*-substituted aldehydes. Epoxides **3m**–**o**, bearing double substitution or unsaturated groups on the phenyl ring, were obtained in satisfactory to fairly good yields (up to 68%). Finally, 2-naphthyl-substituted and heteroaromatic 3-furyl- and 3-indolyl-substituted glycidic ethyl esters **3p**–**r** were isolated in moderate to good yields (up to 45%). Remarkably, although the furyl ring is known to suffer oxidation by peracids,^17^ epoxide **3q** was successfully obtained in 24% overall yield, the MMPP oxidation being performed at −20 °C. However, oxidant susceptible pyridine- and thiophene-based aldehydes turned out to be unsuccessful reagents. The process proceeded slightly less efficiently when applied to prepare model epoxide **3a** at a 5 mmol scale, whereas improvements were observed for epoxide **3r**, obtained in 47% yield, working at a 1 mmol scale and using 40 mol % Et_2_NH. Application of the protocol to prepare 2-aliphatic glycidic esters proved to be unsuccessful. When starting from the isobutyl-substituted alkene, a complex mixture was obtained and traces of the corresponding ethyl β-nitro ester were observed.

**Scheme 4:**
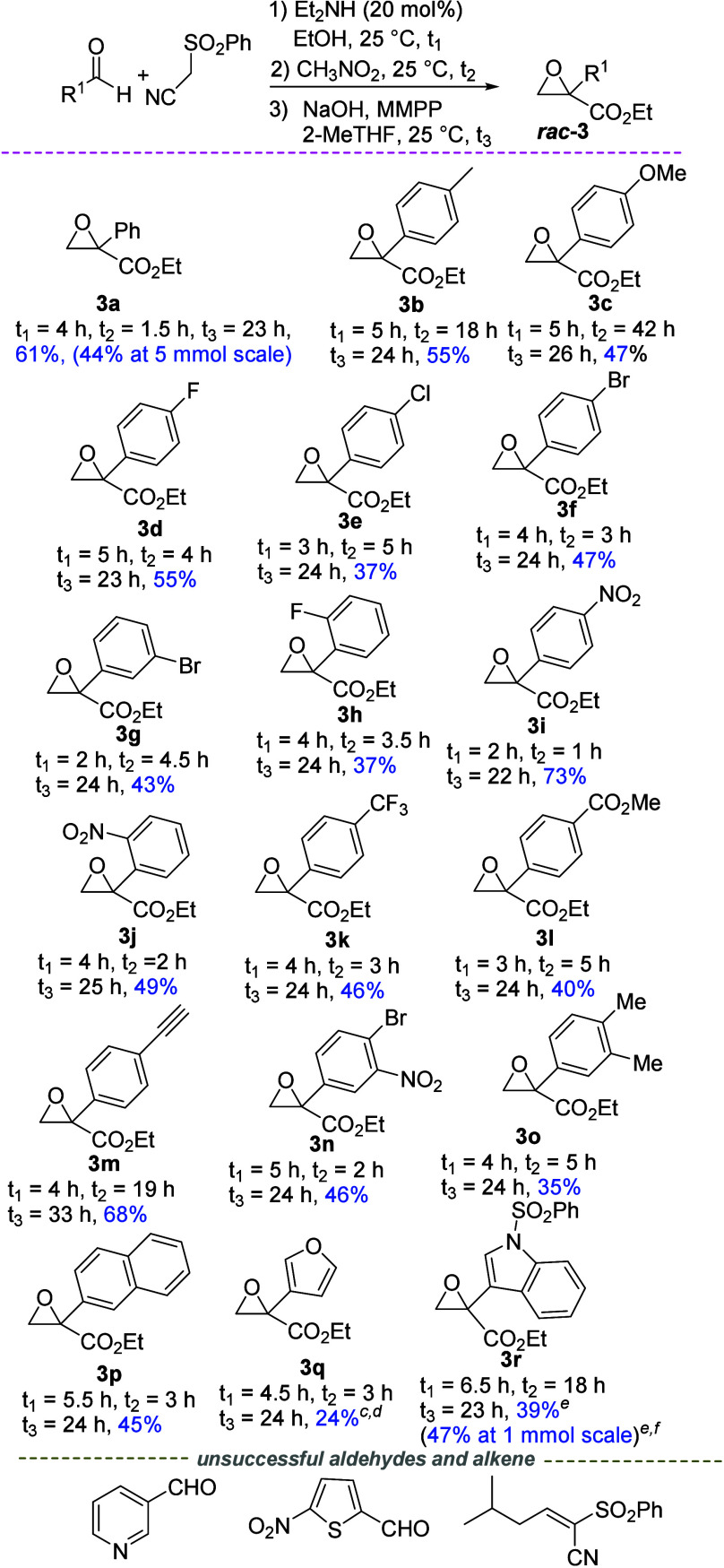
One-Pot Telescoped Synthesis of 2-Aryl Glycidic Esters ***rac*** **-3** ^a^,^b^

a Reaction conditions: (1) Knoevenagel step with (phenylsulfonyl)­acetonitrile (0.20 mmol), aldehyde (0.22 mmol), and Et_2_NH (0.04 mmol) in anhydrous EtOH (1 mL); (2) nitro Michael addition step with addition of CH_3_NO_2_ (1.0 mmol); (3) dilution of the reaction mixture with 2-MeTHF (4 mL) and addition of MMPP (0.45 mmol) and NaOH (0.30 mmol).
b Yield of the isolated product after chromatography.
c Step 3 was carried out at −20 °C.
d Reaction carried out at a 0.5 mmol scale due to the volatility of product **3q**.
e The reaction mixture was diluted with 2-MeTHF (4 mL at 0.2 mmol scale, 40 mL at 1.0 mmol scale) before the addition of nitromethane in step 2.
f With 40% Et_2_NH and 10 mL of anhydrous EtOH (*t*_1_ = 23 h; *t*_2_ = 22 h; *t*_3_ = 24 h).

For the sake of comparison, alkylidene derived from benzaldehyde and malononitrile was treated under the conditions reported in Scheme 4 (for steps 2 and 3). Compound **4a** was recovered in 25% yield, whereas epoxide **3a** was not detected. Notably, the presence of the phenylsulfone group on the alkene appears to be crucial for the reaction outcome.^15,18^

Surprisingly, ring-opening reactions of 2-aryl glycidic ethyl esters have rarely been explored for derivatization, in contrast to their 3-aryl glycidic ester counterparts. Given a facile access to terminal glycidic esters **3**, a variety of postfunctionalizations with different nitrogen-based nucleophiles were designed to prepare non-natural β^2,2^-amino acid derivatives, taking advantage of the highly regioselective ring-opening reactions they undergo (Scheme 5). The members of this class of amino acids, including α-hydroxy-β-amino acid derivatives, are relevant scaffolds in the pharmaceutical industry, subunits useful for the synthesis of natural products and drugs.^19^

**Scheme 5:**
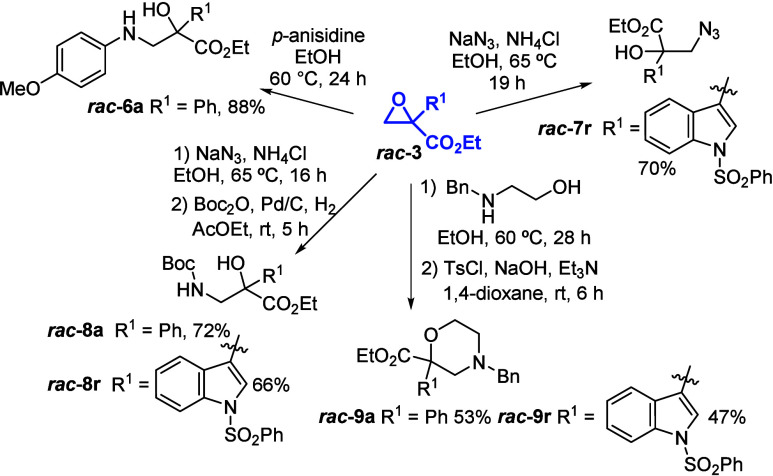
Synthetic Elaborations of 2-Aryl Glycidic Esters

Epoxide **3a** treated with *p*-anisidine afforded α-hydroxy-β-amino ester **6a** in 88% yield. NaN_3_ was then employed to prepare functionalized precursors to use in click chemistry as a tool to facilitate drug discovery.^20^ Specifically, starting from indolyl-substituted epoxide **3r**, alcohol **7r** was obtained in 70% yield. Further reduction, with *in situ* Boc protection of the primary amine, afforded simple access to new tryptamine derivative **8r**, bearing a quaternary stereocenter, in 66% overall yield. Tryptamine derivatives are often used as intermediates for the synthesis of drugs and are key units of alkaloids.^21^ The same process, applied to model epoxide **3a**, led to *N*-Boc α-hydroxy-β-amino ethyl ester **8a** in 72% overall yield. Finally, an approach to construct new morpholine esters has been showcased through *N*-benzyl ethanol amine epoxide ring opening followed by tosylation and intramolecular nucleophilic substitution. Accordingly, morpholines **9a** and **9r** were obtained in 53% and 47% yields, respectively.^22^ This route highlights 2-aryl glycidic esters as versatile building blocks to access unprecedented morpholine esters **9**, which could be used to obtain a variety of morpholinol derivatives, key compounds involved in the synthesis of neurokinin receptor antagonists.^23^ To determine the entire reaction pathway, a control experiment was first performed. In the preliminary study illustrated in Table 1, traces of acrylate **5a** were detected. Hence, to assess if the epoxidation occurred on acrylate **5a**, this compound was treated under the same oxidative conditions reported in Scheme 4, either at room temperature or at 50 °C (Scheme 6). The process was also carried out in the absence of a base (Table S2). Acrylate **5a** proved to be unreactive, a result in agreement with the literature, indicating that harsher reaction conditions are necessary for the epoxidation to proceed.^10^ This result suggested that another alkene intermediate would undergo epoxidation by MMPP. Oxidation on compound **2r**, carried out under the same conditions, was next studied over time via HRMS analysis.^24^ Besides epoxide **3r**, intermediate **II′** was detected, which is a postulated precursor of observed nitro ester **4** (Table 1).

**Scheme 6:**
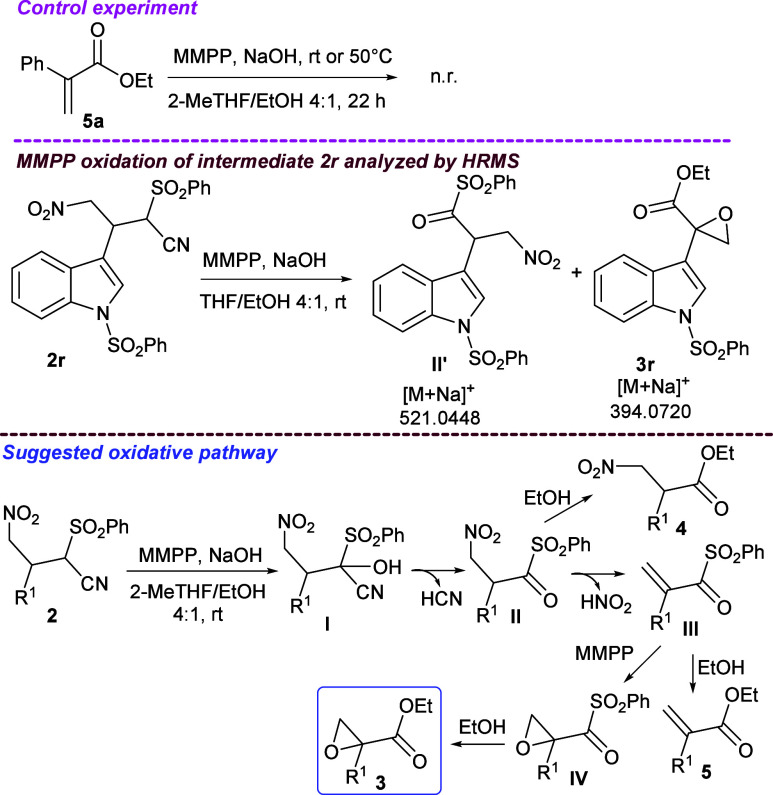
Oxidation of Compounds **5a** and **2r** with MMPP and a Suggested Pathway

On the basis of all of the data, a plausible pathway is proposed to occur in the MMPP oxidation of nitro-Michael adducts **2** to epoxides **3** (Scheme 6). Compound **2** is hydroxylated to intermediate **I**, which undergoes the elimination of HCN to give crucial intermediate **II**. According to their structure, nitro compounds are prone to eliminate nitrous acid under basic conditions.^25^ In a THF or 2-MeTHF/EtOH medium, intermediate **II** would eliminate nitrous acid to afford alkene **III**, which being more electrophilic than acrylate **5**, is susceptible to nucleophilic epoxidation by MMPP,^26^ giving intermediate **IV**. Indeed, we previously observed that intermediates of type **II** are not easily attacked by EtOH^11a^ and sterically hindered alcohols to give the ester when compared with MeOH. This would make intermediate **II** more susceptible to elimination to afford alkene **III** than esterification to afford compound **4**. This reactivity is expected to be enhanced in the 2-MeTHF/EtOH mixture, where higher conversion and selectivity toward the epoxide pathway were observed (Table 1). However, in pure alcoholic media, intermediate **II** would evolve into β-nitro ester **4** to a significant extent before the elimination of nitrous acid takes place. Epoxy intermediate **IV**, once formed, undergoes esterification by ethanol to give epoxide **3**. Partial esterification of intermediate **III** would account for the presence of acrylate **5**, detected in the MMPP oxidation of **2a** (Table 1).

In conclusion, we developed a first general and convenient route to easily prepare 2-aryl glycidic ethyl esters from commercial sources, including aldehydes as feedstocks. Remarkably, the one-pot sequential process can be carried out at room temperature in benign solvents, enabling an effective synthesis of the epoxides in good to high overall yields. The protocol can be scaled up, and the synthetic utility of the 2-aryl glycidic esters has been demonstrated. By leveraging highly selective ring-opening reactions, interesting new α-hydroxy-β-amino acid derivatives, encompassing tryptamine and morpholine esters, can be obtained.

## Supplementary Material



## Data Availability

The data underlying this study are available in the published article and its Supporting Information.

## Notes

Full experimental procedures, characterization data, and NMR spectra (PDF)
