# Supplementary material for: Serendipity-Driven Telescoped Synthesis of 2‑Aryl Glycidic Esters from Aldehydes
Source: Org Lett. 2026 Jan 19;28(4):1457–62. doi: 10.1021/acs.orglett.5c05362 (PMC12865760; doi:10.1021/acs.orglett.5c05362)
Supplement: Supplementary file 1 [file ol5c05362_si_001.pdf]

Serendipity-Driven Telescoped Synthesis of 2-Aryl Glycidic Esters from Aldehydes

Vincenzo Battaglia,[§ǂ] Isaac G. Sonsona,[§ǂ] Sara Meninno,[§] Carlo Crescenzi,[‡] and Alessandra Lattanzi*[,§]

§Dipartimento di Chimica e Biologia "A. Zambelli", Università di Salerno, Via Giovanni Paolo II 132, 84084, Fisciano, Italy

‡Dipartimento di Farmacia, Università di Salerno, Via Giovanni Paolo II 132, 84084, Fisciano, Italy

*E-mail: lattanzi@unisa.it

General Methods 2

Optimization of the reaction conditions 3

Table S1. Optimization of the oxidation of **2a** in the presence of MMPP (additional experiments).......... 3

Experimental Procedures and Compounds Characterization 4

General procedure for the one-pot synthesis of the epoxides **3a-r**. 4

Synthesis of the intermediate **2a**.......... 10

One-pot synthesis of the intermediate **2r**.......... 11

Opening of the epoxide **3a** with *p*-anisidine 12

Synthesis of the indole-derived azide **7r** 12

Multi-step synthesis of the *N*-Boc protected *β*-amino ester **8a** 13

Multi-step synthesis of the indole-derived *N*-Boc protected *β*-amino ester **8r** 14

Opening of the epoxide **3a** with 2-(benzylamino)ethan-1-ol 15

Synthesis of the morpholine derivative **9a** 16

Multi-step synthesis of the indole-derived morpholine derivative **9r** 17

Scale-Up of the Reaction 18

One-pot synthesis of the epoxide **3a** from benzaldehyde (5 mmol scale) 18

One-pot synthesis of the epoxide **3a** from the corresponding alkene (6 mmol scale) 18

One-pot synthesis of the epoxide **3r** (1 mmol scale) 19

Mechanistic Studies 20

Table S2. Epoxidation of ethyl 2-phenylacrylate **5a** in presence of MMPP.......... 20

Analysis of the reaction mixture by HRMS.......... 20

Table S3. ESI-HRMS analysis of the reaction mixture (**2r** in presence of NaOH and MMPP).......... 21

NMR Spectra.......... 24

General Methods

Anhydrous 1,4-dioxane was purchased from Merck-Sigma Aldrich and used as received. All other solvents were dried over molecular sieves (Merck-Sigma Aldrich Molecular Sieves, 3 Å, 1.6 mm pellets). For activation, the molecular sieves were heated overnight at 200 ºC under vacuum. Reactions were monitored by thin-layer chromatography (TLC) on Macherey-Nagel precoated silica gel plates (0.25 mm) and visualized under UV light with phosphomolybdic acid as the TLC stain. Column chromatography was performed using Merck silica gel (60, particle size: 0.040–0.063 mm) as the stationary phase. ^{1}H NMR, proton-decoupled ^{13}C {^{1}H} NMR and proton-decoupled ^{19}F {^{1}H} NMR spectra were recorded on Bruker Avance III HD 600, Bruker Avance III HD 400, Bruker Avance III HD 300 or Bruker Avance III HD 250 spectrometers in deuterated chloroform ($CDCl_3$) as solvent. Chemical shifts for protons are reported using residual solvent protons (δ = 7.26 ppm for $CDCl_3$) as an internal standard. Carbon spectra were referenced to the shift of the ^{13}C signal of $CDCl_3$ (δ = 77.0 ppm). The following abbreviations are used to indicate the multiplicity in NMR spectra: s – singlet; d – doublet; t – triplet; q – quartet; dd – doublet of doublets; ddd – doublet of doublet of doublets; dt – doublet of triplets; dq – doublet of quartets; td – triplet of doublets; m – multiplet; bs – broad singlet; pd – pseudo-doublet. MALDI-HRMS spectra were acquired in positive ionization mode using a Bruker solariX XR Fourier transform ion cyclotron resonance mass spectrometer (Bruker Daltonik GmbH, Bremen, Germany) equipped with a 7 T refrigerated actively-shielded superconducting magnet. ESI-HRMS spectra were acquired using Thermo Scientific LTQ-Orbitrap XL mass spectrometer (Thermo Fisher Scientific, Dreieich, Germany) operated in positive ionization mode in the range m/z 100-1000 and with mass resolution set at 100000. For the detection of reaction intermediates by ESI-HRMS, the data recorded were processed with Xcalibur 2.0 software (Thermo Fisher Scientific, Dreieich, Germany). Melting points were measured with a Stuart Model SMP 30 melting point apparatus and are uncorrected. Petroleum ether (PE) refers to light petroleum ether (boiling point 40-60 °C). All starting materials (unless otherwise noted) were purchased from Merck-Sigma Aldrich, TCI-Europe or Fluorochem and used as received. (*E*)-3-phenyl-2-(phenylsulfonyl)acrylonitrile[1] and ethyl 2-phenylacrylate (**5a**)[2] were synthesized following the protocols reported in the literature and the spectroscopic data obtained for these compounds are in agreement with those previously reported. Spectroscopic data obtained for the product **3a'**[3] and the side products **4a**,[4] **4a'**,[4] and **5a'**,[5] are in agreement with those previously reported in the literature.

Optimization of the reaction conditions

Table S1. Optimization of the oxidation of **2a** in the presence of MMPP (additional experiments).[a]

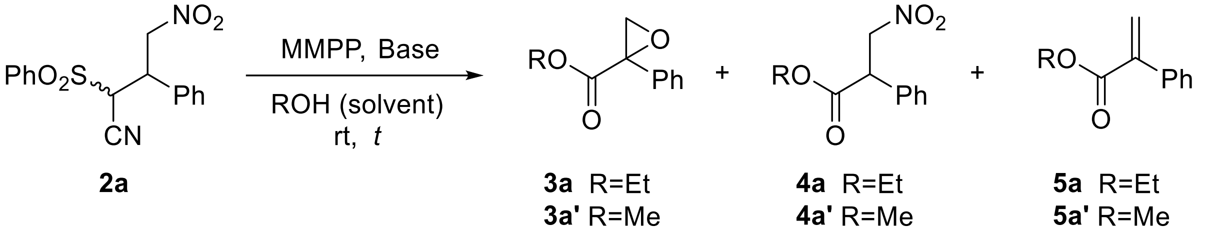

Entry	Solvent	C (M)	MMPP (equiv)	Base or additive	R	t (h)	Yield (%)[b] **3** / **4** / **5**
1	MeOH	0.1	2	-	Me	3	21 : 59 : -
2	MeOH	0.05	1.5	-	Me	3	14 : 68 : 5
3	MeOH	0.05	1	$NaHCO_3$ (1.0 equiv)	Me	3	14 : 66 : 6
4	MeOH	0.05	0.5	-	Me	3	12 : 51 : 6
5	MeOH	0.05	0.5	BzOH (0.5 equiv)	Me	3	4 : 25 : 3
6	EtOH	0.1	2	Li_2CO_3 (1.5 equiv)	Et	24	34 : 24 : -
7	EtOH	0.1	2	Li_2CO_3 (3.0 equiv)	Et	3	46 : 18 : -
8	THF/EtOH 2:1	0.03	2	Li_2CO_3 (1.5 equiv)	Et	24	66[c] : 5[c] : -
9	$(CH_3)_2CHCH_2CH_2OH$	0.1	2	Li_2CO_3 (1.5 equiv)	i-Bu	4	33 : 22 : 10
10	THF/EtOH 4:1	0.04	2	Na_2CO_3 (1.5 equiv)	Et	24	74 : 4 : 1
11	THF/EtOH 4:1	0.04	2	K_2CO_3 (1.5 equiv)	Et	44	72 : 3 : 2
12	THF/EtOH 4:1	0.04	2	NaOH (1.5 equiv)	Et	14	65 : 2 : 1
13	THF/EtOH 4:1	0.04	2	KOH (1.5 equiv)	Et	24	56 : 2 : 1
14	THF/EtOH 4:1	0.08	2	NaOH (1.5 equiv)	Et	18	57 : 1 : 1
15	THF/EtOH 4:1	0.02	2	NaOH (1.5 equiv)	Et	24	65 : 2 : 3

[a] To a mixture of compound **2a** (0.1 mmol) and the indicated base or additive (0.07-0.3 mmol, 0.7-3 equiv), in the corresponding solvent or solvent mixture, MMPP (0.05-0.2 mmol, 0.5-2 equiv) is added. The reaction is stirred at room temperature for the indicated time.
[b] Yield determined by ^1H NMR analysis of crude reaction mixture using tetrachloroethane as internal standard.
[c] Yield obtained after isolation by column chromatography.

Experimental Procedures and Compounds Characterization

General procedure for the one-pot synthesis of the epoxides 3a-r

To a mixture of aldehyde **1** (0.22 mmol, 1.1 equiv) and (phenylsulfonyl)acetonitrile (37.0 mg, 0.20 mmol) in ethanol (1.0 mL), diethylamine (4.0 µL, 0.04 mmol, 20 mol%) is added and the reaction mixture is stirred at room temperature for 2-6.5 h. After this time, nitromethane (56 µL, 1.0 mmol, 5.0 equiv) is added and the reaction is stirred at room temperature for 1-42 h. Then, the reaction mixture is diluted by adding 2-methyltetrahydrofuran (4.0 mL), followed by sodium hydroxide (12.0 mg, 0.30 mmol, 1.5 equiv) and magnesium bis(monoperoxyphthalate) hexahydrate (MMPP) (80%, 278 mg, 0.45 mmol, 2.25 equiv). The reaction is stirred at room temperature for 22-33 h. Then, the reaction is poured into an aqueous saturated $NaHCO_3$ solution (40 mL) and extracted with diethyl-ether (40 mL). The organic layer is washed with additional aqueous saturated $NaHCO_3$ solution (40 mL), dried with anhydrous sodium sulfate, and filtered. The solvent is removed by rotary evaporation. The residue obtained is purified by column chromatography using silica gel as the stationary phase and a mixture of PE/ethyl acetate as the eluent (pentane/Et_2O for the product **3q**), affording pure the corresponding racemic terminal epoxides **3** in 24-73% yield. Every step in the one-pot sequence was monitored by TLC using a mixture of PE/ethyl acetate (9:1 and 7:3) as eluent and a combination of UV light and phosphomolybdic acid stain for visualization. Due to the volatility of the epoxide **3q**, this reaction was carried out at 0.5 mmol scale. For the synthesis of the epoxide **3q**, the oxidation step was carried out at -20 °C. For the synthesis of epoxide **3r**, due to the low solubility of the alkene intermediate after Knoevenagel condensation, the dilution with 2-methyltetrahydrofuran (4.0 mL) was performed before the addition of nitrometane.

Ethyl 2-phenyloxirane-2-carboxylate (*rac*-3a)[6]

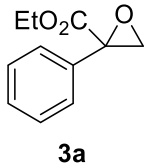

3a

Column chromatography eluent: PE/ethyl acetate, 99:1 to 24:1. Colorless oil, 23.4 mg, 61% yield. **^{1}H NMR:** (600 MHz, $CDCl_3$) δ 7.52 – 7.49 (m, 2H), 7.39 – 7.34 (m, 3H), 4.25 (q, J = 7.1 Hz, 2H), 3.42 (d, J = 6.4 Hz, 1H), 2.96 (d, J = 6.4 Hz, 1H), 1.28 (t, J = 7.1 Hz, 3H). **^{13}C {^{1}H} NMR:** (151 MHz, $CDCl_3$) δ 169.3, 134.5, 128.6, 128.3, 127.3, 62.1, 58.2, 54.2, 14.1. **HRMS (MALDI-FT ICR)** m/z: $[M+Na]^+$ calculated for $C_{11}H_{12}NaO_3$: 215.0679, found: 215.0698.

Ethyl 2-(4-methylphenyl)oxirane-2-carboxylate (*rac*-3b)

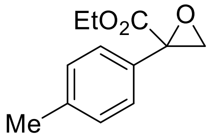

3b

Column chromatography eluent: PE/ethyl acetate, 99:1 to 24:1. Colorless oil, 22.7 mg, 55% yield. **^{1}H NMR:** (600 MHz, $CDCl_3$) δ 7.39 (pd, J = 8.0 Hz, 2H), 7.18 (pd, J = 8.0 Hz, 2H), 4.27–4.23 (m, 2H), 3.40 (d, J = 6.4 Hz, 1H), 2.95 (d, J = 6.4 Hz, 1H), 2.36 (s, 3H), 1.28 (t, J = 7.1 Hz, 3H). **^{13}C {^{1}H} NMR:** (151 MHz, $CDCl_3$) δ 169.5, 138.6, 131.6, 129.1, 127.3, 62.2, 58.2, 54.2, 21.4, 14.2. **HRMS (MALDI-FT ICR)** m/z: $[M+K]^+$ calculated for $C_{12}H_{14}KO_3$: 245.0575, found: 245.0588.

Ethyl 2-(4-methoxyphenyl)oxirane-2-carboxylate (*rac*-3c)

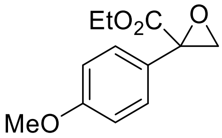

3c

Column chromatography eluent: PE/ethyl acetate, 99:1 to 24:1. Pale yellow oil, 20.9 mg, 47% yield. **^{1}H NMR:** (600 MHz, $CDCl_3$) δ 7.43–7.41 (m, 2H), 6.90–6.89 (m, 2H), 4.25 (q, J = 7.1 Hz, 2H), 3.81 (s, 3H), 3.38 (d, J = 6.3 Hz, 1H), 2.96 (d, J = 6.3 Hz, 1H), 1.28 (t, J = 7.1 Hz, 3H). **^{13}C {^{1}H} NMR:** (151 MHz, $CDCl_3$) δ 169.6, 159.9, 128.7, 126.6, 113.8, 62.2, 58.0, 55.4, 54.2, 14.2. **HRMS (MALDI-FT ICR)** m/z: $[M+Na]^+$ calculated for $C_{12}H_{14}NaO_4$: 245.0784, found: 245.0784.

Ethyl 2-(4-fluorophenyl)oxirane-2-carboxylate (*rac*-3d)

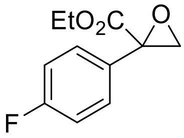

3d

Column chromatography eluent: PE/ethyl acetate, 99:1 to 24:1. Colorless oil, 23.1 mg, 55% yield. **^{1}H NMR:** (600 MHz, $CDCl_3$) δ 7.51 – 7.47 (m, 2H), 7.08 – 7.04 (m, 2H), 4.25 (q, J = 7.1 Hz, 2H), 3.41 (d, J = 6.4 Hz, 1H), 2.93 (d, J = 6.4 Hz, 1H), 1.28 (t, J = 7.1 Hz, 3H). **^{13}C {^{1}H} NMR:** (151 MHz, $CDCl_3$) δ 169.2, 162.9 (d, $^1J_{CF}$ = 247.3 Hz), 130.4 (d, $^4J_{CF}$ = 3.2 Hz), 129.3 (d, $^3J_{CF}$ = 8.0 Hz), 115.4 (d, $^2J_{CF}$ = 22.0 Hz), 62.3, 57.7, 54.4, 14.2. **^{19}F {^{1}H} NMR:** (565 MHz, $CDCl_3$) δ -113.0. **HRMS (MALDI-FT ICR)** m/z: $[M+Na]^+$ calculated for $C_{11}H_{11}FNaO_3$: 233.0584, found: 233.0611.

Ethyl 2-(4-chlorophenyl)oxirane-2-carboxylate (*rac*-3e)[7]

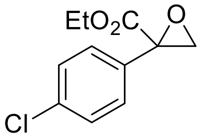

3e

Column chromatography eluent: PE/ethyl acetate, 99:1 to 24:1. Pale-yellow oil, 16.8 mg, 37% yield. **^{1}H NMR:** (400 MHz, $CDCl_3$) δ 7.45 (pd, *J* = 8.5 Hz, 2H), 7.34 (pd, *J* = 8.5 Hz, 2H), 4.25 (q, *J* = 7.1 Hz, 2H), 3.42 (d, *J* = 6.4 Hz, 1H), 2.91 (d, *J* = 6.4 Hz, 1H), 1.28 (t, *J* = 7.1 Hz, 3H). **^{13}C {^{1}H} NMR:** (101 MHz, $CDCl_3$) δ 169.0, 134.7, 133.1, 128.8, 128.6, 62.4, 57.6, 54.5, 14.2. **HRMS (ESI)** m/z: $[M+H]^+$ calculated for $C_{11}H_{12}ClO_3$: 227.0469, found: 227.0465.

Ethyl 2-(4-bromophenyl)oxirane-2-carboxylate (*rac*-3f)

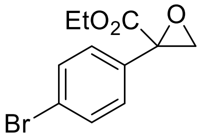

3f

Column chromatography eluent: PE/ethyl acetate, 99:1 to 24:1. Colorless oil, 24.4 mg, 47% yield. **^{1}H NMR:** (300 MHz, $CDCl_3$) δ 7.50 (d, *J* = 8.4 Hz, 2H), 7.39 (d, *J* = 8.1 Hz, 2H), 4.25 (q, *J* = 7.1 Hz, 2H), 3.42 (d, *J* = 6.3 Hz, 1H), 2.90 (d, *J* = 6.3 Hz, 1H), 1.28 (t, *J* = 7.1 Hz, 2H). **^{13}C {^{1}H} NMR:** (62.5 MHz, $CDCl_3$) δ 168.9, 133.6, 131.5, 129.1, 122.9, 62.4, 57.7, 54.4, 14.2. **HRMS (MALDI-FT ICR)** m/z: $[M+Na]^+$ calculated for $C_{11}H_{11}BrNaO_3$: 292.9784 and 294,9764 found: 292.9793 and 294.9774.

Ethyl 2-(3-bromophenyl)oxirane-2-carboxylate (*rac*-3g)

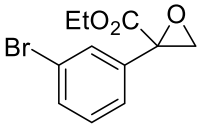

3g

Column chromatography eluent: PE/ethyl acetate, 99:1 to 24:1. Colorless oil, 24.9 mg, 43% yield. **^{1}H NMR:** (400 MHz, $CDCl_3$) δ 7.67 (s, 1H), 7.49 – 7.45 (m, 2H), 7.24 (t, *J* = 8.0 Hz, 1H), 4.26 (q, *J* = 7.1 Hz, 2H), 3.42 (d, *J* = 6.4 Hz, 1H), 2.91 (d, *J* = 6.4 Hz, 1H), 1.29 (t, *J* = 7.1 Hz, 3H). **^{13}C {^{1}H} NMR:** (75 MHz, $CDCl_3$) δ 168.8, 136.8, 131.8, 130.4, 129.9, 126.1, 122.4, 62.5, 57.5, 54.5, 14.2. **HRMS (MALDI-FT ICR)** m/z: $[M+Na]^+$ calculated for $C_{11}H_{11}BrNaO_3$: 292.9784 and 294.9764, found: 292.9819 and 294.9797.

Ethyl 2-(2-fluorophenyl)oxirane-2-carboxylate (*rac*-3h)

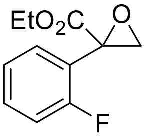

3h

Column chromatography eluent: PE/ethyl acetate, 99:1 to 49:1. Colorless oil, 15.1 mg, 37% yield. **^{1}H NMR:** (400 MHz, $CDCl_3$) δ 7.46 – 7.33 (m, 2H), 7.18 – 7.12 (m, 1H), 7.12 – 7.05 (m, 1H), 4.26 – 4.21 (m, 2H), 3.52 (d, *J* = 6.4 Hz, 1H), 3.04 (d, *J* = 6.4 Hz, 1H), 1.25 (t, *J* = 7.1 Hz, 3H). **^{13}C {^{1}H} NMR:** (62.5 MHz, $CDCl_3$) δ 168.8, 161.4 (d, $^1J_{CF}$ = 248.9 Hz), 130.7 (d, $^3J_{CF}$ = 8.0 Hz), 128.9, 124.2, 122.9 (d, $^2J_{CF}$ = 15.7 Hz), 115.4 (d, $^2J_{CF}$ = 20.6 Hz), 62.4, 55.0, 53.0, 14.1. **^{19}F {^{1}H} NMR:** (376 MHz, $CDCl_3$) δ -115.2. **HRMS (MALDI-FT ICR)** m/z: $[M+H]^+$ calculated for $C_{11}H_{12}FO_3$: 211.0765, found: 211.0758.

Ethyl 2-(4-nitrophenyl)oxirane-2-carboxylate (*rac*-3i)

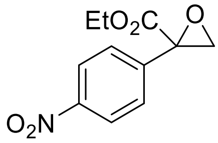

3i

Column chromatography eluent: PE/ethyl acetate, 99:1 to 24:1. Pale yellow oil, 34.6 mg, 73% yield. **^{1}H NMR:** (400 MHz, $CDCl_3$) δ 8.23 (pd, *J* = 9.2 Hz, 2H), 7.72 (pd, *J* = 8.8 Hz, 2H), 4.28 (q, *J* = 7.1 Hz, 2H), 3.51 (d, *J* = 6.4 Hz, 1H), 2.91 (d, *J* = 6.4 Hz, 1H), 1.30 (t, *J* = 7.1 Hz, 3H). **^{13}C {^{1}H} NMR:** (62.5 MHz, $CDCl_3$) δ 168.2, 148.1, 141.6, 128.4, 123.5, 62.8, 57.4, 54.9, 14.2. **HRMS (MALDI-FT ICR)** m/z: $[M+H]^+$ calculated for $C_{11}H_{11}NO_5$: 238.0710, found: 238.0723.

Ethyl 2-(2-nitrophenyl)oxirane-2-carboxylate (*rac*-3j)

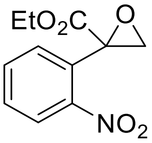

3j

Column chromatography eluent: PE/ethyl acetate, 99:1 to 97:3. Colorless oil, 23.2 mg, 49% yield. **^{1}H NMR:** (600 MHz, $CDCl_3$) δ 8.20 (dd, *J* = 8.4 Hz, *J* = 1.2 Hz, 1H), 7.74 – 7.69 (m, 2H), 7.58 – 7.51 (m, 1H), 4.22 (dq, *J* = 10.8, 7.1 Hz, 1H), 4.15 (dq, *J* = 10.8, 7.1 Hz, 1H), 3.78 (d, *J* = 6.2 Hz, 1H), 2.97 (d, *J* = 6.2 Hz, 1H), 1.20 (t, *J* = 7.1 Hz, 3H). **^{13}C {^{1}H} NMR:** (151 MHz, $CDCl_3$) δ 167.8, 148.1, 134.2, 131.5, 129.7, 129.1, 124.8, 62.6, 57.8, 54.6, 14.0. **HRMS (MALDI-FT ICR)** m/z: $[M+H]^+$ calculated for $C_{11}H_{11}NO_5$: 238.0710, found: 238.0719.

Ethyl 2-(4-(trifluoromethyl)phenyl)oxirane-2-carboxylate (*rac*-3k)

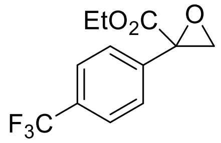

3k

Column chromatography eluent: PE/ethyl acetate, 99:1 to 49:1. Colorless oil, 23.9 mg, 46% yield. **^{1}H NMR:** (600 MHz, $CDCl_3$) δ 7.66 – 7.62 (m, 4H), 4.29 – 4.25 (m, 2H), 3.47 (d, J = 6.4 Hz, 1H), 2.91 (d, J = 6.4 Hz, 1H), 1.29 (t, J = 7.1 Hz, 3H). **^{13}C {^{1}H} NMR:** (151 MHz, $CDCl_3$) δ 168.7, 138.5, 130.9 (q, $^2J_{CF}$ = 32.5 Hz), 127.8, 125.3 (q, $^3J_{CF}$ = 3.7 Hz), 124.1 (q, $^1J_{CF}$ = 272.0 Hz), 62.5, 57.6, 54.6, 14.2. **^{19}F {^{1}H} NMR:** (565 MHz, $CDCl_3$) δ -62.7. **HRMS (MALDI-FT ICR)** m/z: $[M+Na]^+$ calculated for $C_{12}H_{11}F_3NaO_3$: 283.0552, found: 283.0553.

Ethyl 2-(4-(methoxycarbonyl)phenyl)oxirane-2-carboxylate (*rac*-3l)

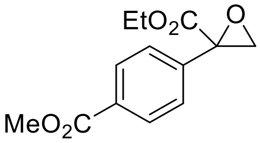

3l

Column chromatography eluent: PE/ethyl acetate, 99:1 to 24:1. Colorless wax, 20.0 mg, 40% yield. **^{1}H NMR:** (600 MHz, $CDCl_3$) δ 8.03 (pd, J = 8.4 Hz, 2H), 7.59 (pd, J = 8.4 Hz, 2H), 4.26 (q, J = 7.1 Hz, 2H), 3.92 (s, 3H), 3.46 (d, J = 6.4 Hz, 1H), 2.92 (d, J = 6.4 Hz, 1H), 1.28 (t, J = 7.1 Hz, 2H). **^{13}C {^{1}H} NMR:** (151 MHz, $CDCl_3$) δ 168.7, 166.8, 139.4, 130.4, 129.6, 127.3, 62.5, 57.9, 54.6, 52.3, 14.2. **HRMS (MALDI-FT ICR)** m/z: $[M+Na]^+$ calculated for $C_{13}H_{14}NaO_5$: 373.0733, found: 373.0789.

Ethyl 2-(naphthalen-2-yl)oxirane-2-carboxylate (*rac*-3m)

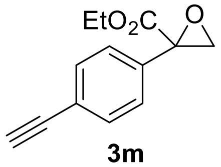

3m

Column chromatography eluent: PE/ethyl acetate, 99:1 to 24:1. Pale-yellow wax, 29.4 mg, 68% yield. **^{1}H NMR:** (600 MHz, $CDCl_3$) δ 7.51–7.46 (m, 4H), 4.25 (q, J = 7.1 Hz, 2H), 3.43 (d, J = 6.4 Hz, 1H), 3.10 (s, 1H), 2.92 (d, J = 6.4 Hz, 1H), 1.28 (t, J = 7.1 Hz, 3H). **^{13}C {^{1}H} NMR:** (151 MHz, $CDCl_3$) δ 168.9, 135.2, 132.1, 127.3, 122.5, 83.3, 78.1, 62.4, 57.9, 54.5, 14.2. **HRMS (MALDI-FT ICR)** m/z: $[M+Na]^+$ calculated for $C_{13}H_{12}NaO_3$: 239.0679, found: 239.0685.

Ethyl 2-(4-bromo-3-nitrophenyl)oxirane-2-carboxylate (*rac*-3n)

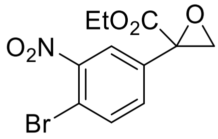

3n

Column chromatography eluent: PE/ethyl acetate, 99:1 to 19:1. Pale yellow solid, 28.8 mg, 46% yield. **mp** = 32−36 °C. **^{1}H NMR:** (600 MHz, $CDCl_3$) δ 8.04 (d, *J* = 2.1 Hz, 1H), 7.74 (d, *J* = 8.4 Hz, 1H), 7.62 (dd, *J* = 8.4, 2.1 Hz, 1H), 4.27 (q, *J* = 7.1 Hz, 2H), 3.49 (d, *J* = 6.4 Hz, 1H), 2.90 (d, *J* = 6.4 Hz, 1H), 1.30 (t, *J* = 7.1 Hz, 3H). **^{13}C {^{1}H} NMR:** (151 MHz, $CDCl_3$) δ 167.9, 149.8, 135.9, 135.0, 132.0, 124.7, 114.7, 62.8, 56.6, 55.0, 14.1. **HRMS (MALDI-FT ICR)** m/z: $[M+H]^+$ calculated for $C_{11}H_{11}BrNO_5$: 315.9815 and 317.9796, found 315.9828 and 317.9808.

Ethyl 2-(3,4-dimethylphenyl)oxirane-2-carboxylate (*rac*-3o)

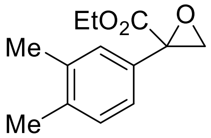

3o

Column chromatography eluent: PE/ethyl acetate, 99:1 to 24:1. Pale-yellow oil, 15.4 mg, 35% yield. **^{1}H NMR:** (300 MHz, $CDCl_3$) δ 7.27–7.23 (m, 2H), 7.13 (d, *J* = 7.8 Hz, 1H), 4.25 (q, *J* = 7.1 Hz, 2H), 3.39 (d, *J* = 6.3 Hz, 1H), 2.96 (d, *J* = 6.3 Hz, 1H), 2.27 (s, 3H), 2.26 (s, 3H), 1.28 (t, *J* = 7.1 Hz, 3H). **^{13}C {^{1}H} NMR:** (62.5 MHz, $CDCl_3$) δ 169.6, 137.3, 136.7, 131.9, 129.6, 128.4, 124.8, 62.1, 58.3, 54.1, 19.9, 19.7, 14.2. **HRMS (MALDI-FT ICR)** m/z: $[M+Na]^+$ calculated for $C_{13}H_{16}NaO_3$: 243.0992, found: 243.0992.

Ethyl 2-(naphthalen-2-yl)oxirane-2-carboxylate (*rac*-3p)

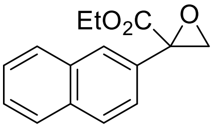

3p

Column chromatography eluent: PE/ethyl acetate, 99:1 to 24:1. Pale-yellow oil, 21.8 mg, 45% yield. **^{1}H NMR:** (600 MHz, $CDCl_3$) δ 7.98 (d, *J* = 1.5 Hz, 1H), 7.89–7.80 (m, 3H), 7.61 (dd, *J* = 8.6 Hz, 1.7 Hz, 1H), 7.54–7.47 (m, 2H), 4.28 (q, *J* = 7.1 Hz, 2H), 3.50 (d, *J* = 6.4 Hz, 1H), 3.05 (d, *J* = 6.4 Hz, 1H), 1.29 (t, *J* = 7.1 Hz, 3H). **^{13}C {^{1}H} NMR:** (151 MHz, $CDCl_3$) δ 169.4, 133.4, 133.0, 132.1, 128.3, 128.1, 127.8, 126.7, 126.5, 126.5, 125.0, 62.3, 58.4, 54.4, 14.2. **HRMS (MALDI-FT ICR)** m/z: $[M+Na]^+$ calculated for $C_{15}H_{14}NaO_3$: 265.0835, found: 265.0843.

Ethyl 2-(furan-3-yl)oxirane-2-carboxylate (*rac*-3q)

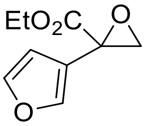

3q

Column chromatography eluent: pentane/Et_2O, 19:1. Colorless oil, 21.9 mg, 24% yield. **^{1}H NMR:** (300 MHz, $CDCl_3$) δ 7.73 (s, 1H), 7.38 (s, 1H), 6.40–6.39 (m, 1H), 4.32–4.25 (m, 2H), 3.38 (d, *J* = 6.4 Hz, 1H), 3.02 (d, *J* = 6.4 Hz, 1H), 1.32 (t, *J* = 7.1 Hz, 3H). **^{13}C {^{1}H} NMR:** (75 MHz, $CDCl_3$) δ 168.9, 143.1, 142.3, 120.1, 109.2, 62.2, 55.4, 52.6, 14.2. **HRMS (MALDI-FT ICR)** m/z: $[M+K]^+$ calculated for $C_9H_{10}KO_4$: 221.0211, found: 221.0200.

Ethyl 2-(1-(phenylsulfonyl)-1H-indol-3-yl)oxirane-2-carboxylate (*rac*-3r)

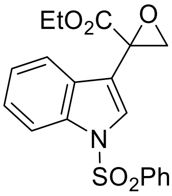

3r

Column chromatography eluent: PE/ethyl acetate, 19:1 to 9:1. Yellow wax, 30.6 mg, 39% yield. **^{1}H NMR:** (300 MHz, $CDCl_3$) δ 8.00–7.57 (m, 3H), 7.82 (s, 1H), 7.63 (d, *J* = 7.9 Hz, 1H), 7.58–7.53 (m, 1H) 7.48–7.43 (m, 2H), 7.36–7.31 (m, 1H), 7.27–7.22 (m, 1H), 4.25 (q, *J* = 7.1 Hz, 2H), 3.45 (d, *J* = 6.4 Hz, 1H), 3.15 (d, *J* = 6.4 Hz, 1H), 1.25 (t, *J* = 7.1 Hz, 3H). **^{13}C {^{1}H} NMR:** (75 MHz, $CDCl_3$) δ 168.8, 138.2, 135.0, 134.2, 129.5, 129.1, 127.0, 126.0, 125.3, 123.7, 120.9, 116.8, 113.7, 62.4, 53.6, 53.3, 14.2. **HRMS (MALDI-FT ICR)** m/z: $[M+Na]^+$ calculated for $C_{19}H_{17}NNaO_5S$: 394.0720, found: 394.0729.

Synthesis of the intermediate 2a

To a mixture of (*E*)-3-phenyl-2-(phenylsulfonyl)acrylonitrile (135 mg, 0.5 mmol, 1.0 equiv) and diethylamine (10.5 µL, 0.1 mmol, 20 mol%) in ethanol (2.5 mL), nitromethane (135 µL, 2.5 mmol, 5 equiv) is added and the reaction mixture is stirred at room temperature. The reaction is monitored by TLC using a mixture of hexane/ethyl acetate 4:1 as eluent. After 3 hours of reaction time, total conversion of the alkene is observed. The solvent is evaporated under reduced pressure, and the residue is purified by column chromatography using silica gel as stationary phase and a mixture hexane/ethyl acetate 4:1 as eluent. The compound **2a** is obtained as a diastereomeric mixture.

4-nitro-3-phenyl-2-(phenylsulfonyl)butanenitrile (2a)

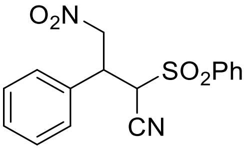

2a

Pale-brown solid, 150.3 mg, 91% yield. dr = 65:35. **^{1}H NMR:** (600 MHz, $CDCl_3$) δ 8.01 (d, J = 7.4 Hz, 1.3H), 7.88 (d, J = 7.4 Hz, 0.7H), 7.79 (t, J = 7.4 Hz, 0.65H), 7.73 (t, J = 7.4 Hz, 0.35H), 7.67–7.64 (m, 1.3H), 7.58–7.55 (m, 0.7H), 7.37–7.31 (m, 5H), 5.34 (dd, J = 13.8 Hz, 4.1 Hz, 0.65H), 5.11 (dd, J = 13.8 Hz, 11.3 Hz, 0.65H), 5.07 (dd, J = 13.8 Hz, 7.1 Hz, 0.35H), 4.87 (dd, J = 13.8 Hz, 7.3 Hz, 0.35H), 4.65–4.61 (m, 0.65H), 4.59 (d, J = 7.0 Hz, 0.35H), 4.40 (dd, J = 14.3 Hz, 7.2 Hz, 0.35H), 4.14 (d, J = 2.9 Hz, 0.65H). **^{13}C {^{1}H} NMR:** (151 MHz, $CDCl_3$) δ 136.0, 135.9, 135.7, 135.6, 134.7, 133.0, 130.1, 129.9, 129.8, 129.7, 129.6, 129.6, 129.5, 128.5, 127.5, 112.6, 112.1, 76.8, 75.5, 60.4, 59.3, 41.6, 39.9. **HRMS (MALDI-FT ICR)** m/z: $[M+K]^+$ calculated for $C_{16}H_{14}N_2KO_4S$: 369.0306, found: 369.0364.

One-pot synthesis of the intermediate 2r

To a mixture of 1-(phenylsulfonyl)-1*H*-indole-3-carbaldehyde (**1r**) (285 mg, 1.0 mmol, 1.0 equiv) and (phenylsulfonyl)acetonitrile (185 mg, 1.0 mmol) in ethanol (10 mL), diethylamine (41 µL, 0.4 mmol, 40 mol%) is added and the reaction is stirred at room temperature for 24 hours. After this time a white suspension is obtained. The reaction mixture is diluted with tetrahydrofuran (40 mL). Nitromethane (0.28 mL, 5.0 mmol, 5.0 equiv) is added and the reaction is stirred at room temperature for 6.5 h. An orange solution is obtained. The solvent is evaporated under reduced pressure, and the residue is purified by column chromatography using silica gel as stationary phase and a gradient of hexane/ethyl acetate 9:1 to 7:3 as eluent. The compound **2r** is obtained as a diastereomeric mixture.

4-Nitro-2-(phenylsulfonyl)-3-(1-(phenylsulfonyl)-1H-indol-3-yl)butanenitrile (2r)

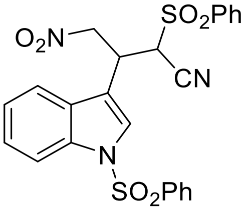

2r

Pale yellow solid, 341.4 mg, 67% yield. dr = 65:35. **^{1}H NMR:** (300 MHz, $CDCl_3$) δ 8.05 – 7.27 (m, 15H), 5.42 (dd, *J* = 13.5, 4.1 Hz, 0.65H), 5.14 (dd, *J* = 13.5, 11.2 Hz, 0.65H), 5.04 – 4.89 (m, 1.35H), 4.76 – 4.67 (m, 0.7H), 4.17 (d, *J* = 2.8 Hz, 0.65H). **^{13}C {^{1}H} NMR:** (75 MHz, $CDCl_3$) δ 137.6, 137.4, 136.2, 125.8, 135.8, 135.5, 135.2, 134.6, 134.4, 134.3, 130.2, 129.7, 129.6, 129.6, 129.5, 129.5, 128.8, 128.0, 127.0, 127.0, 126.3, 126.1, 125.7, 124.6, 124.4, 124.2, 119.2, 118.3, 116.1, 114.3, 114.2, 114.0, 112.9, 111.7, 75.9, 74.7, 59.2, 58.7, 33.2, 32.0. **HRMS (MALDI-FT ICR)** m/z: $[M+Na]^+$ calculated for $C_{24}H_{19}N_3NaO_6S_2$: 532.0608, found: 532.0652.

Opening of the epoxide 3a with *p*-anisidine

To a solution of the epoxide **3a** (19.2 mg, 0.1 mmol) in ethanol (0.5 mL), *p*-anisidine (25.1 mg, 0.2 mmol, 2.0 equiv) is added and the reaction is heated in an oil bath and stirred at 60 ºC. The reaction is monitored by TLC using a mixture of hexane/ethyl acetate 8:2 as the eluent and phosphomolybdic acid as the TLC stain. After 24 hours, total conversion of the epoxide is observed. The solvent is evaporated under reduced pressure and the residue purified by column chromatography using silica gel as stationary phase and a gradient of hexane/ethyl acetate 9:1 to 17:3 as eluent. After the column, the pure racemic *p*-methoxyphenyl protected *β*-amino ester **6a** is obtained.

Ethyl 2-hydroxy-3-((4-methoxyphenyl)amino)-2-phenylpropanoate (*rac*-6a)

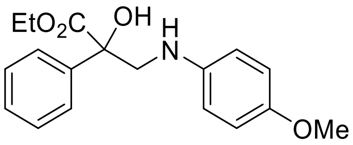

6a

Yellow oil, 27.8 mg, 88% yield. **^{1}H NMR:** (600 MHz, $CDCl_3$) δ 7.67–7.65 (m, 2H), 7.40–7.38 (m, 2H), 7.35–7.32 (m, 1H), 6.79–6.76 (m, 2H), 6.70–6.67 (m, 2H), 4.20 (q, *J* = 7.1 Hz, 2H), 4.12 (s, 1H), 3.99 (d, *J* = 12.4 Hz, 1H), 3.88 (bs, 1H), 3.75 (s, 3H), 3.37 (d, *J* = 12.4 Hz, 1H), 1.23 (t, *J* = 7.1 Hz, 3H). **^{13}C {^{1}H} NMR:** (151 MHz, $CDCl_3$) δ 174.3, 152.8, 142.2, 139.8, 128.6, 128.3, 125.6, 115.6, 114.9, 78.8, 62.9, 55.9, 53.5, 14.1. **HRMS (MALDI-FT ICR)** m/z: $[M+H]^+$ calculated for $C_{18}H_{22}NO_4$: 316.1543, found: 316.1553.

Synthesis of the indole-derived azide 7r

To a solution of the indole-derived epoxide **3r** (37.1 mg, 0.1 mmol) in ethanol (1.0 mL), sodium azide (19.5 mg, 0.3 mmol, 3.0 equiv) and ammonium chloride (16.0 mg, 0.3 mmol, 3.0 equiv) are added and the reaction mixture is heated in an oil bath and stirred at 65 ºC for 19 hours. After this

time, the solvent is evaporated under reduced pressure. The residue is suspended in water (20 mL) and extracted with ethyl acetate (3 x 20 mL). The organic layers are collected, washed with brine (2 x 20 mL) and dried with anhydrous sodium sulfate. After filtration, the solvent is evaporated under reduced pressure and the residue purified by column chromatography using silica gel as the stationary phase and a gradient of hexane/ethyl acetate 19:1 to 17:3 as the eluent. After column, the pure racemic indole-derived azide **7r** is obtained.

Ethyl 3-azido-2-hydroxy-2-(1-(phenylsulfonyl)-1H-indol-3-yl)propanoate (*rac*-7r)

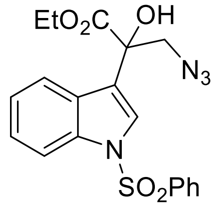

7r

Colorless oil, 29.0 mg, 70% yield. **^{1}H NMR:** (600 MHz, $CDCl_3$) δ 7.98 (d, *J* = 8.4 Hz, 1H), 7.90 (d, *J* = 7.6 Hz, 2H), 7.81 (d, *J* = 8.0 Hz, 1H), 7.70 (s, 1H), 7.56 (t, *J* = 7.5 Hz, 1H), 7.46 (t, *J* = 7.9 Hz, 2H), 7.33 (t, *J* = 7.8 Hz, 1H), 7.25 (t, *J* = 7.5 Hz, 1H), 4.36 (dq, *J* = 10.8, 7.1 Hz, 1H), 4.29 (dq, *J* = 10.8, 7.1 Hz, 1H), 4.18 (s, 1H), 3.91 (d, *J* = 12.5 Hz, 1H), 3.78 (d, *J* = 12.5 Hz, 1H), 1.29 (t, *J* = 7.1 Hz, 3H). **^{13}C {^{1}H} NMR:** (151 MHz, $CDCl_3$) δ 172.4, 138.1, 135.6, 134.2, 129.6, 127.9, 127.0, 125.2, 124.5, 123.6, 121.8, 120.5, 113.8, 77.0, 63.6, 57.5, 14.2. **HRMS (MALDI-FT ICR)** m/z: $[M+Na]^+$ calculated for $C_{19}H_{18}N_4NaO_5S$: 437.0890, found: 437.0932.

Multi-step synthesis of the *N*-Boc protected *β*-amino ester 8a

The azidation step was carried out following a slightly modified procedure reported in the literature:[8] to a solution of epoxide **3a** (19.2 mg, 0.1 mmol) in ethanol (0.4 mL), sodium azide (19.5 mg, 0.3 mmol, 3.0 equiv) and ammonium chloride (16.0 mg, 0.3 mmol, 3.0 equiv) are added and the reaction mixture is heated in an oil bath and stirred at 65 ºC for 16 hours. After this time, the solvent is removed in the evaporator. The residue is suspended in water (20 mL) and extracted with ethyl acetate (3 x 20 mL). The organic layers are collected, washed with brine (2 x 20 mL) and dried over anhydrous sodium sulfate. After filtration, the solvent is evaporated under reduced pressure. Without further purification, the resulting yellow oil obtained is dissolved in dry ethyl acetate (1 mL). Di-*tert*-butyl dicarbonate (44.1 mg, 0.2 mmol, 2.0 equiv) and palladium (10% on carbon) (10.6 mg, 0.01 mmol, 10 mol%) are added. The flask is equipped with a three-way adapter connected to a balloon filled with hydrogen. The solvent is frozen using a cooling bath of liquid nitrogen, and the air in the flask is replaced by hydrogen by three cycles of vacuum-hydrogen.

Then, the cooling bath is removed, and the reaction mixture is stirred at room temperature for 5 hours. After this time, the reaction is diluted with chloroform (10 mL) and filtered over celite. The solvent is evaporated under reduced pressure, and the racemic *N*-Boc protected *β*-amino ester **8a** is purified by column chromatography (eluent: gradient of hexane/ethyl acetate 19:1 to 4:1).

Ethyl 3-((*tert*-butoxycarbonyl)amino)-2-hydroxy-2-phenylpropanoate (*rac*-8a)

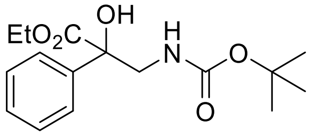

8a

White solid, 22.3 mg, 72% yield. **mp** = 100–102 °C. **^{1}H NMR:** (600 MHz, $CDCl_3$) δ 7.61 (d, *J* = 7.8 Hz, 2H), 7.37–7.34 (m, 2H), 7.32–7.29 (m, 1H), 5.02 (bs, 1H), 4.30–4.25 (m, 1H), 4.24 (bs, 1H), 4.22–4.10 (m, 2H), 3.44 (dd, *J* = 13.8, 3.6 Hz, 1H), 1.41 (s, 9H), 1.28 (t, *J* = 7.1 Hz, 3H). **^{13}C {^{1}H} NMR:** (151 MHz, $CDCl_3$) δ 173.9, 156.2, 139.2, 128.5, 128.3, 125.7, 79.8, 78.8, 62.9, 48.5, 28.4, 14.1. **HRMS (MALDI-FT ICR)** m/z: $[M+Na]^+$ calculated for $C_{16}H_{23}NNaO_5$: 332.1468, found: 332.1475.

Multi-step synthesis of the indole-derived *N*-Boc protected *β*-amino ester 8r

To a solution of indole-derived epoxide **3r** (37.1 mg, 0.1 mmol) in ethanol (1.0 mL), sodium azide (19.5 mg, 0.3 mmol, 3.0 equiv) and ammonium chloride (16.0 mg, 0.3 mmol, 3.0 equiv) are added and the reaction mixture is heated in an oil bath and stirred at 65 ºC for 16 hours. After this time, the solvent is evaporated under reduced pressure. The residue obtained is suspended in water (20 mL) and extracted with ethyl acetate (3 x 20 mL). The organic layers are collected, washed with brine (2 x 20 mL) and dried with anhydrous sodium sulfate. After filtration, the solvent is evaporated under reduced pressure. Without further purification, the crude is directly dissolved in dry ethyl acetate (1 mL) and di-*tert*-butyl dicarbonate (44.1 mg, 0.2 mmol, 2.0 equiv) and palladium (10% on carbon) (10.6 mg, 0.01 mmol, 10 mol%) are added. The flask is equipped with a three-way adapter connected to a balloon filled with hydrogen. The solvent is frozen using a cooling bath of liquid nitrogen, and the air in the flask is replaced by hydrogen by three cycles of vacuum-hydrogen. Then, the cooling bath is removed, and the reaction mixture is stirred at room temperature for 5 hours. After this time, the reaction is diluted with chloroform (15 mL) and filtered over celite. The solvent is evaporated under reduced pressure and the remaining crude purified by column chromatography, using silica gel as the stationary phase and a gradient of hexane/ethyl

acetate 9:1 to 4:1 as the eluent. After the column, the pure racemic indole-derived *N*-Boc protected *β*-amino ester **8r** is obtained.

Ethyl 3-((*tert*-butoxycarbonyl)amino)-2-hydroxy-2-(1-(phenylsulfonyl)-1H-indol-3-yl) propanoate (*rac*-8r)

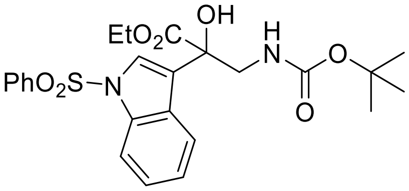

8r

Colorless oil, 32.2 mg, 66% yield. **^{1}H NMR:** (600 MHz, $CDCl_3$) δ 7.97 (d, *J* = 8.4 Hz, 1H), 7.89 (d, *J* = 7.6 Hz, 2H), 7.84 (d, *J* = 7.9 Hz, 1H), 7.73 (s, 1H), 7.54 (t, *J* = 7.5 Hz, 1H), 7.44 (t, *J* = 7.9 Hz, 2H), 7.31 (t, *J* = 7.8 Hz, 1H), 7.23 (t, *J* = 7.6 Hz, 1H), 5.03 (bs, 1H), 4.39 (s, 1H), 4.28 – 4.24 (m, 2H), 4.21 – 4.14 (m, 1H), 3.52 (dd, *J* = 13.6, 2.9 Hz, 1H), 1.42 (s, 9H), 1.24 (t, *J* = 7.1 Hz, 3H). **^{13}C {^{1}H} NMR:** (151 MHz, $CDCl_3$) δ 173.3, 156.2, 138.2, 135.6, 134.1, 129.5, 128.1, 127.0, 125.0, 124.6, 123.5, 122.0, 121.3, 113.7, 80.1, 76.5, 63.2, 47.6, 28.4, 14.1. **HRMS (MALDI-FT ICR)** m/z: $[M+Na]^+$ calculated for $C_{24}H_{28}N_2NaO_7S$: 511.1509, found: 511.1558.

Opening of the epoxide 3a with 2-(benzylamino)ethan-1-ol

The following procedure was based on a similar reaction reported in the literature:[9] To a solution of the epoxide **3a** (19.2 mg, 0.1 mmol) in dry ethanol (0.5 mL), 2-(benzylamino)ethan-1-ol (18 μL, 0.12 mmol, 1.2 equiv) is added and the reaction is heated in an oil bath and stirred at 60 °C. The reaction is monitored by TLC using a mixture of hexane/ethyl acetate 8:2 as eluent and phosphomolybdic acid as a TLC stain. After 28 hours, total conversion of the epoxide is observed. The solvent is removed by rotatory evaporation and the residue purified by column chromatography using silica gel as stationary phase and a gradient of hexane/ethyl acetate 4:1 to 3:2 as eluent. After the column, the pure racemic *β*-amino ester is obtained.

Ethyl 3-(benzyl(2-hydroxyethyl)amino)-2-hydroxy-2-phenylpropanoate

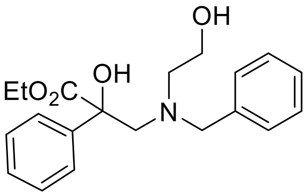

Colorless oil, 24.4 mg, 71% yield. **^{1}H NMR:** (600 MHz, $CDCl_3$) δ 7.65 – 7.63 (m, 2H), 7.37 – 7.34 (m, 2H), 7.32 – 7.23 (m, 6H), 4.59 (bs, 1H), 4.25 (dq, *J* = 10.8, 7.1 Hz, 1H), 4.15 (dq, *J* = 10.8, 7.1 Hz, 1H), 3.89 (d, *J* = 13.9 Hz, 1H), 3.76 (d, *J* = 13.9 Hz, 1H), 3.57 – 3.47 (m, 3H), 3.05 (d, *J* = 14.1 Hz, 1H), 2.79 (ddd, *J* = 13.4, 7.1, 4.2 Hz, 1H), 2.70 (ddd, *J* = 13.5, 5.4, 4.0 Hz, 1H), 2.52 (s, 1H), 1.23 (t, *J* = 7.1 Hz, 3H). **^{13}C {^{1}H} NMR:** (151 MHz, $CDCl_3$) δ 174.6, 140.4, 139.1, 128.9, 128.5, 128.4, 128.0, 127.3, 125.7, 79.6, 63.4, 62.6, 61.0, 60.2, 57.6, 14.1. **HRMS (MALDI-FT ICR)** m/z: $[M+H]^+$ calculated for $C_{20}H_{26}NO_4$: 344.1856, found: 344.1875.

Synthesis of the morpholine derivative 9a

The following procedure was based on a similar reaction reported in the literature:[9] To a solution of (+/-)-ethyl 3-(benzyl(2-hydroxyethyl)amino)-2-hydroxy-2-phenylpropanoate (34.3 mg, 0.1 mmol), sodium hydroxide (16.0 mg, 0.4 mmol, 4.0 equiv) and triethylamine (2.8 μL, 0.02 mmol, 20 mol%) in dry dioxane (0.4 mL), a solution of *p*-toluenesulfonyl chloride (24.1 mg, 0.125 mmol, 1.25 equiv) in dry dioxane (0.6 mL) is added and the reaction is stirred at room temperature. The reaction is monitored by TLC using mixtures of hexane/ethyl acetate 9:1 and 6:4 as eluents in combination with UV light for visualization. After 6.5 hours, total conversion of the starting material is observed. The reaction mixture is poured over a saturated $NaHCO_3$ aqueous saturated solution (30 mL) and extracted with chloroform (3 x 20 mL). The organic layers are collected and dried with anhydrous sodium sulfate. After filtration, the solvent is evaporated under reduced pressure and the residue purified by column chromatography using silica gel as stationary phase and a gradient of hexane/ethyl acetate 19:1 to 4:1 as eluent. After the column, the pure racemic morpholine derivative **9a** is obtained.

Ethyl 4-benzyl-2-phenylmorpholine-2-carboxylate (*rac*-9a)

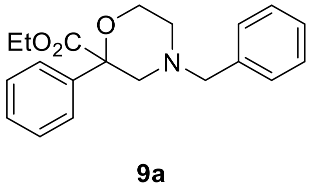

9a

Colorless oil, 24.4 mg, 75% yield (53% overall yield from the epoxide **3a** after two steps). **^{1}H NMR:** (600 MHz, $CDCl_3$) δ 7.51 (d, *J* = 7.8, 2H), 7.34 – 7.28 (m, 8H), 4.24 – 4.19 (m, 2H), 4.09 (td, *J* = 11.1, 2.8 Hz, 1H), 3.95 (dt, *J* = 11.5, 2.8 Hz, 1H), 3.59 (d, *J* = 13.3 Hz, 2H), 3.53 (d, *J* = 13.3 Hz, 1H), 2.67 (d, *J* = 11.1 Hz, 1H), 2.34 (td, *J* = 11.0, 3.5 Hz, 2H), 1.22 (t, *J* = 7.1 Hz, 3H). **^{13}C {^{1}H} NMR:** (151 MHz, $CDCl_3$) δ 171.6, 139.2, 137.7, 129.1, 128.4, 128.3, 128.1, 127.3, 125.4,

80.3, 64.1, 63.1, 61.5, 59.9, 52.8, 14.3. **HRMS (MALDI-FT ICR)** m/z: $[M+H]^+$ calculated for $C_{20}H_{24}NO_3$: 326.1751, found: 326.1761.

Multi-step synthesis of the indole-derived morpholine derivative 9r

To a solution of the indole-derived epoxide **3r** (37.1 mg, 0.1 mmol) in dry ethanol (1.0 mL), 2-(benzylamino)ethan-1-ol (18 µL, 0.12 mmol, 1.2 equiv) is added and the reaction is heated in an oil bath and stirred at 60 ºC. The reaction is monitored by TLC using a mixture of hexane/ethyl acetate 7:3 as the eluent and phosphomolybdic acid as the TLC stain. After 28 hours, total conversion of the epoxide is observed. The solvent is evaporated under reduced pressure, and the crude is used directly in the next step without further purification. The residue is diluted in dry dioxane (0.6 mL). Sodium hydroxide (18.4 mg, 0.46 mmol, 4.6 equiv), triethylamine (2.8 µL, 0.02 mmol, 20 mol%) and a solution of *p*-toluenesulfonyl chloride (28.9 mg, 0.15 mmol, 1.5 equiv) in dry dioxane (0.8 mL) are added and the reaction is stirred at room temperature. The reaction is monitored by TLC using a mixture of hexane/ethyl acetate 7:3 and as the eluent in combination with UV light for visualization. After 6 hours, total conversion of the intermediate is observed. The solvent is removed in the evaporator. The crude product is suspended in brine (30 mL) and extracted with chloroform (3 x 20 mL). The organic layers are collected and dried with anhydrous sodium sulfate. After filtration, the solvent is evaporated under reduced pressure and the residue is purified by column chromatography using silica gel as the stationary phase and a gradient of hexane/ethyl acetate 9:1 to 4:1 as the eluent. After the column, the pure racemic indole-derived morpholine derivative **9r** is obtained.

Ethyl 4-benzyl-2-(1-(phenylsulfonyl)-1H-indol-3-yl)morpholine-2-carboxylate (*rac*-9r)

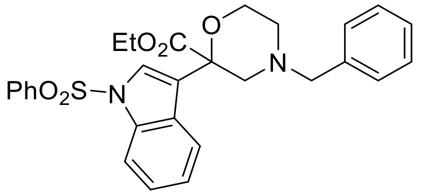

9r

Colorless oil, 23.2 mg, 47% yield. **^{1}H NMR:** (600 MHz, $CDCl_3$) δ 7.93 (d, *J* = 8.4 Hz, 1H), 7.86 – 7.85 (m, 2H), 7.83 (d, *J* = 8.0 Hz, 1H), 7.60 (s, 1H), 7.53 – 7.51 (m, 1H), 7.42 – 7.38 (m, 2H), 7.37 – 7.35 (m, 4H), 7.34 – 7.27 (m, 2H), 7.21 – 7.18 (m, 1H), 4.29 – 4.13 (m, 2H), 4.11 – 4.05 (m, 1H), 3.78 – 3.74 (m, 1H), 3.61 – 3.53 (m, 2H), 3.44 – 3.34 (m, 1H), 2.80 – 2.72 (m, 1H), 2.65 – 2.59 (m, 1H), 2.48 – 2.42 (m, 1H), 1.13 (t, *J* = 7.1 Hz, 3H). **^{13}C {^{1}H} NMR:** (151 MHz, $CDCl_3$) δ 170.6, 138.2, 137.6, 135.3, 134.0, 129.4, 129.2, 128.5, 128.4, 127.5, 127.0, 125.0, 124.9, 123.5, 122.3,

120.6, 113.5, 77.8, 63.7, 63.2, 61.7, 58.5, 53.0, 14.2. **HRMS (MALDI-FT ICR)** m/z: $[M+Na]^+$ calculated for $C_{28}H_{28}N_2NaO_5S$: 527.1611, found: 527.1663.

Scale-Up of the Reaction

One-pot synthesis of the epoxide 3a from benzaldehyde (5 mmol scale)

To a mixture of benzaldehyde **1a** (0.56 mL, 5.5 mmol, 1.1 equiv) and (phenylsulfonyl)acetonitrile (0.92 g, 5.0 mmol) in ethanol (25 mL), diethylamine (104 µL, 1.0 mmol, 20 mol%) is added and the reaction mixture is stirred at room temperature for 4 hours. After this time, nitromethane (1.4 mL, 25 mmol, 5.0 equiv) is added and the reaction is stirred at room temperature for 1.5 hours. Then, the reaction mixture is diluted with 2-methyltetrahydrofuran (100 mL), followed by the addition of sodium hydroxide (0.30 g, 7.5 mmol, 1.5 equiv) and magnesium bis(monoperoxyphthalate) hexahydrate (6.96 g, 11.25 mmol, 2.25 equiv) in this order. The reaction is stirred at room temperature for 23 h. After this time, the solvent is concentrated to a volume of 20 mL (approximately), poured into an aqueous saturated $NaHCO_3$ solution (120 mL) and extracted with diethyl-ether (120 mL). The organic layer is washed with additional aqueous saturated $NaHCO_3$ solution (120 mL), dried with anhydrous sodium sulfate, and filtered. The solvent is removed by rotary evaporation. The residue obtained is purified by column chromatography using silica gel as stationary phase and a mixture of PE/ethyl acetate as eluent (from 99:1 to 98:2), affording pure the racemic terminal epoxide **3a** (424 mg, 44% yield).

One-pot synthesis of the epoxide 3a from the corresponding alkene (6 mmol scale)

To a suspension of (*E*)-3-phenyl-2-(phenylsulfonyl)acrylonitrile[1] (1.62 g, 6.0 mmol) in ethanol (30 mL), nitromethane (1.69 mL, 30.0 mmol) and diethylamine (125 µL, 1.2 mmol, 20 mol%) are added and the reaction mixture is stirred at room temperature for 2.5 hours. After this time, the reaction mixture is diluted with 2-methyltetrahydrofuran (120 mL), followed by the addition of sodium hydroxide (0.36 g, 9.0 mmol, 1.5 equiv) and magnesium bis(monoperoxyphthalate) hexahydrate (8.35 g, 13.5 mmol, 2.25 equiv) in this order. The reaction is stirred at room temperature for 27.5 h. After this time, the solvent is removed under vacuum, poured into an aqueous saturated $NaHCO_3$ solution (120 mL) and extracted with diethyl-ether (120 mL). The organic layer is washed with additional aqueous saturated $NaHCO_3$ solution (120 mL), dried with anhydrous sodium sulfate, and filtered. The solvent is removed by rotary evaporation. The residue obtained is purified by column chromatography using silica gel as stationary phase and a mixture of PE/ethyl acetate as eluent (from 99:1 to 98:2), affording pure racemic terminal epoxide **3a** (563 mg, 49% yield).

One-pot synthesis of the epoxide 3r (1 mmol scale)

To a mixture of aldehyde **1r** (314 mg, 1.1 mmol, 1.1 equiv) and (phenylsulfonyl)acetonitrile (185 mg, 1.0 mmol) in ethanol (10 mL), diethylamine (41 μL, 0.4 mmol, 40 mol%) is added and the reaction mixture is stirred at room temperature for 23 h. After this time, the reaction mixture is diluted adding 2-methyltetrahydrofuran (40 mL) and nitromethane (280 μL, 5.0 mmol, 5.0 equiv) is added. The reaction is stirred at room temperature for 22 h. Then sodium hydroxide (60.0 mg, 1.5 mmol, 1.5 equiv) and magnesium bis(monoperoxyphthalate) hexahydrate (MMPP) (80%, 1.24 g, 2.0 mmol, 2.0 equiv) in this order. The reaction is stirred at room temperature for 24 h. After this time, the reaction is poured into an aqueous saturated $NaHCO_3$ solution (50 mL) and extracted with diethyl-ether (50 mL). The organic layer is washed with saturated aqueous $NaHCO_3$ (50 mL), dried over anhydrous sodium sulfate, and filtered. The solvent is evaporated under reduced pressure. The residue is purified by column chromatography using silica gel as the stationary phase and a mixture of PE/ethyl acetate as the eluent (from 19:1 to 9:1), affording 175 mg of pure racemic epoxide **3r** (47% yield).

Mechanistic Studies

Table S2. Epoxidation of ethyl 2-phenylacrylate **5a** in presence of MMPP.[a]

Entry	MMPP (equiv)	NaOH (equiv)	CH_3NO_2 (equiv)	Et_2NH (equiv)	T (ºC)	Yield (%)
1	1	1.5	-	-	rt	n.r.
2	1	-	-	-	rt	n.r.
3	1	1.5	-	-	50	n.r.
4	1	-	-	-	50	n.r.
5	1	1.5	4	-	30	n.r.
6	1	1.5	4	0.2	30	n.r.

[a] To a solution of ethyl 2-phenylacrylate **5a** (17.6 mg, 0.1 mmol) in a mixture of 2-MeTHF/EtOH 4:1 (2.5 mL), NaOH (6.0 mg, 0.15 mmol, 1.5 equiv), CH_3NO_2 (25 µL, 0.4 mmol, 4.0 equiv), Et_2NH (2 µL, 0.02 mmol, 20 mol%) and MMPP (49.5 mg, 0.1 mmol, 1.0 equiv) are added. The reaction mixture is stirred at the indicated temperature for 22 h. No reaction is observed by TLC (Hx/AcOEt 19:1). n.r. = no reaction.

Analysis of the reaction mixture by HRMS

To a solution of compound **2r** (102 mg, 0.2 mmol) in a mixture of tetrahydrofuran (4.0 mL) and ethanol (1.0 mL), sodium hydroxide (12 mg, 0.3 mmol, 1.5 equiv) and magnesium bis(monoperoxyphthalate) hexahydrate (80%, 247 mg, 0.4 mmol, 2.0 equiv) are added in this order and the reaction is stirred at room temperature. Several aliquots (300 µL) are taken from the reaction mixture at different reaction times (Table S3). Each aliquot is diluted with acetonitrile (100 µL), filtered through a syringe filter (PTFE, 0.45 µm pore size) and analyzed by ESI-HRMS (Figures S1-S5). In the study, only the reaction intermediate **II'** was observed, together with the starting material **2r** and the product **3r**.

Table S3. ESI-HRMS analysis of the reaction mixture (**2r** in presence of NaOH and MMPP).

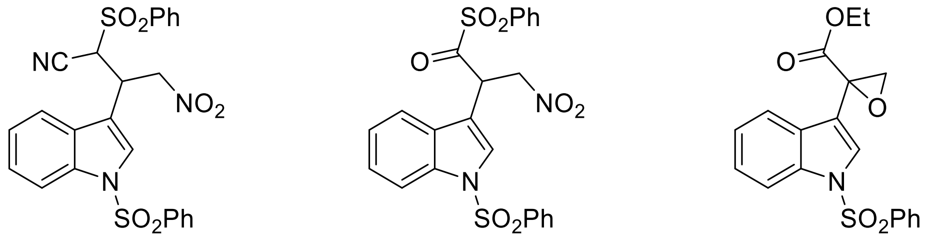

**2r**	**II'**	**3r**
$[M+Na]^+$	$[M+Na]^+$	$[M+Na]^+$
$C_{24}H_{19}N_3O_6S_2Na$	$C_{23}H_{18}N_2O_7S_2Na$	$C_{19}H_{17}NO_5SNa$
**532.0607**	**521.0448**	**394.0720**

Entry	t (min)	m/z **2r** $[M+Na]^+$	m/z **2r** Relative Abundance	m/z **II'** $[M+Na]^+$	m/z **II'** Relative Abundance	m/z **3r** $[M+Na]^+$	m/z **3r** Relative Abundance
1	10	532.0583	16	521.0542	11	394.0700	100
2	30	532.0585	12.5	521.0544	11.5	394.0701	100
3	60	532.0588	11	521.0547	15	394.0704	100
4	120	532.0592	9	521.0549	16	394.0707	100
5	180	532.0585	6	521.0545	16	394.0703	100

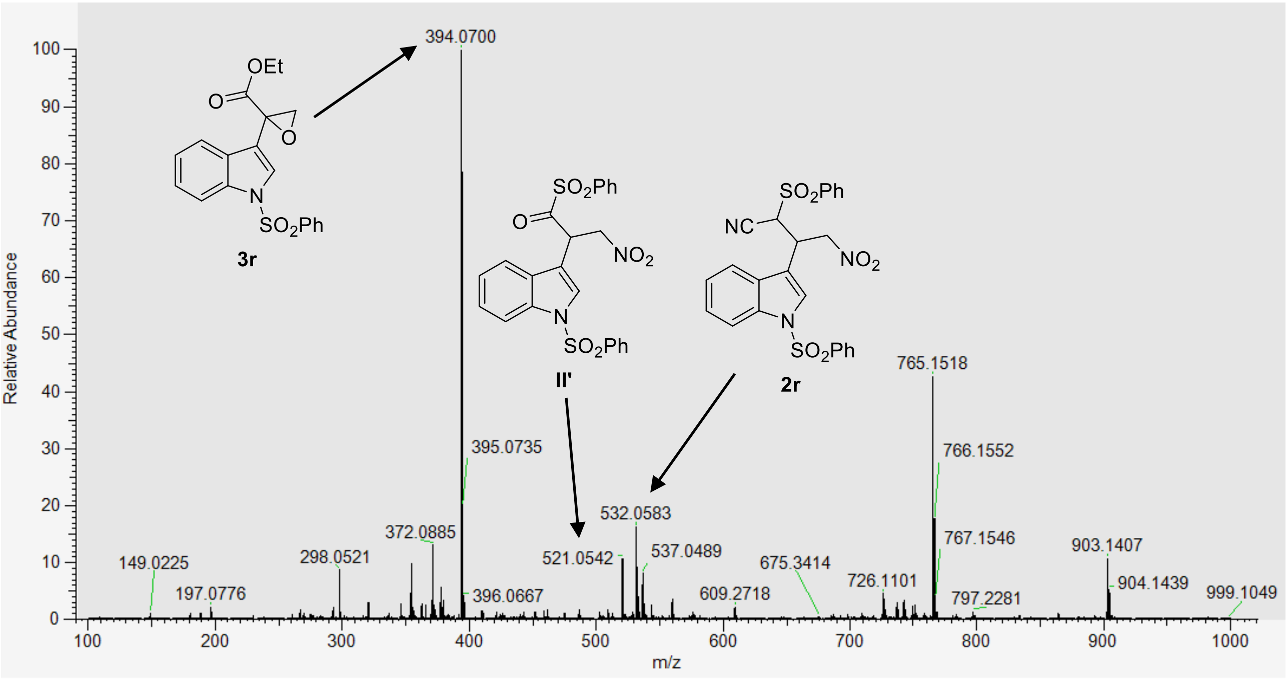

Figure S1. ESI-HRMS analysis of the reaction mixture at t = 10 min

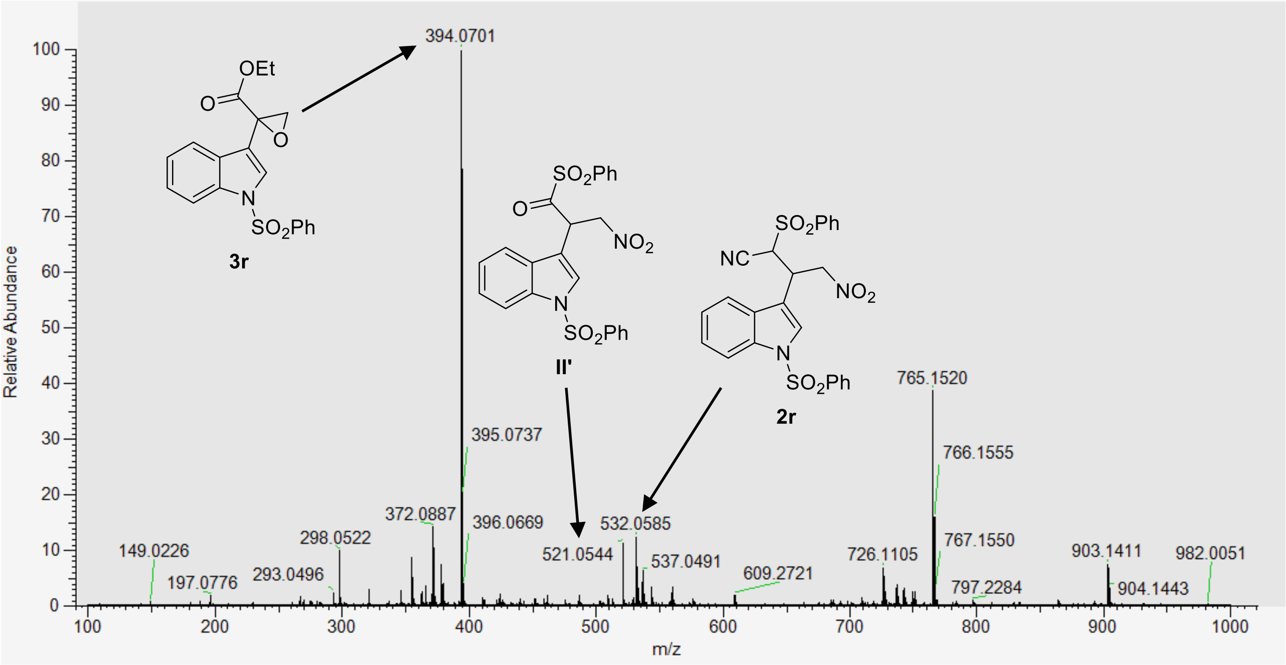

Figure S2. ESI-HRMS analysis of the reaction mixture at t = 30 min

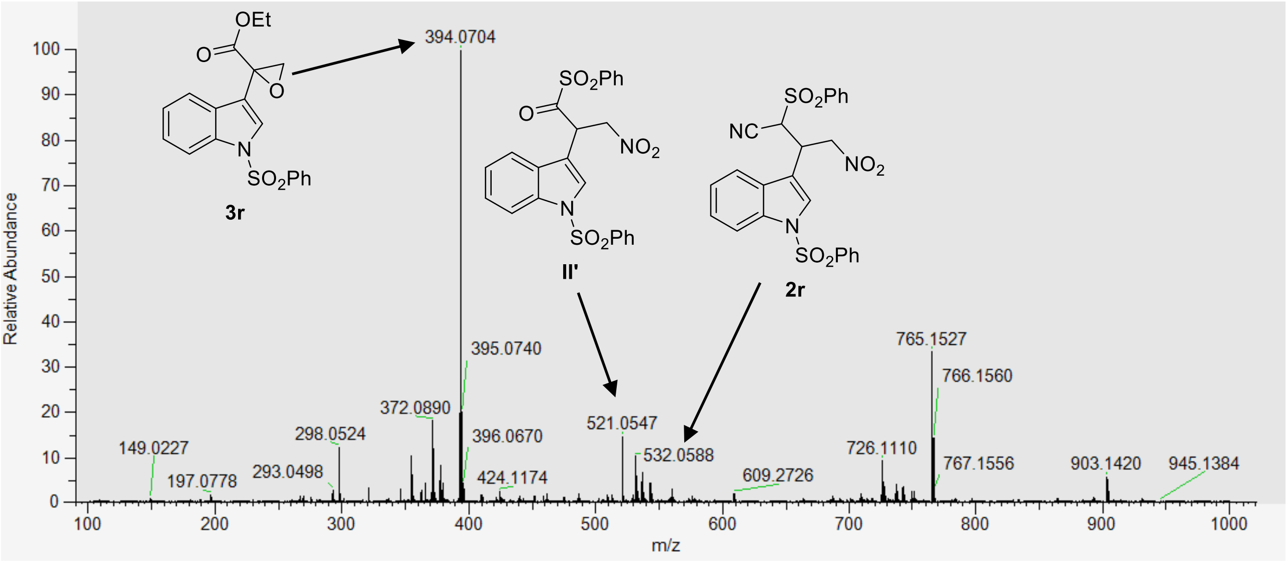

Figure S3. ESI-HRMS analysis of the reaction mixture at t = 60 min

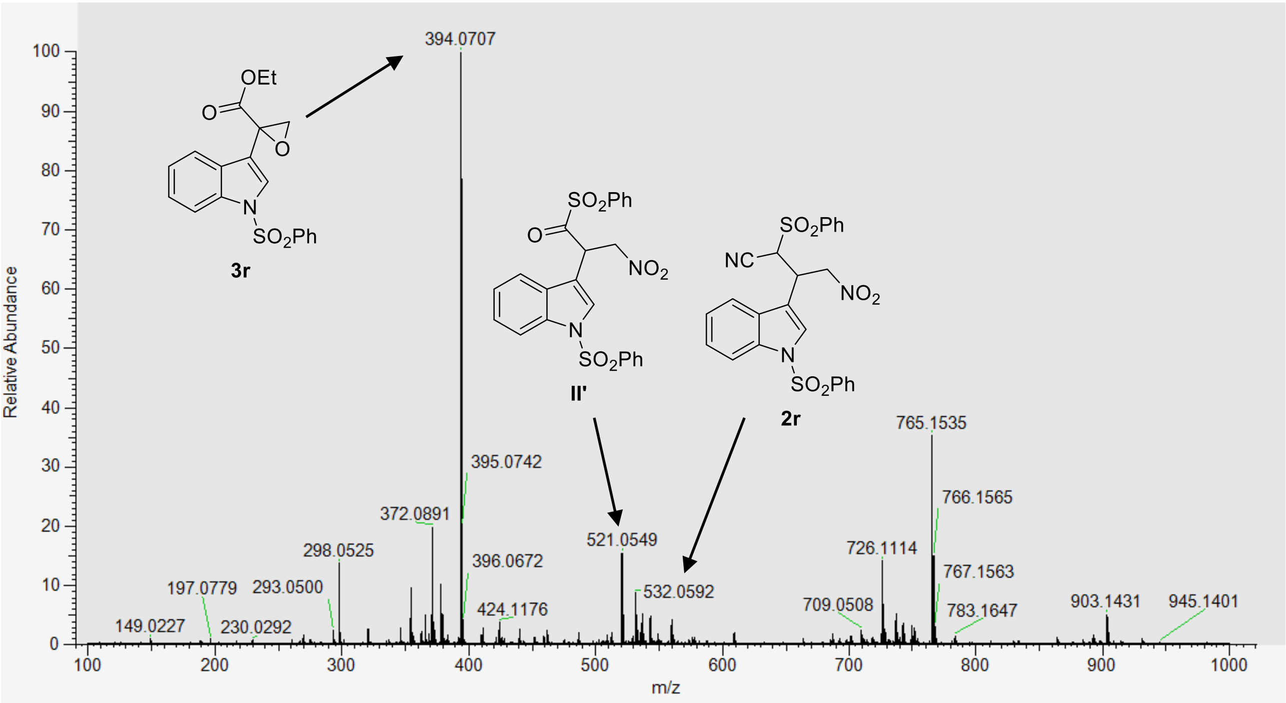

Figure S4. ESI-HRMS analysis of the reaction mixture at t = 120 min

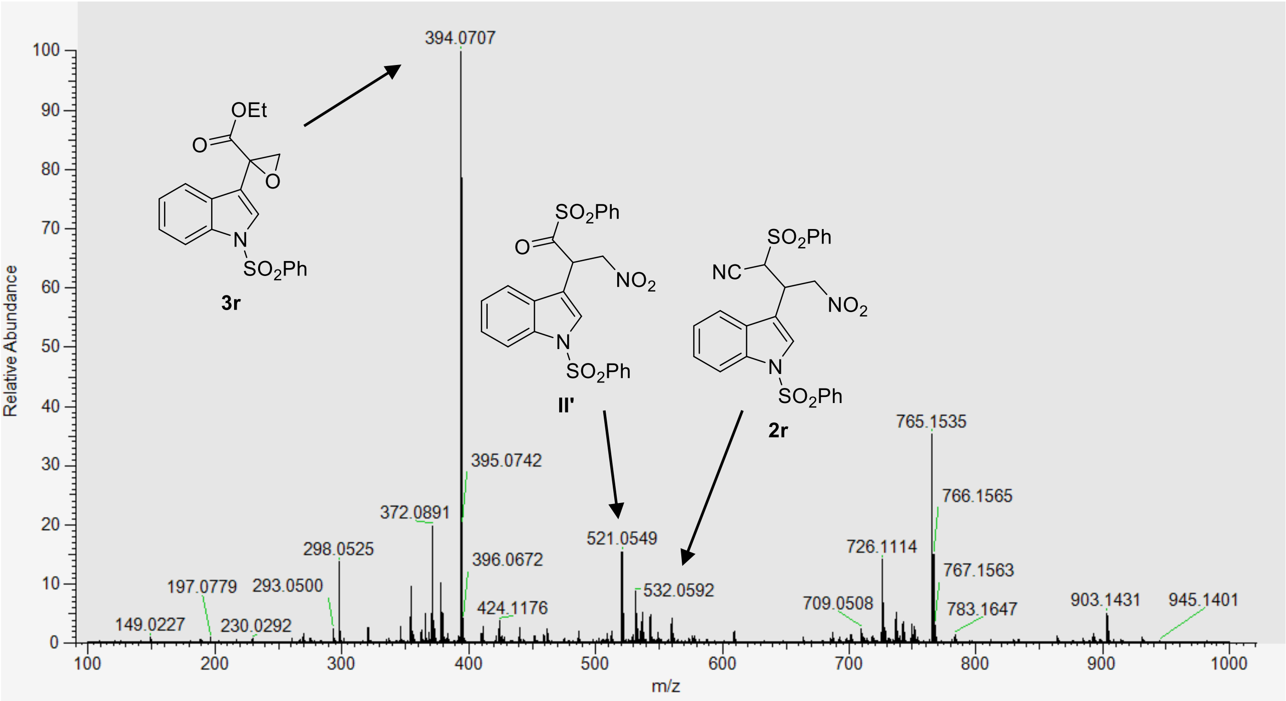

Figure S5. ESI-HRMS analysis of the reaction mixture at t = 180 min

NMR Spectra

^{1}H NMR in $CDCl_3$ (600 MHz)

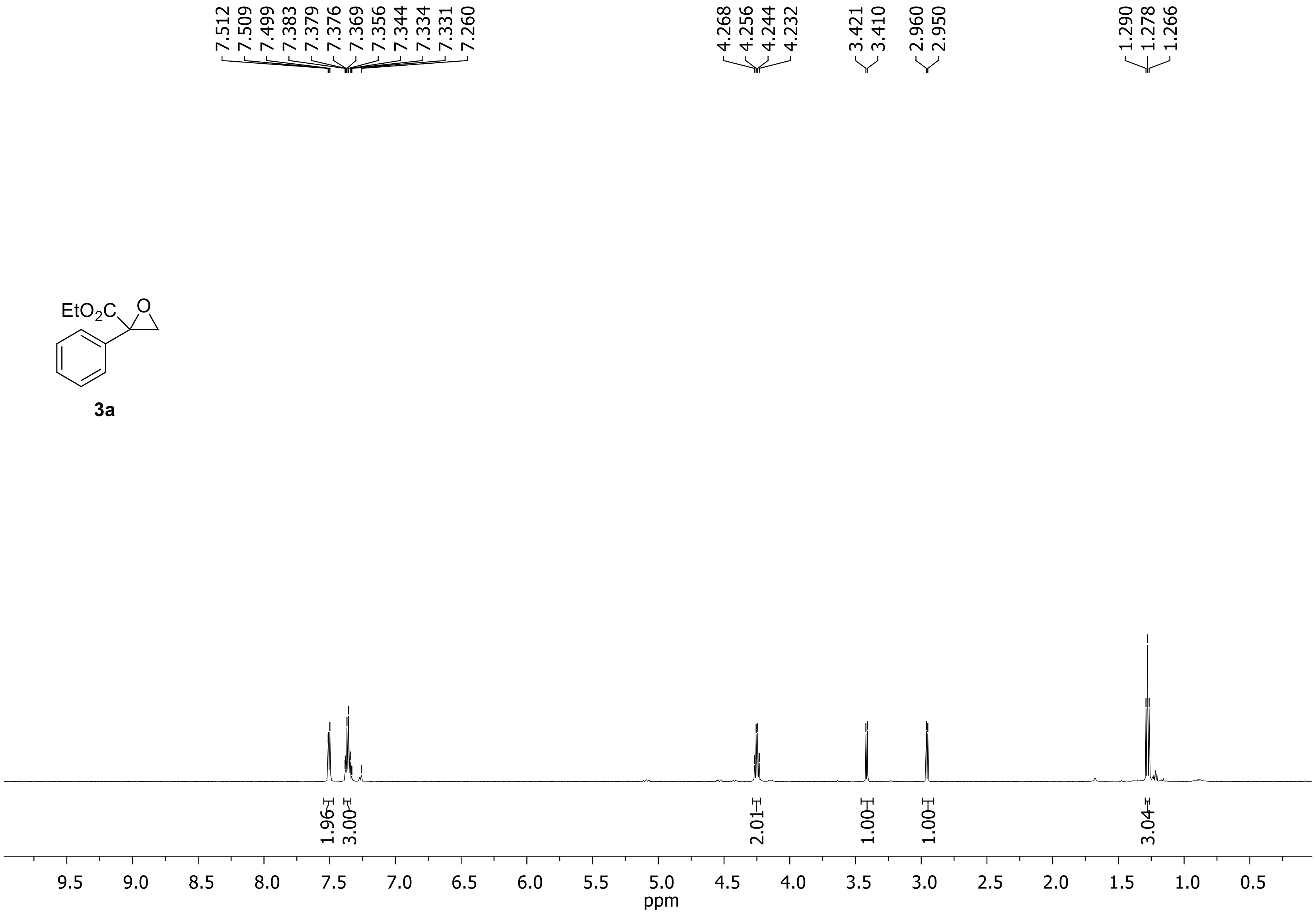

$^{13}C\{^1H\}$ NMR in $CDCl_3$ (151 MHz)

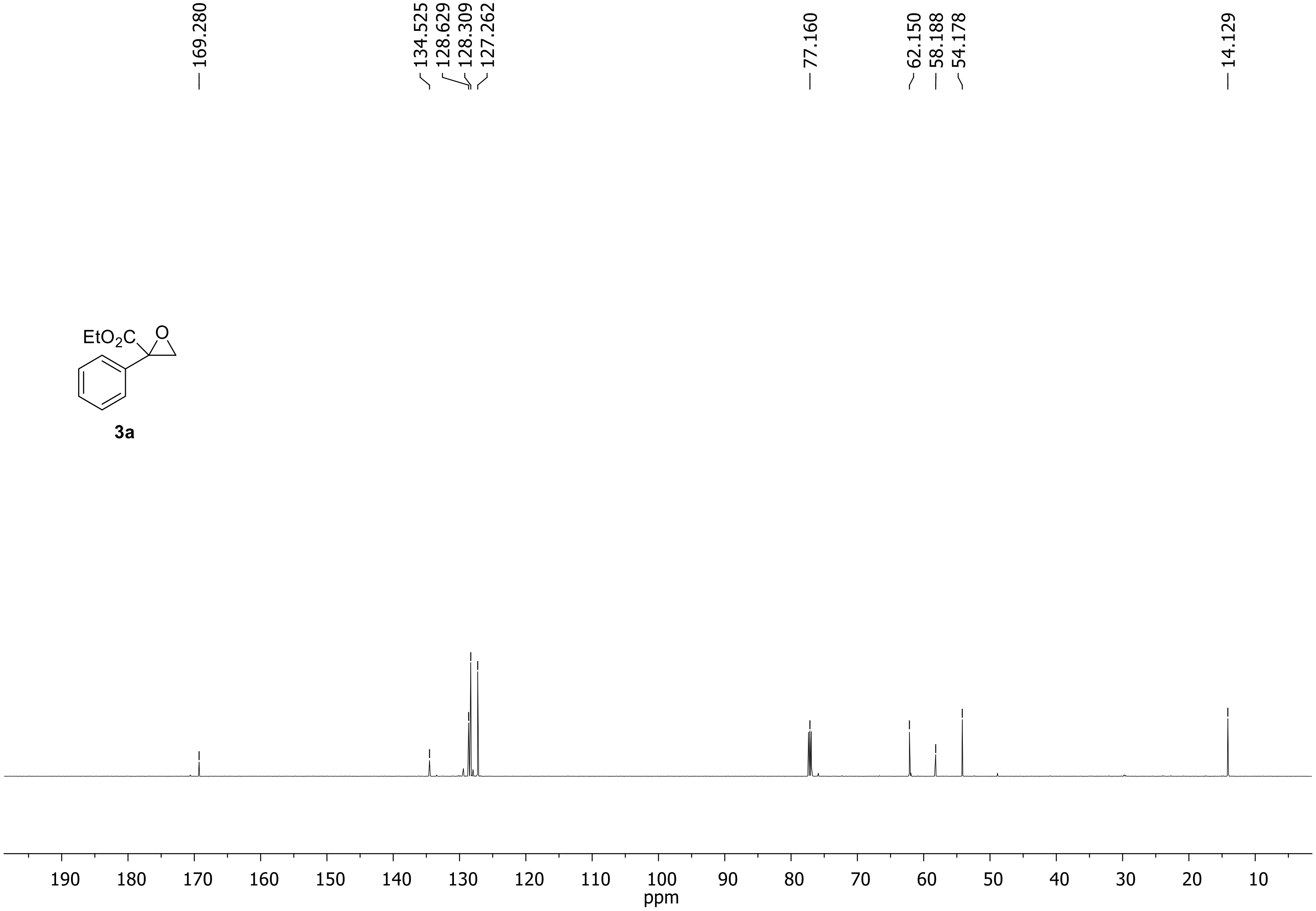

^{1}H NMR in $CDCl_3$ (600 MHz)

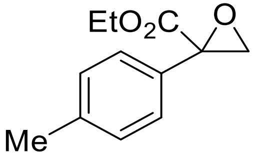

3b

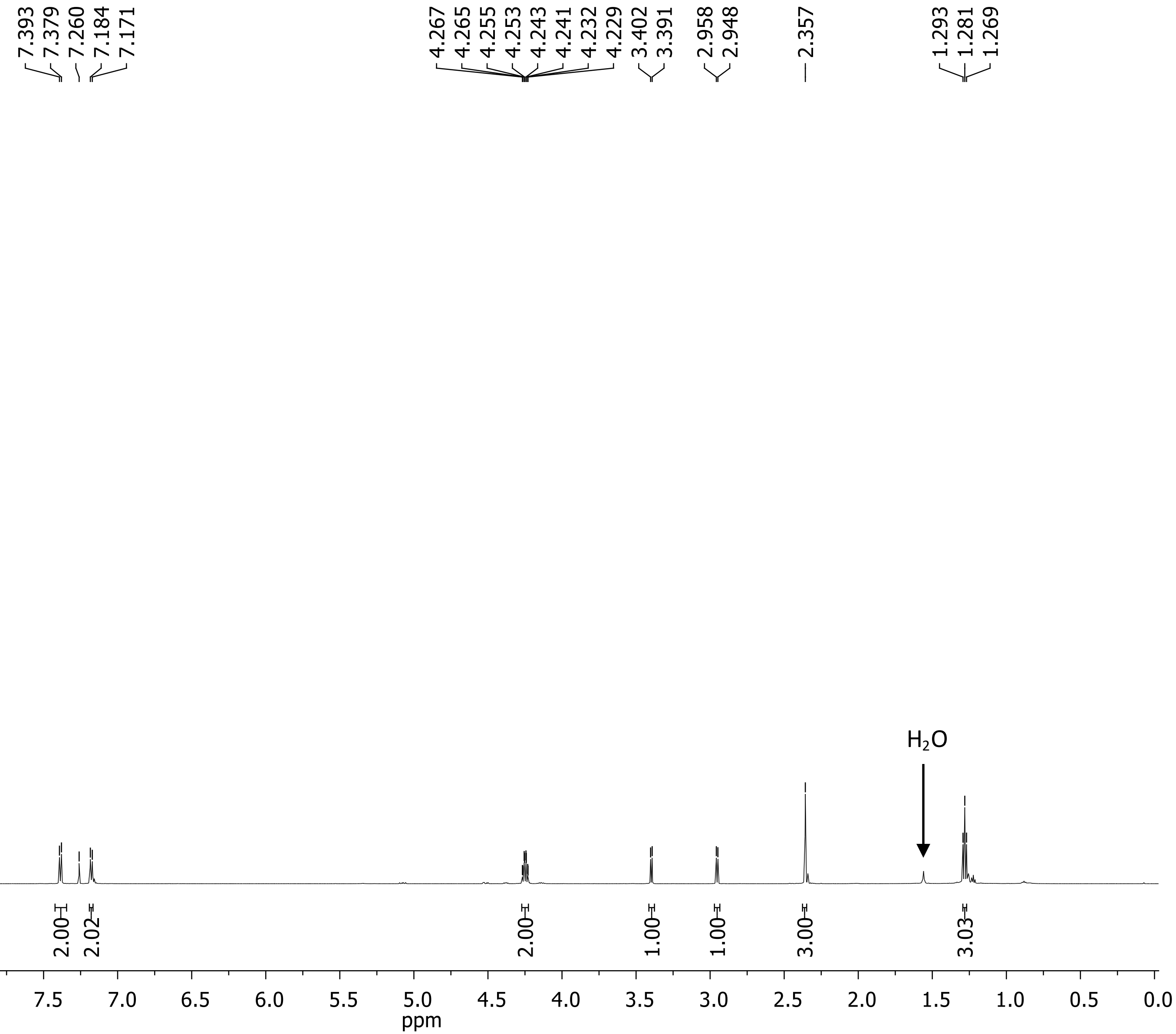

^{13}C {^{1}H} NMR in $CDCl_3$ (151 MHz)

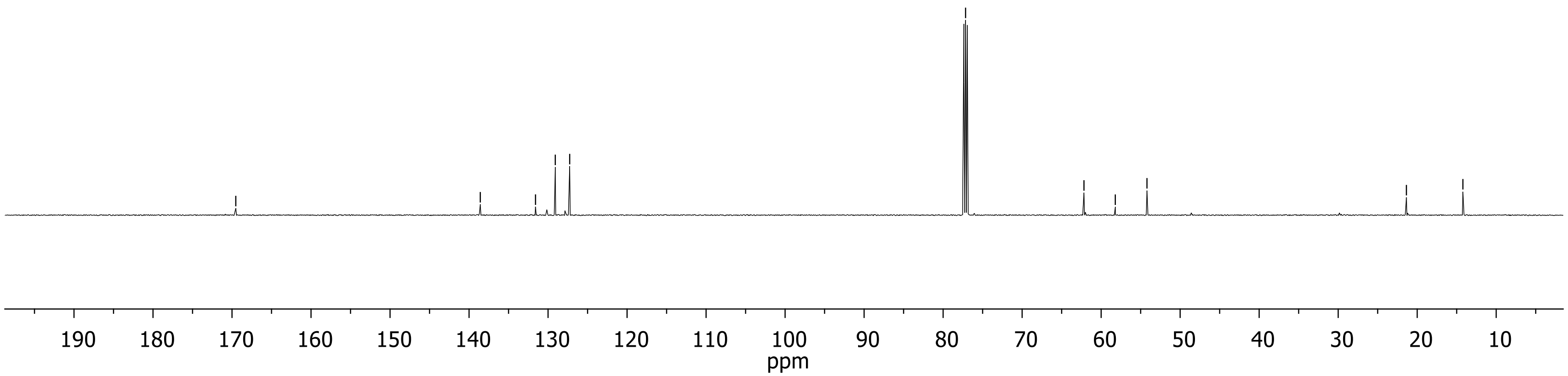

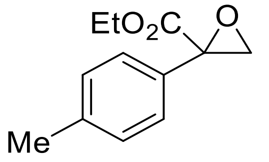

3b

^{1}H NMR in $CDCl_3$ (600 MHz)

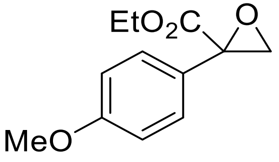

3c

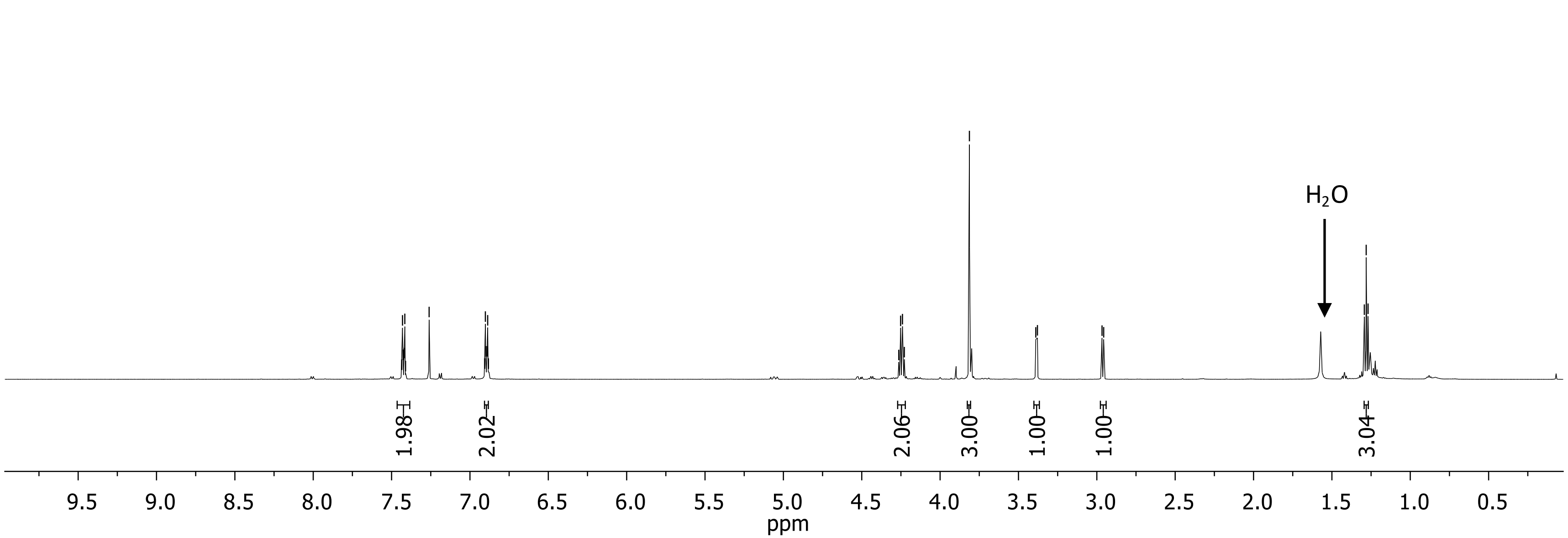

$^{13}C\ \{^{1}H\}$ NMR in $CDCl_3$ (151 MHz)

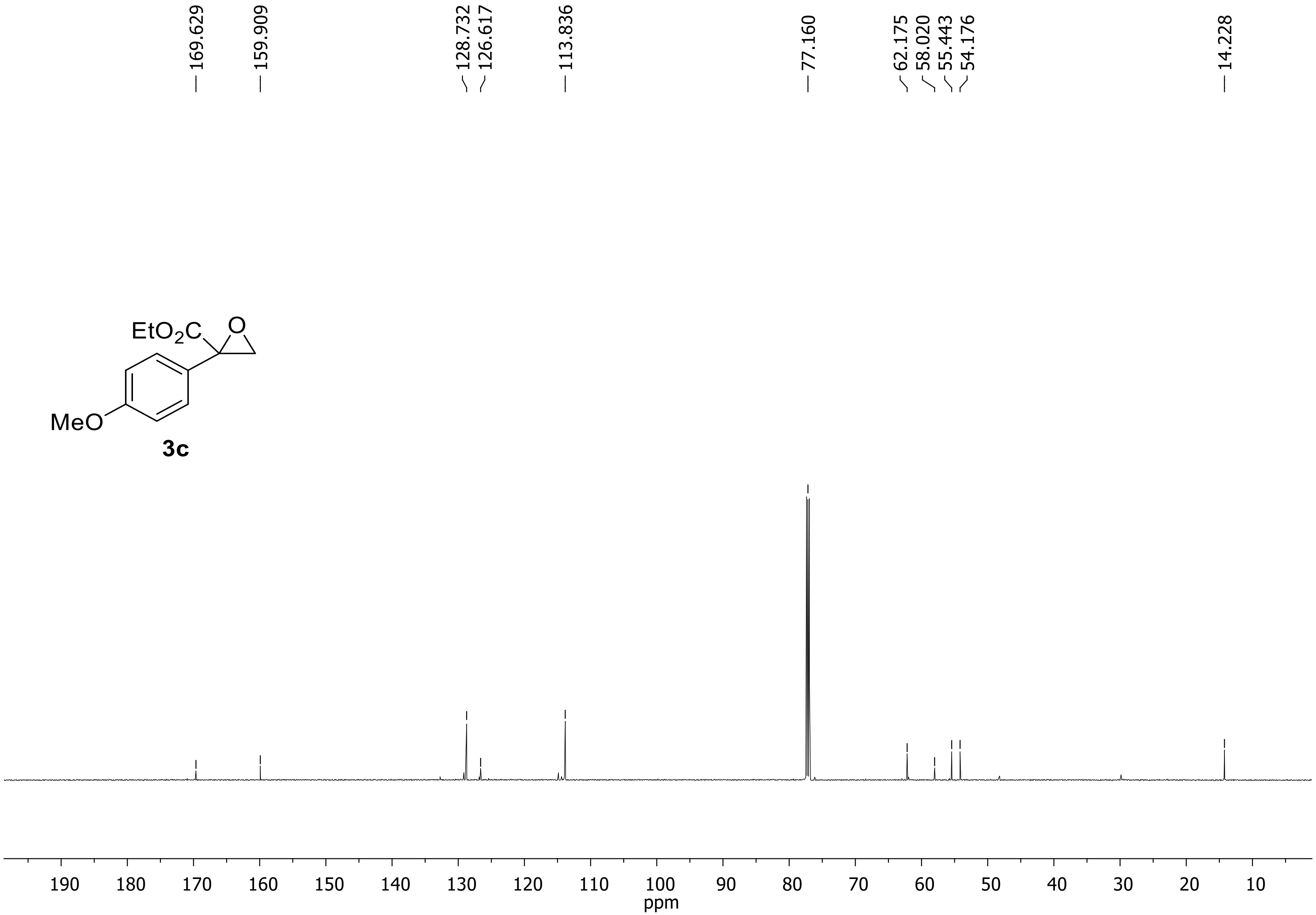

^{1}H NMR in $CDCl_3$ (600 MHz)

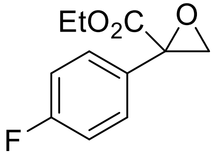

3d

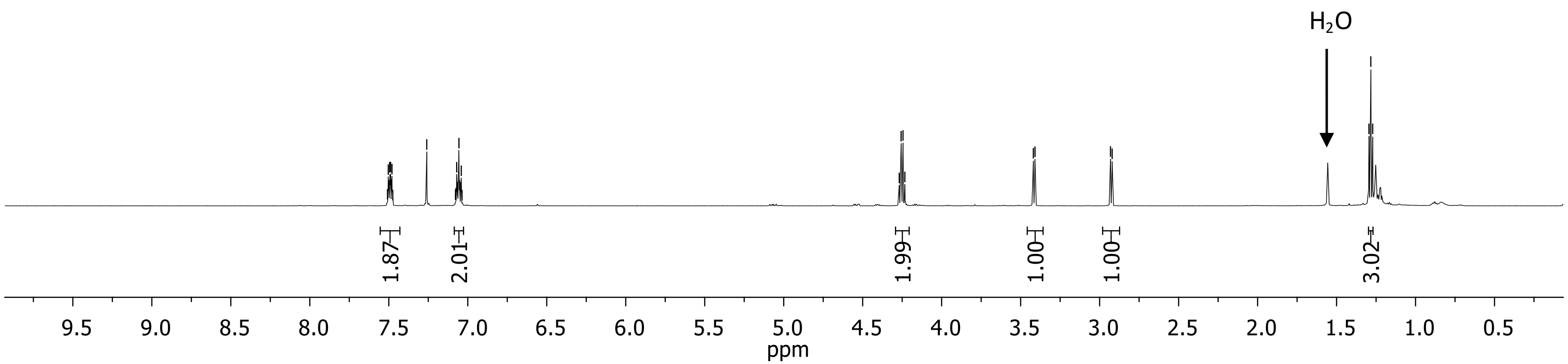

^{13}C {^{1}H} NMR in $CDCl_3$ (151 MHz)

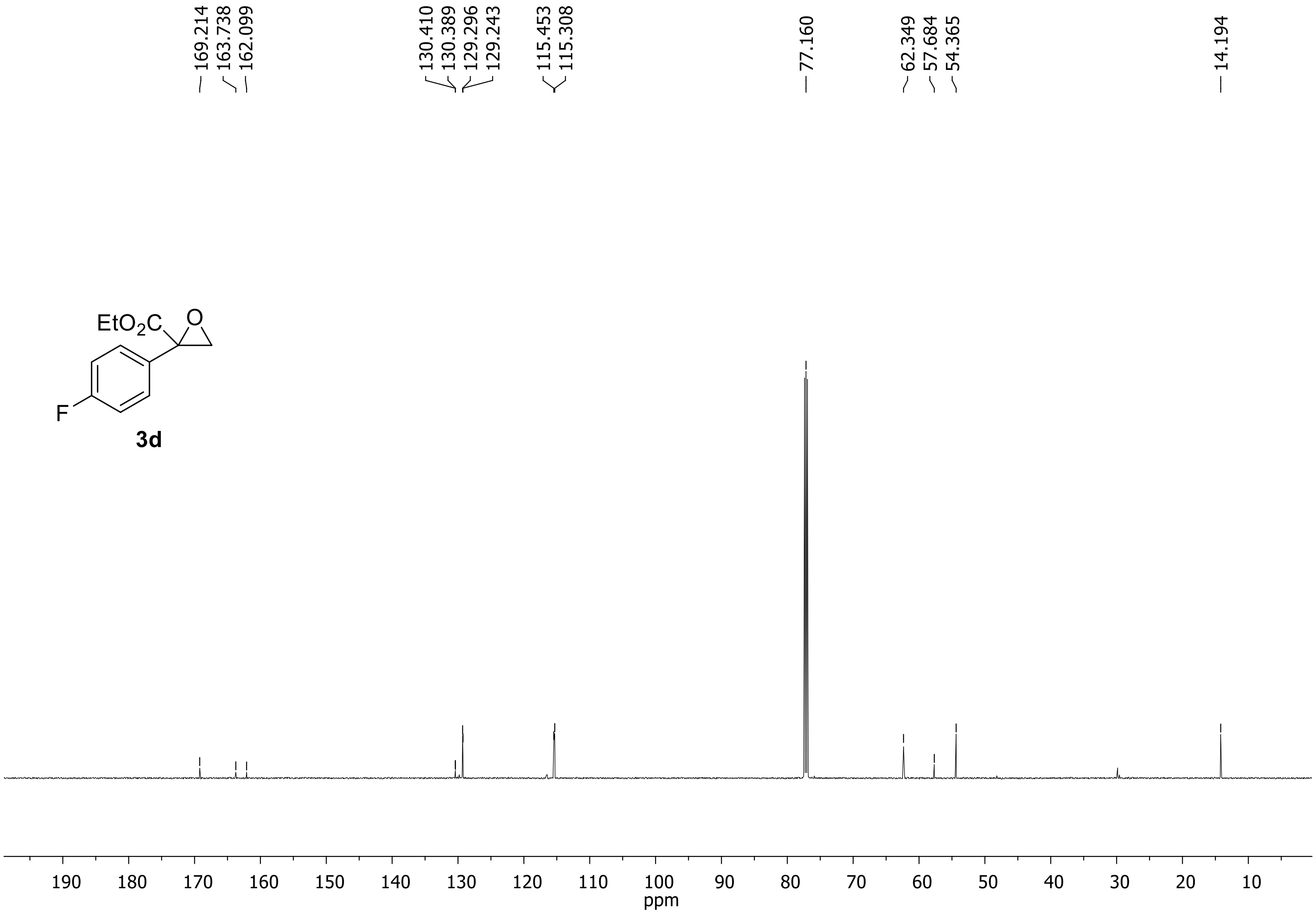

3d

^{19}F {^{1}H} NMR in $CDCl_3$ (565 MHz)

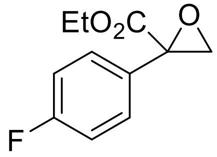

3d

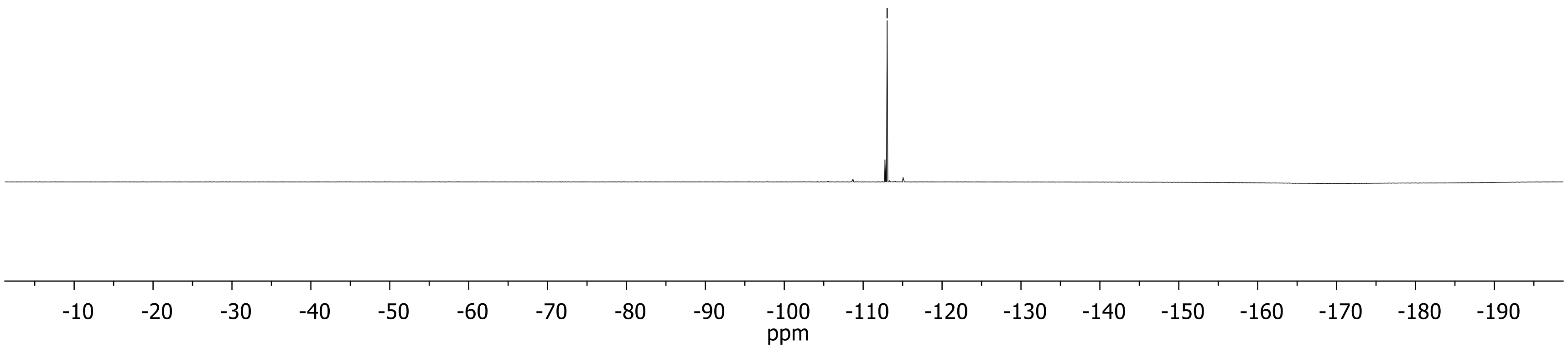

^1H NMR in $CDCl_3$ (400 MHz)

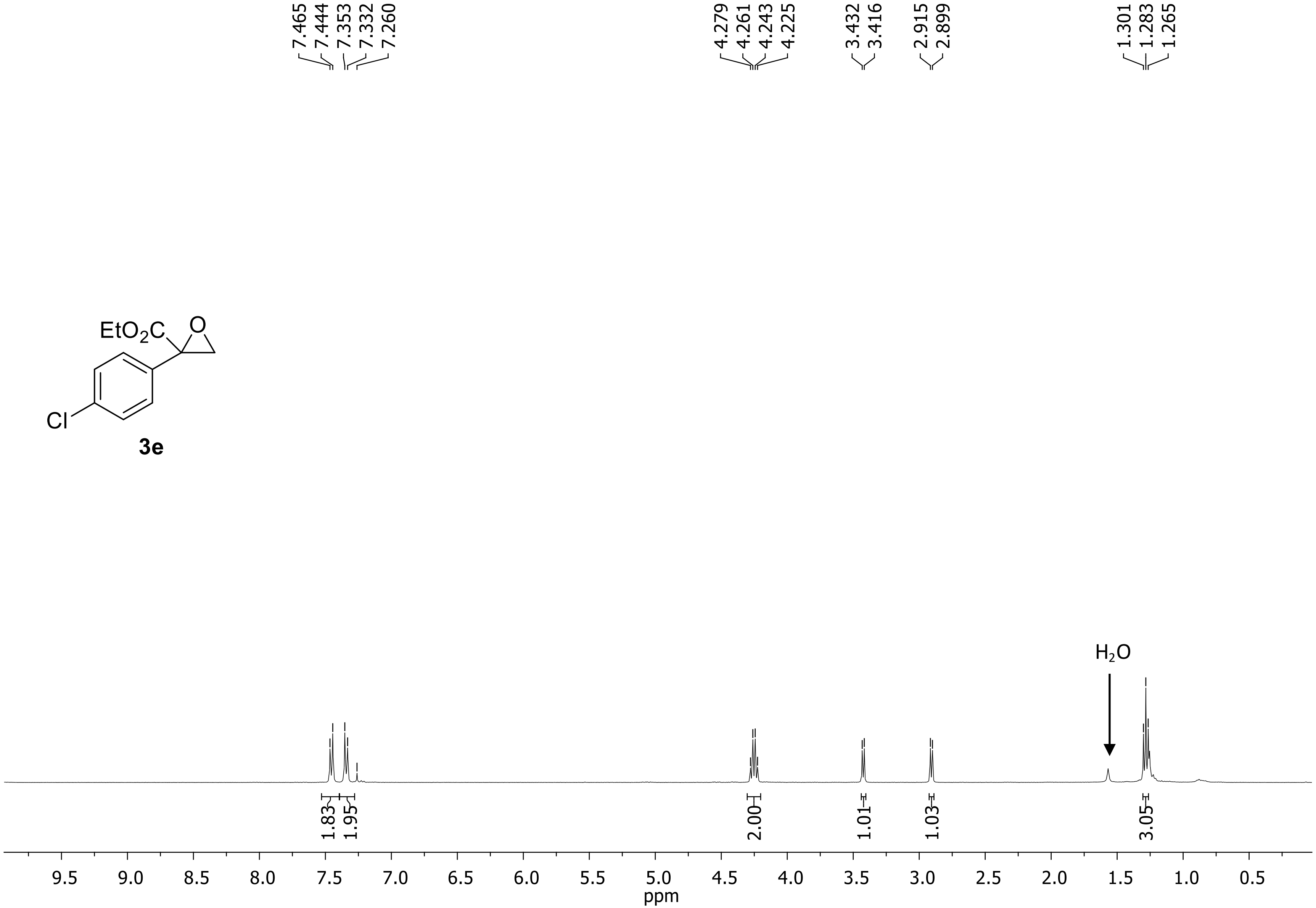

3e

^{13}C {^1H} NMR in $CDCl_3$ (101 MHz)

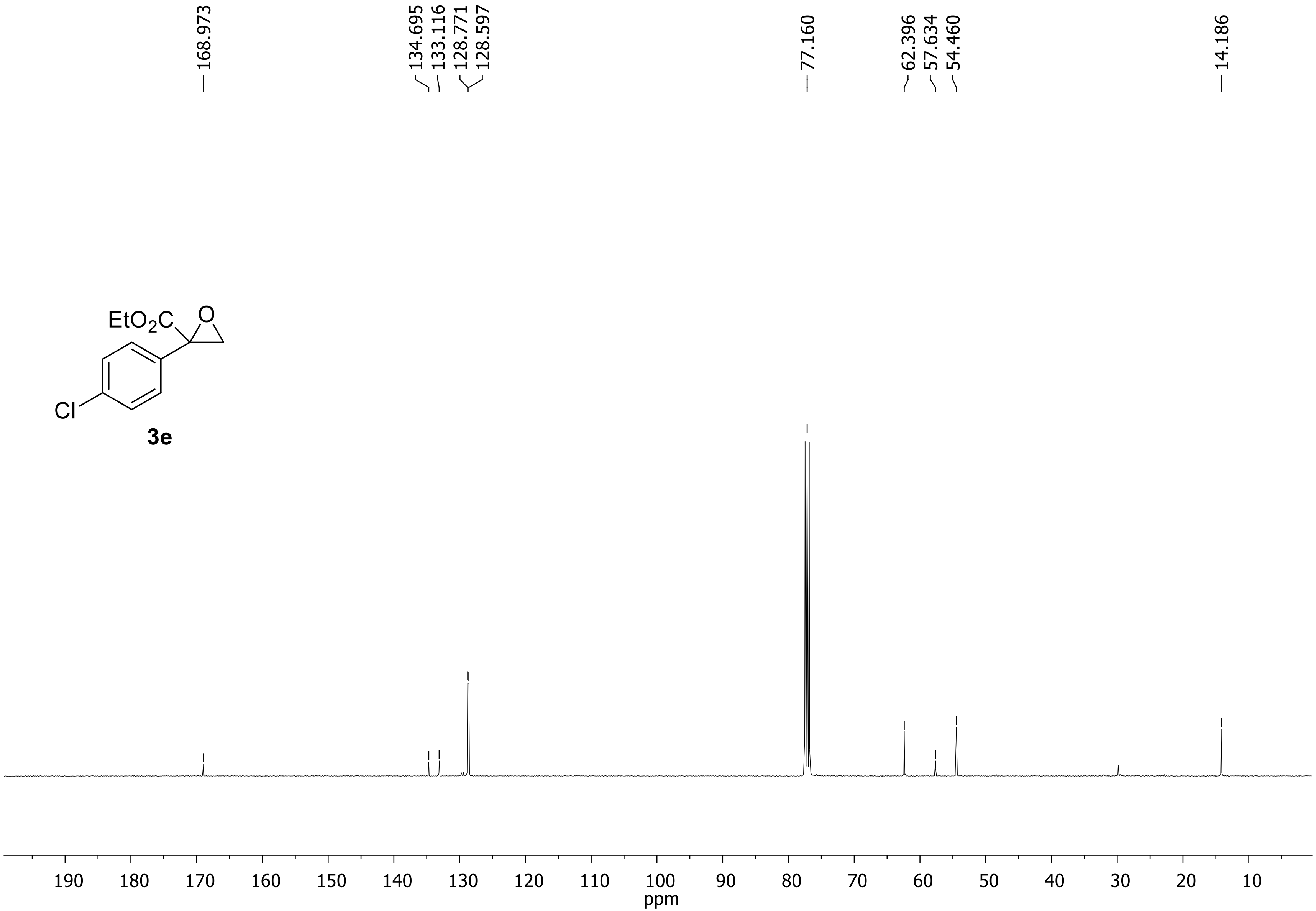

^{1}H NMR in $CDCl_3$ (300 MHz)

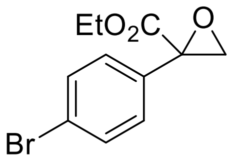

3f

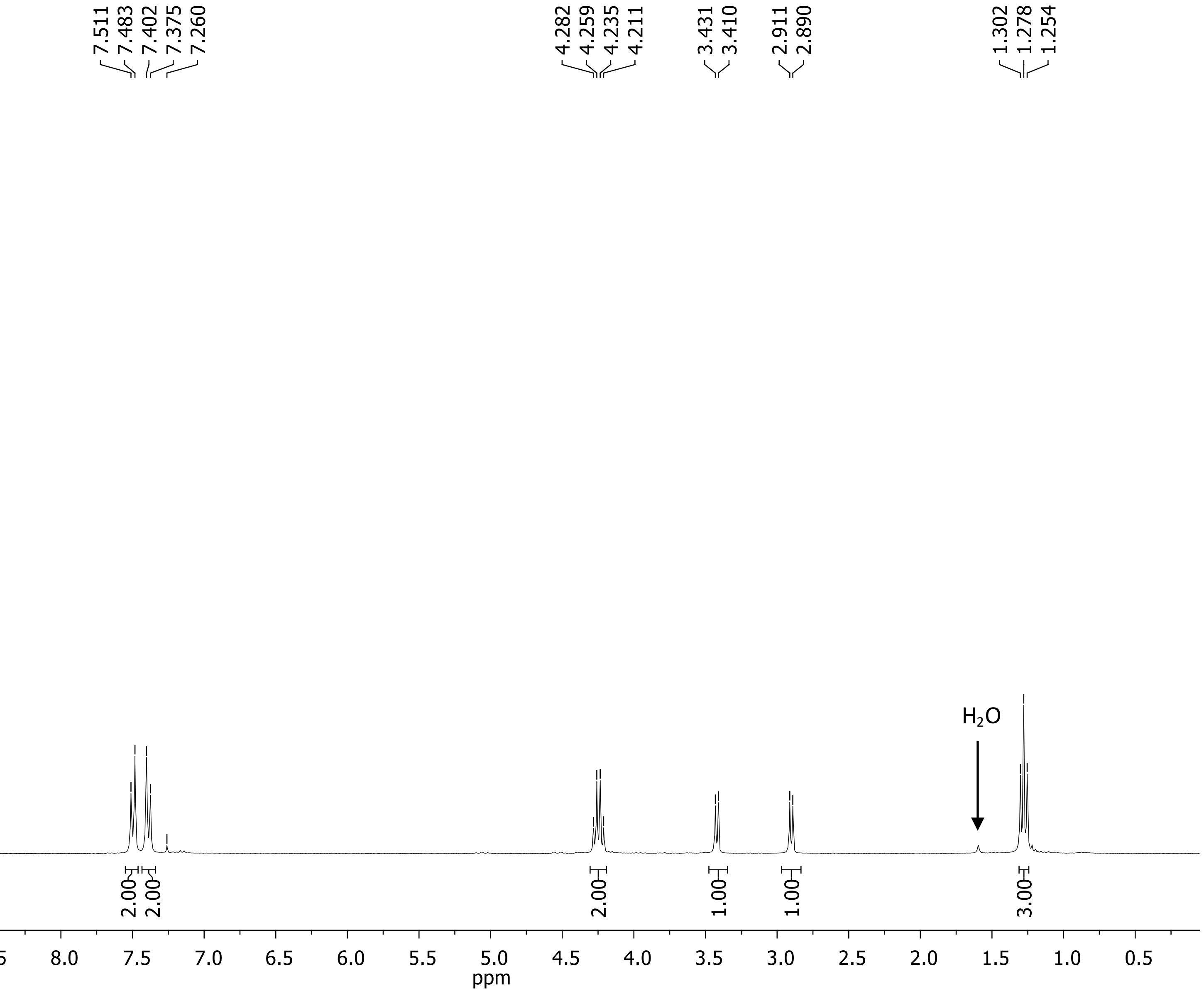

^{13}C {^{1}H} NMR in $CDCl_3$ (62.5 MHz)

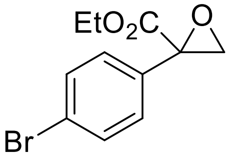

3f

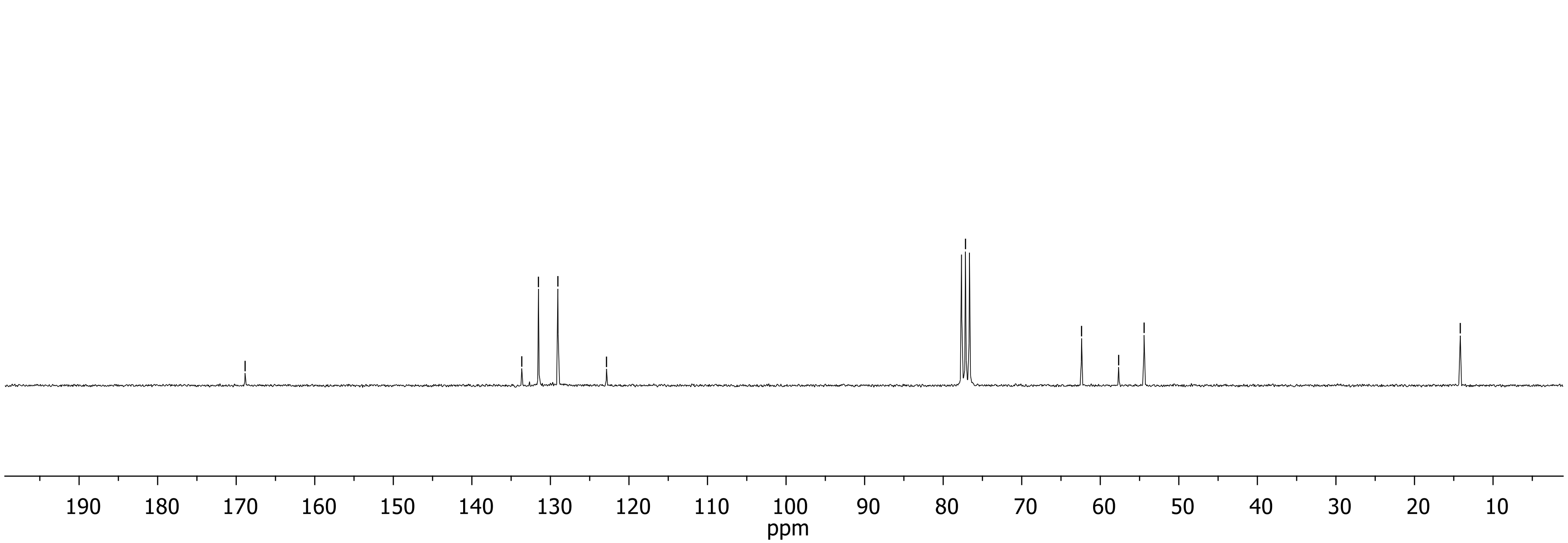

^{1}H NMR in $CDCl_3$ (400 MHz)

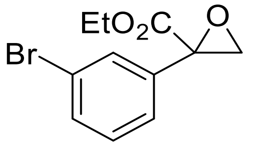

3g

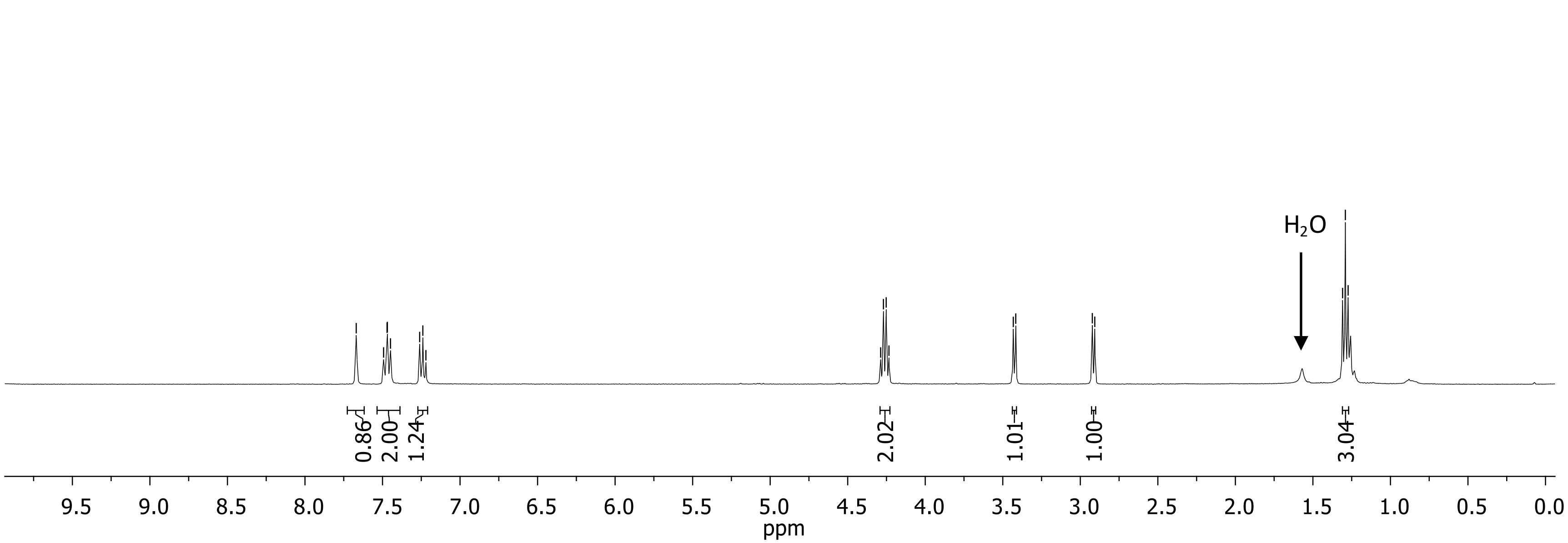

^{13}C {^{1}H} NMR in $CDCl_3$ (75 MHz)

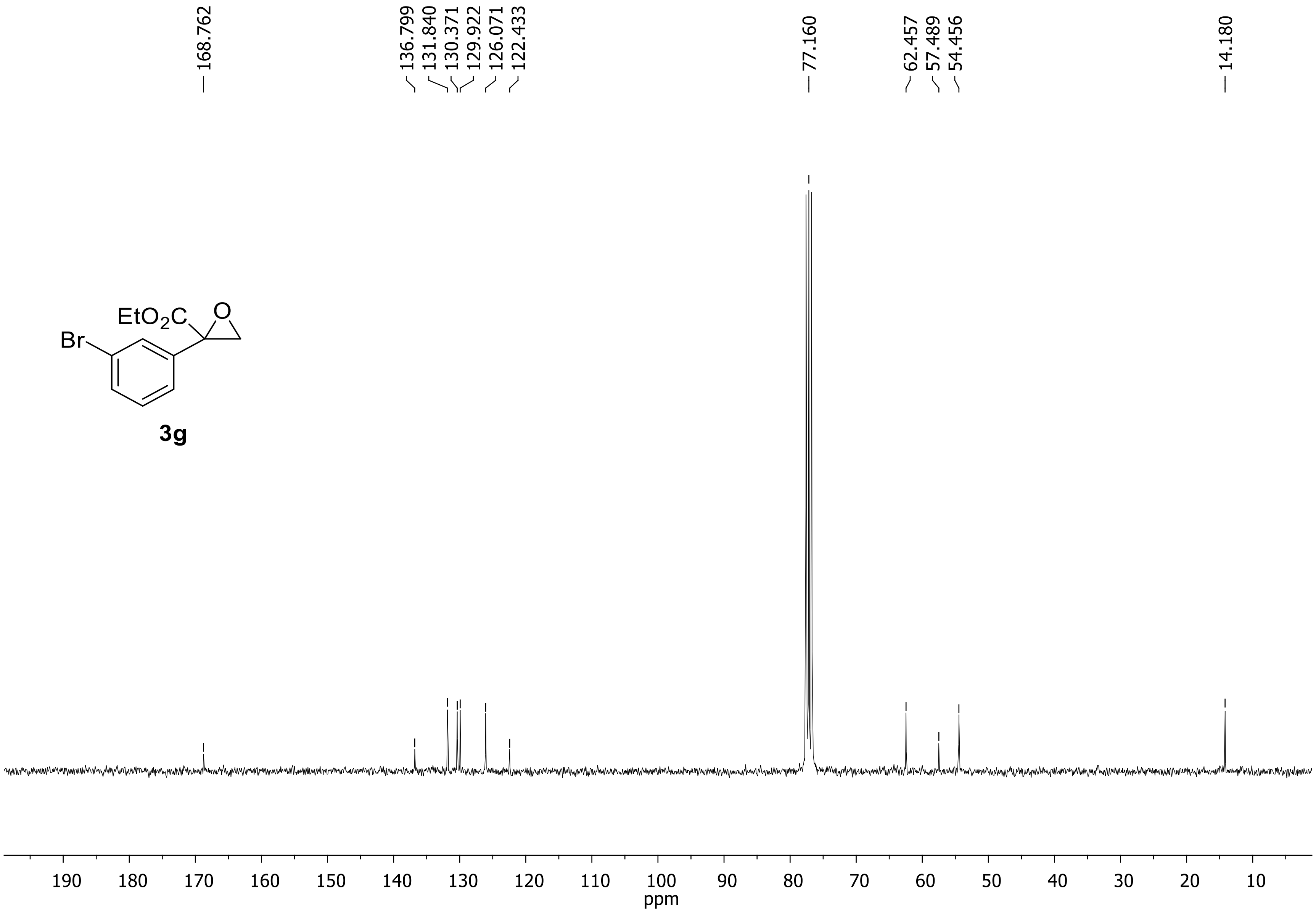

^{1}H NMR in $CDCl_3$ (400 MHz)

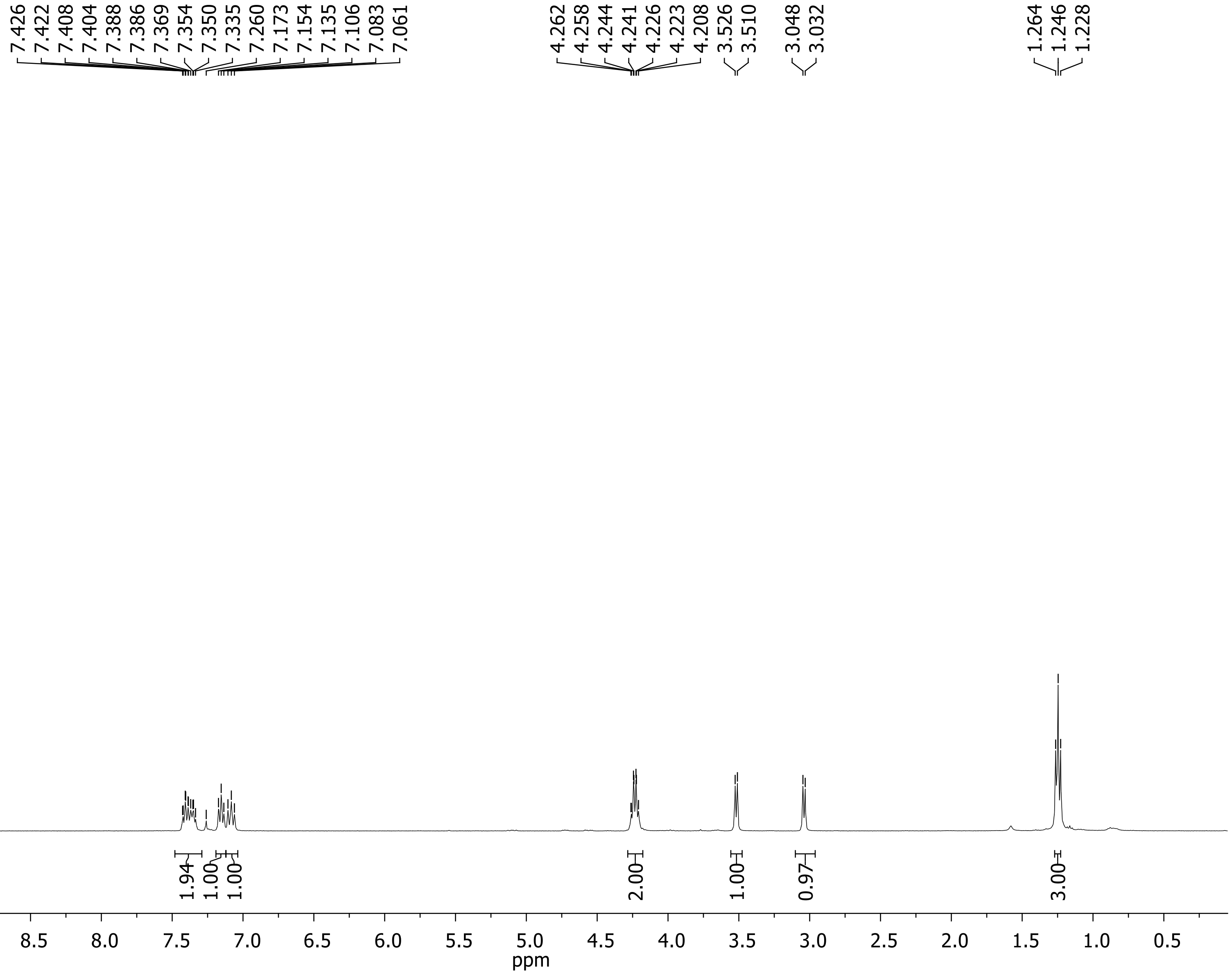

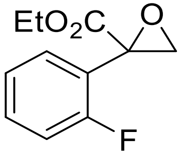

3h

^{13}C {^{1}H} NMR in $CDCl_3$ (62.5 MHz)

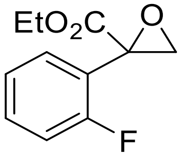

3h

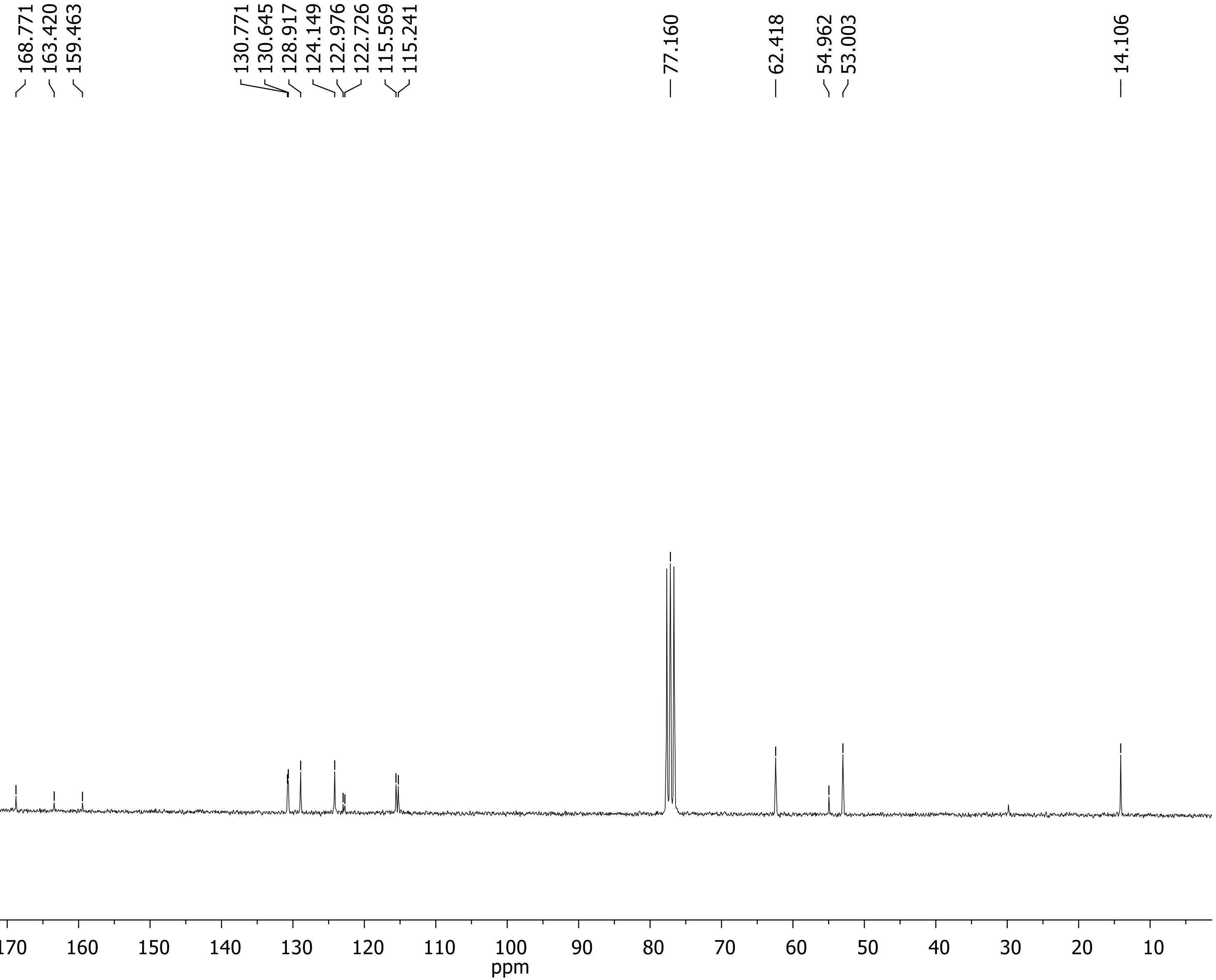

^{19}F {^{1}H} NMR in $CDCl_3$ (376 MHz)

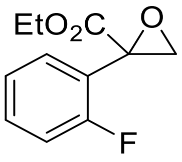

3h

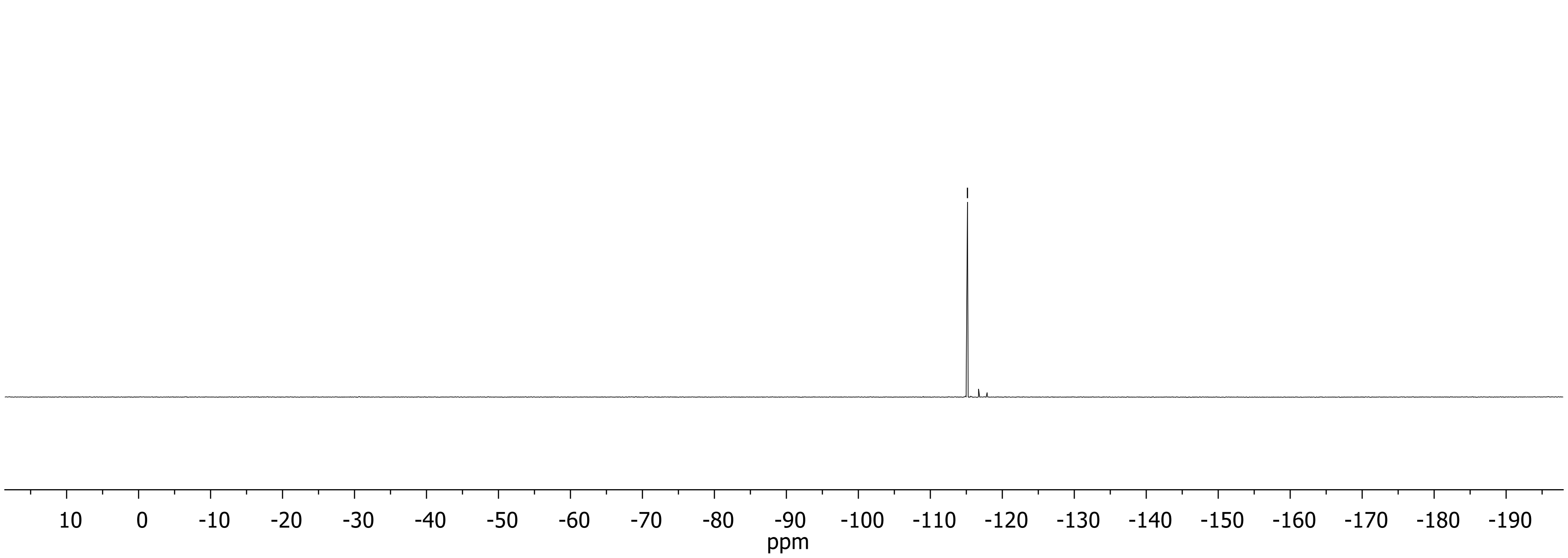

^1H NMR in $CDCl_3$ (400 MHz)

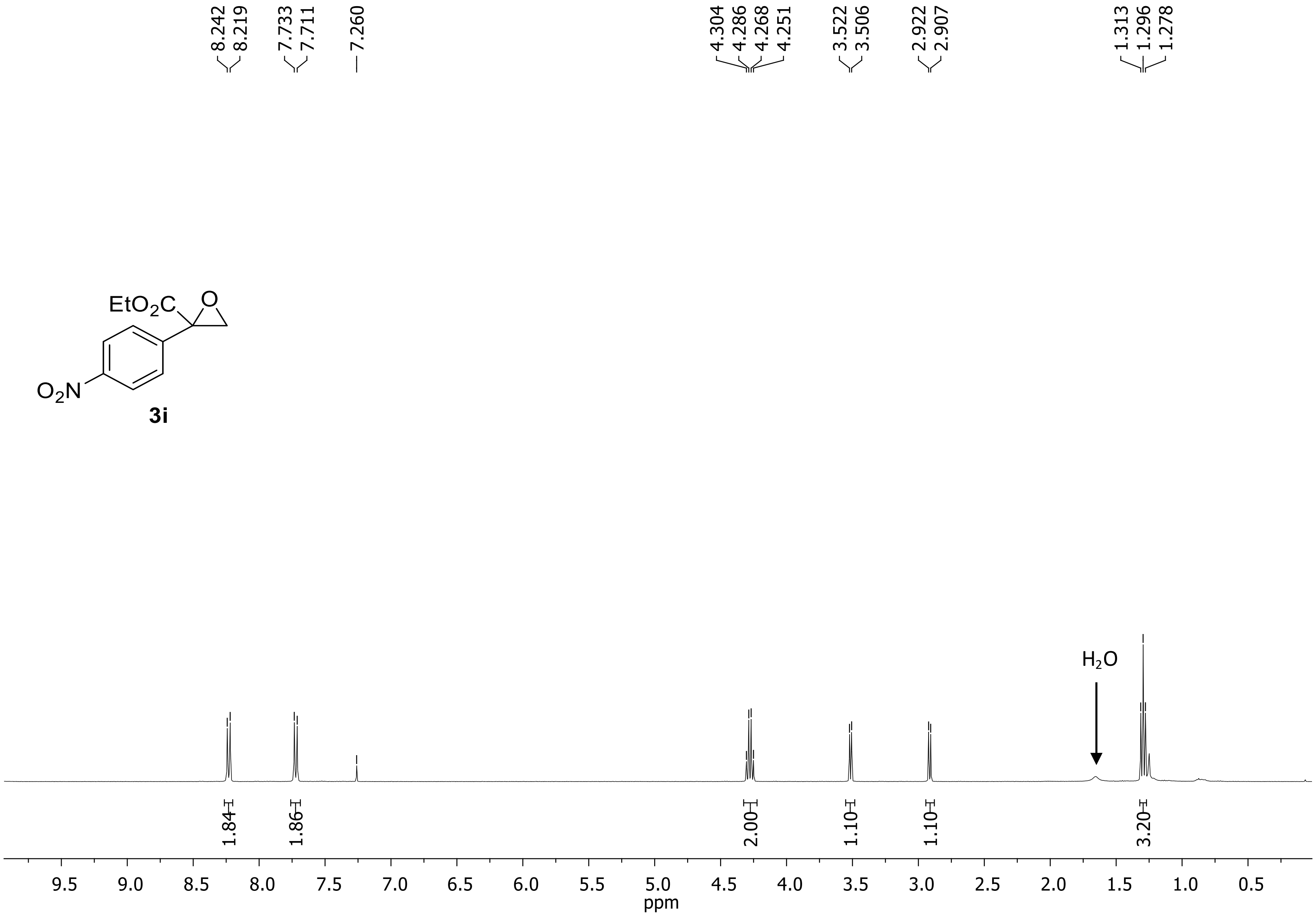

$^{13}C\ \{^1H\}$ NMR in $CDCl_3$ (62.5 MHz)

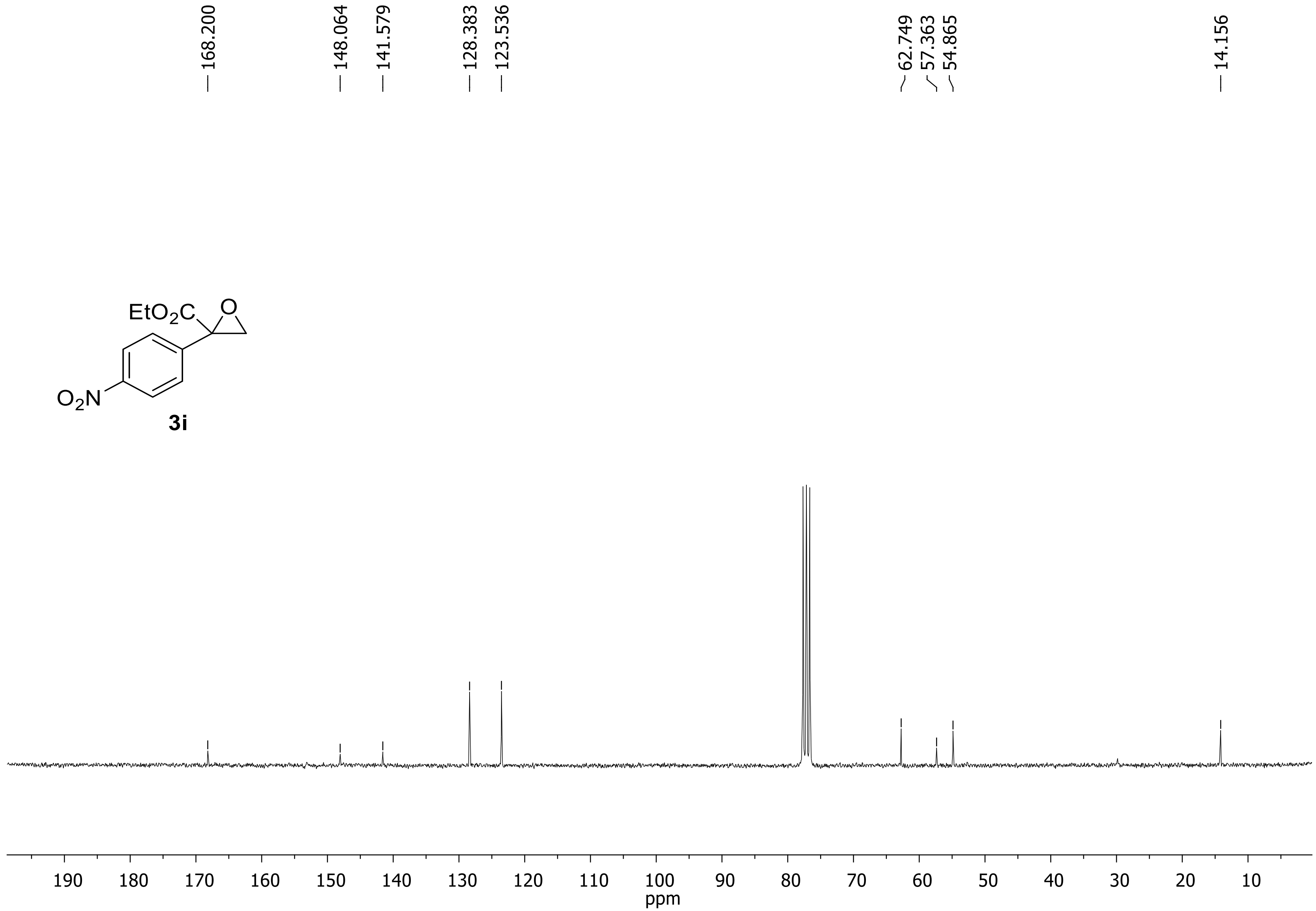

^{1}H NMR in $CDCl_3$ (600 MHz)

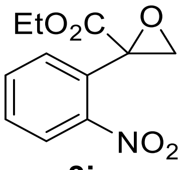

3j

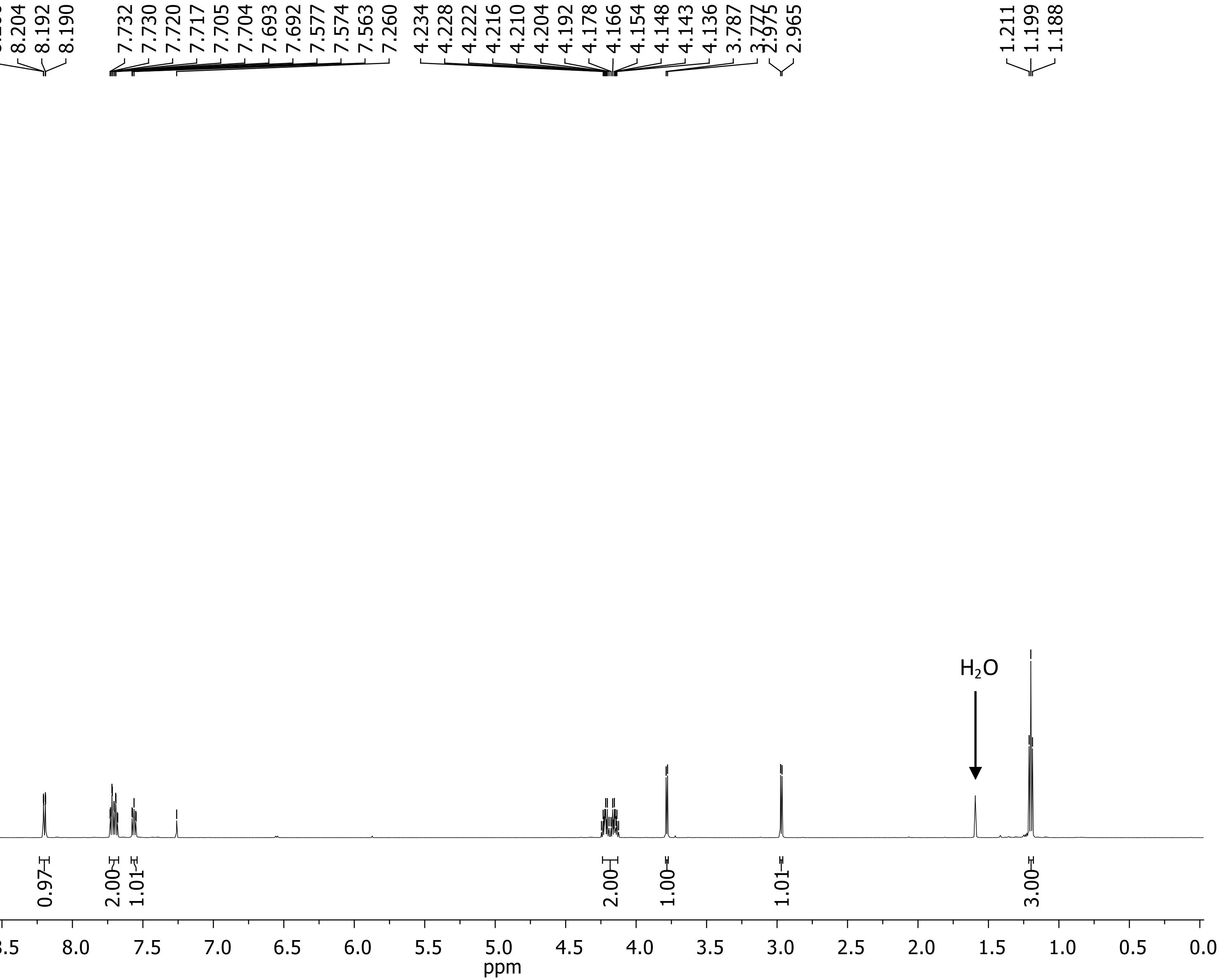

$^{13}C\ \{^{1}H\}$ NMR in $CDCl_3$ (151 MHz)

167.844
148.082
134.202
131.474
129.693
129.113
124.779
77.160
62.618
57.818
54.630
13.982

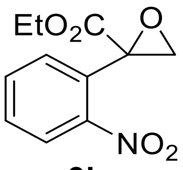

3j

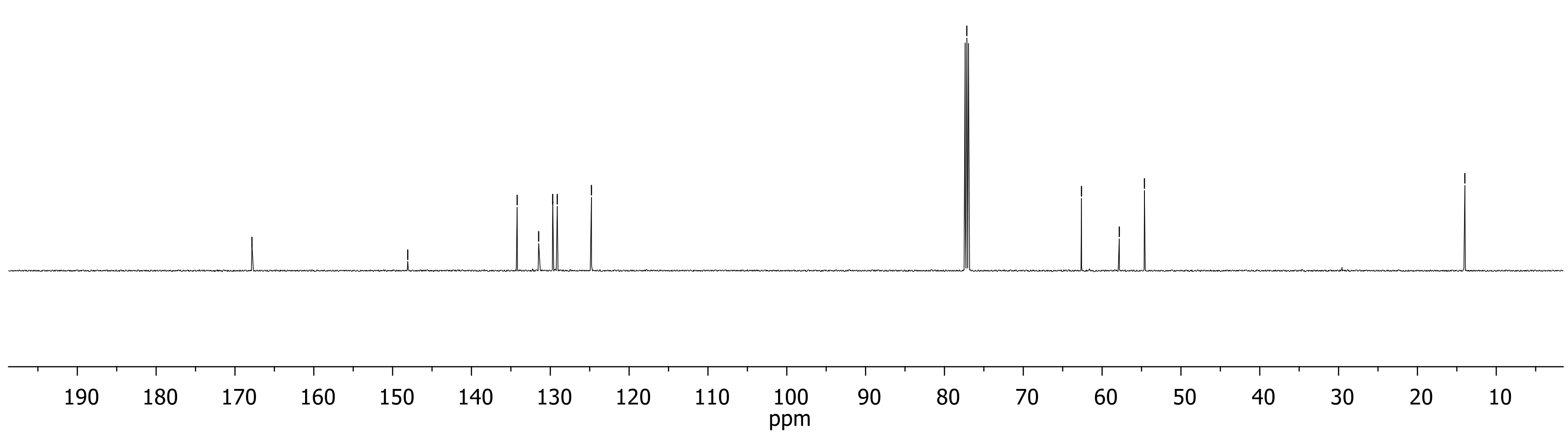

^{1}H NMR in $CDCl_3$ (600 MHz)

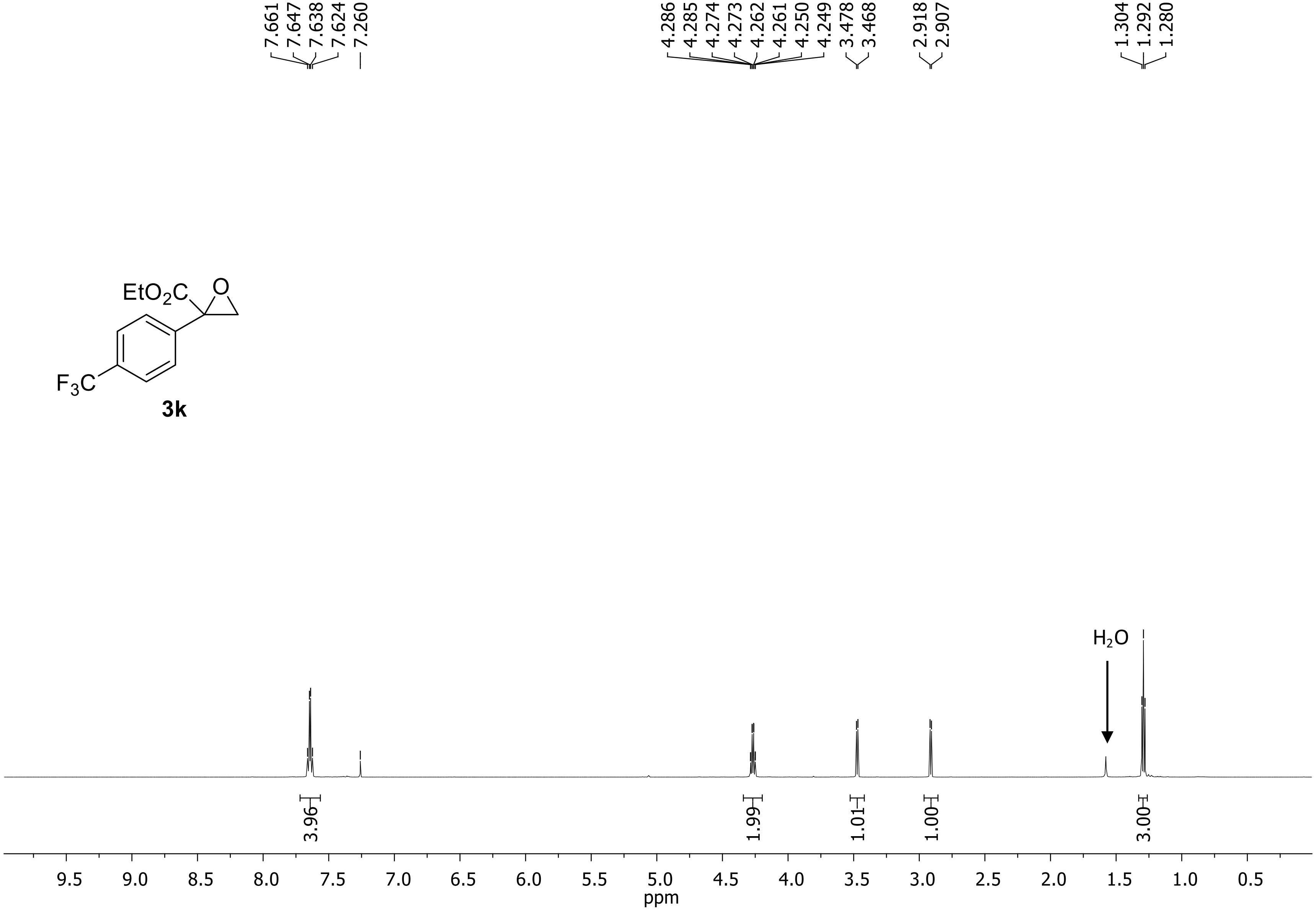

^{13}C {^{1}H} NMR in $CDCl_3$ (151 MHz)

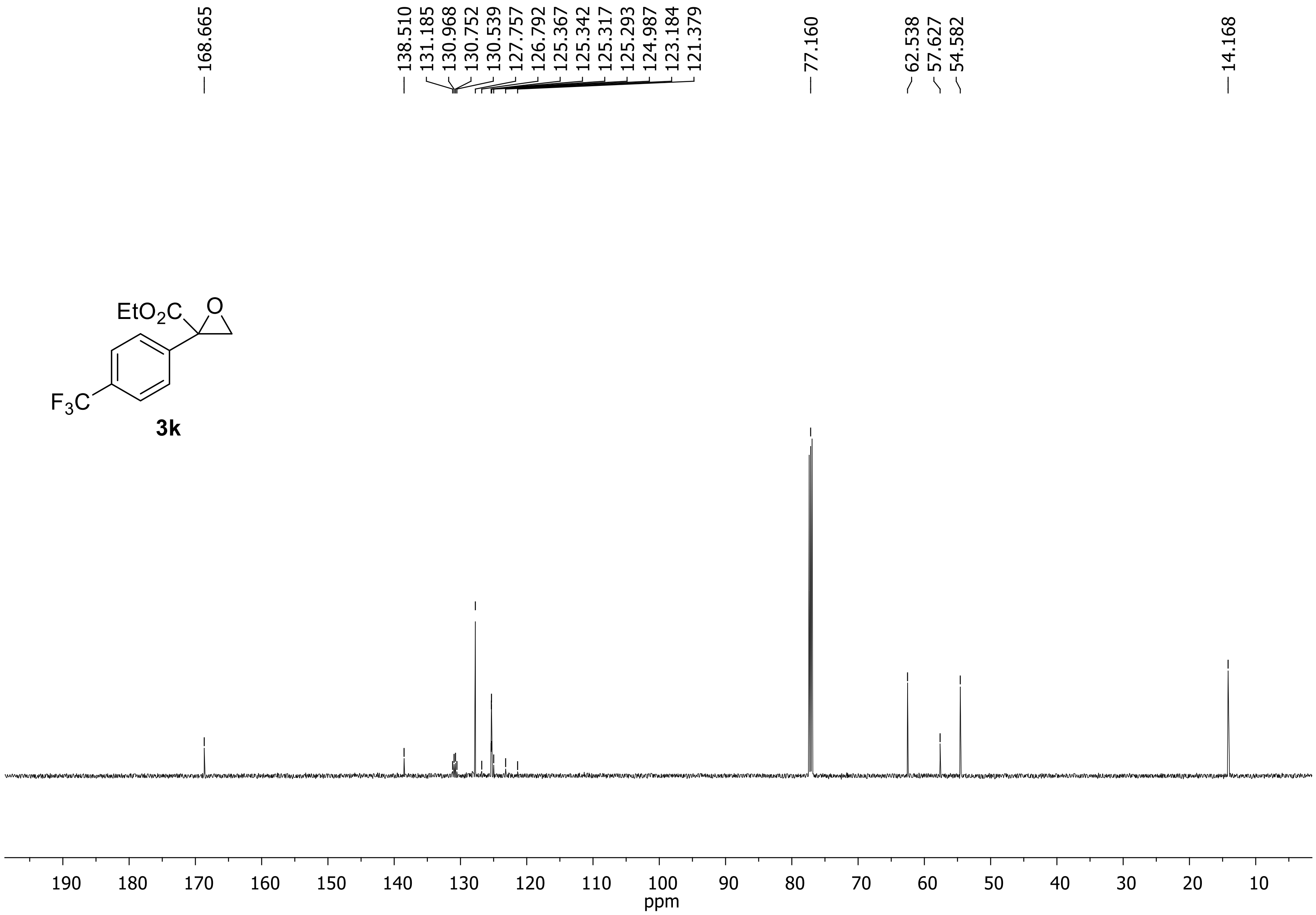

$^{19}F\{^{1}H\}$ NMR in $CDCl_3$ (566 MHz)

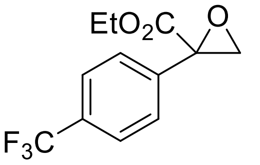

3k

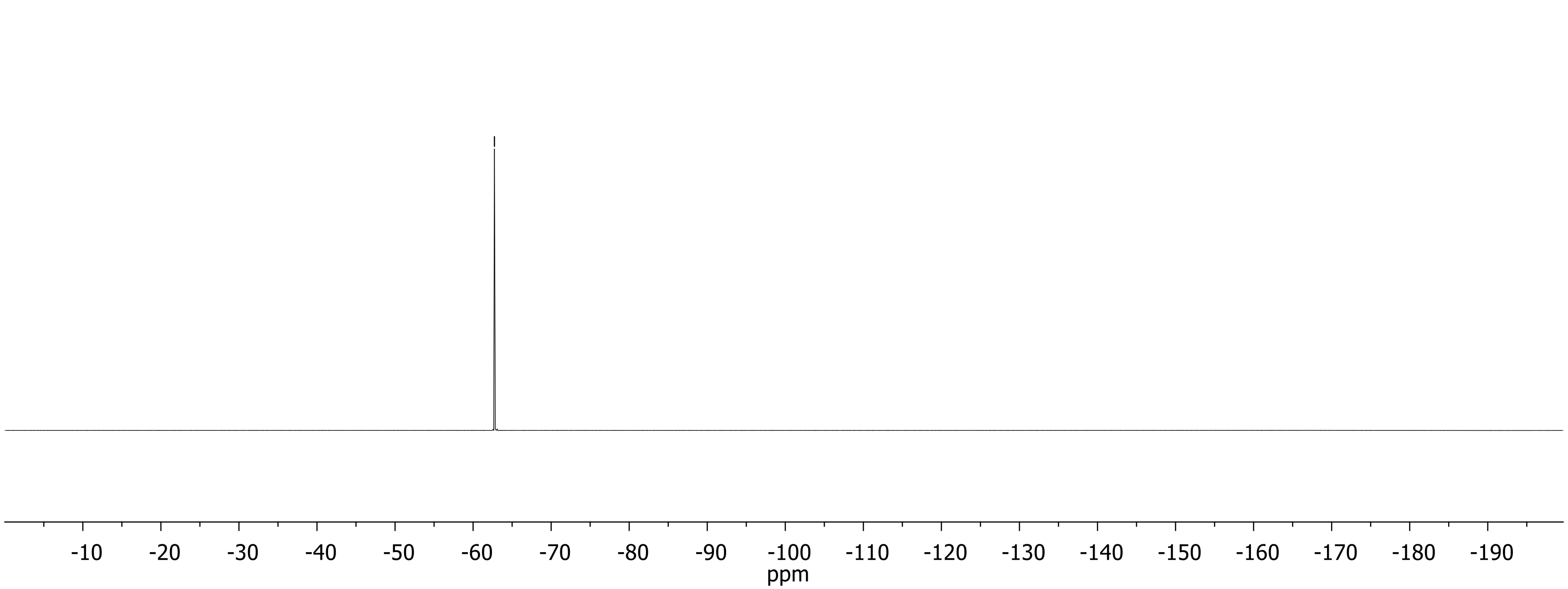

^{1}H NMR in $CDCl_3$ (600 MHz)

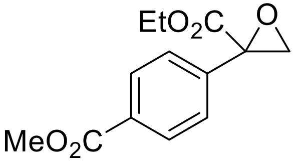

3l

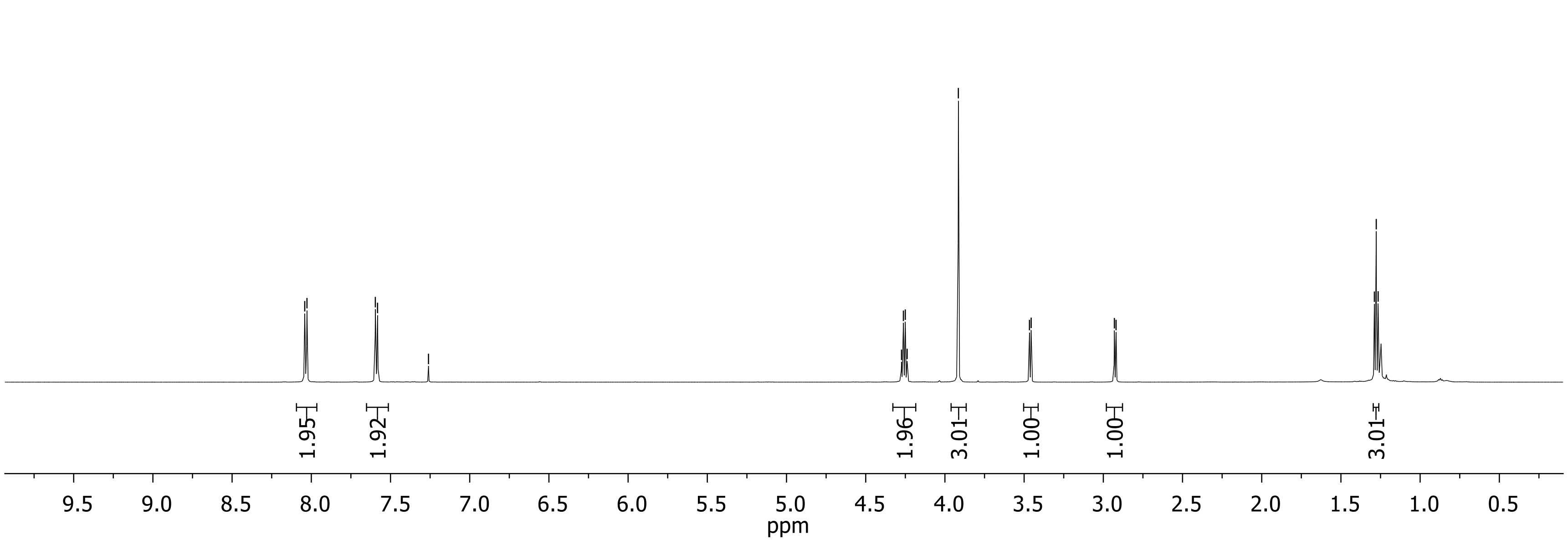

^{13}C {^{1}H} NMR in $CDCl_3$ (151 MHz)

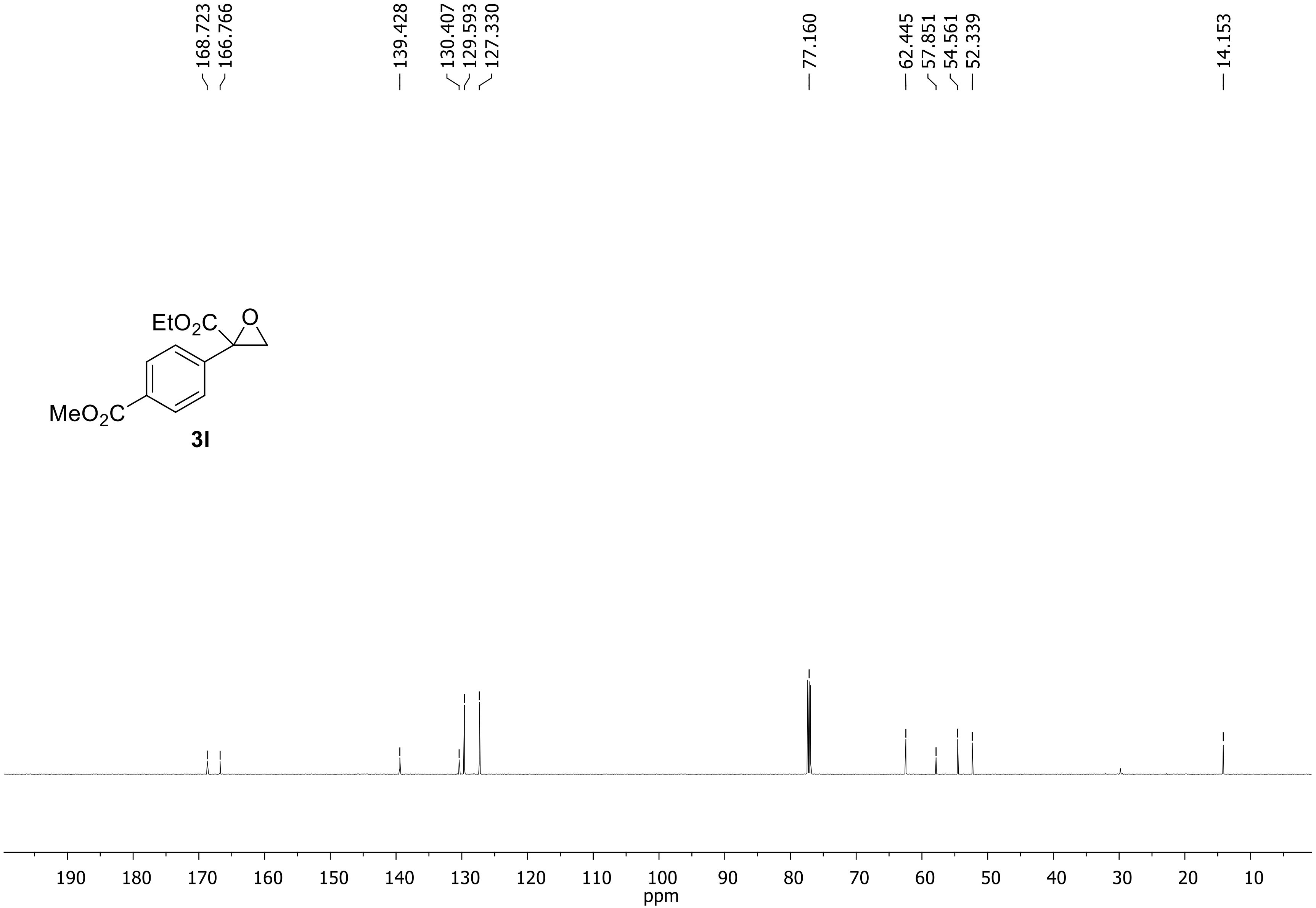

^1H NMR in $CDCl_3$ (600 MHz)

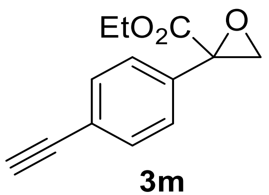

3m

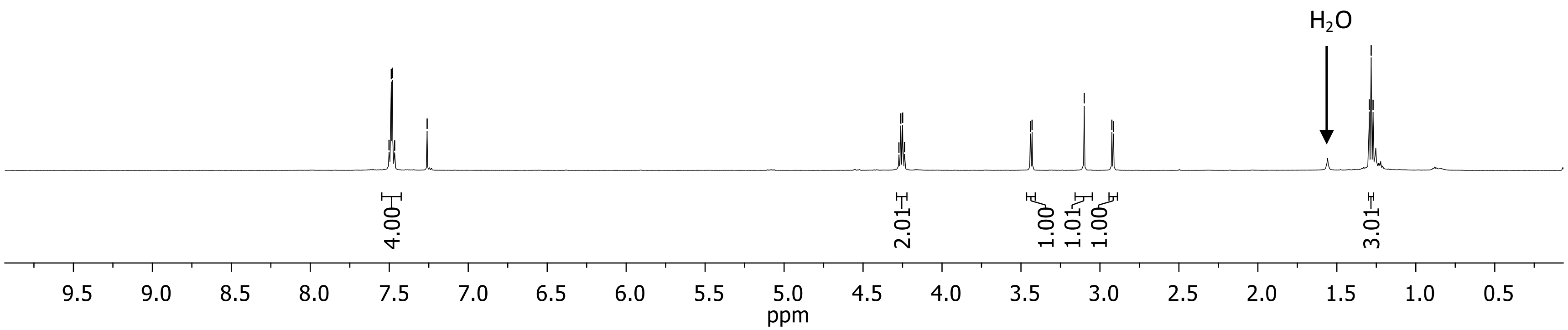

$^{13}C\ \{^1H\}$ NMR in $CDCl_3$ (151 MHz)

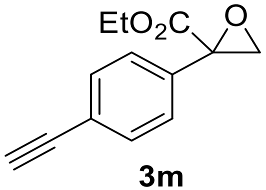

3m

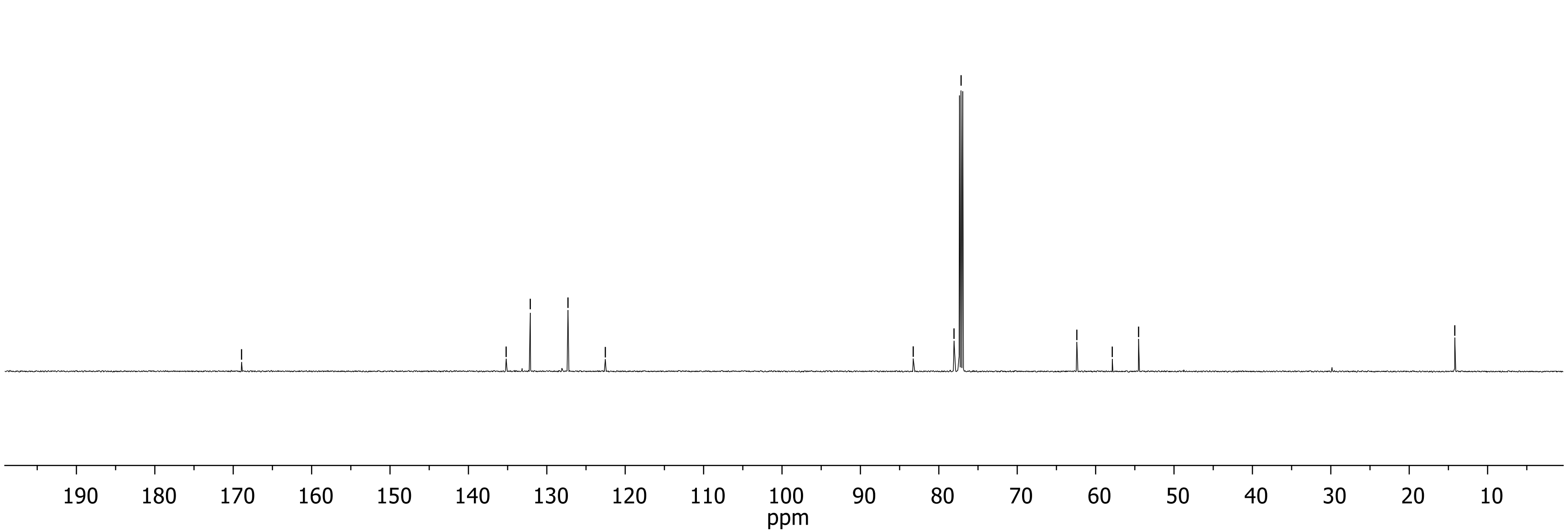

^{1}H NMR in $CDCl_3$ (600 MHz)

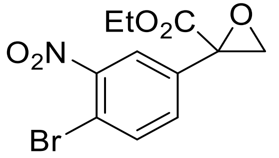

3n

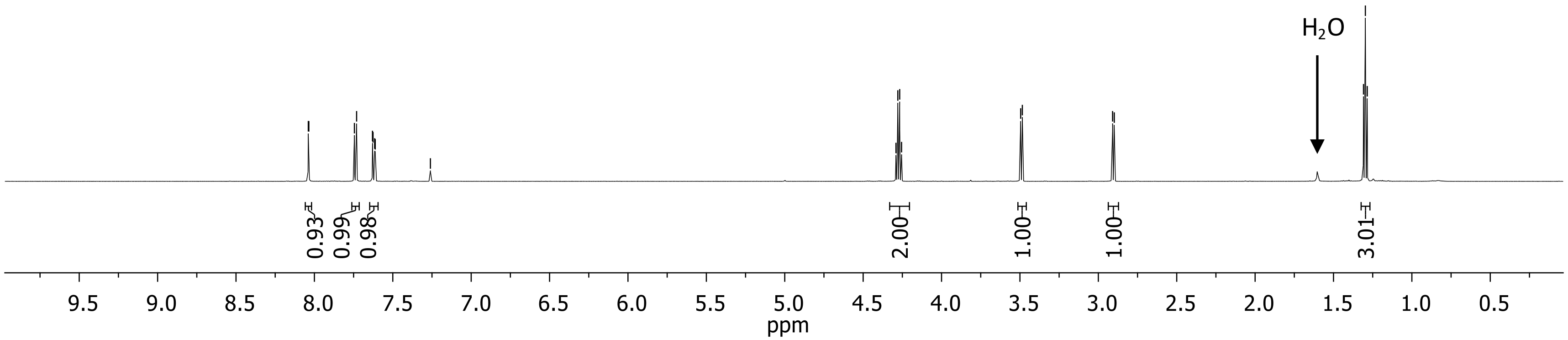

$^{13}C\ \{^{1}H\}$ NMR in $CDCl_3$ (151 MHz)

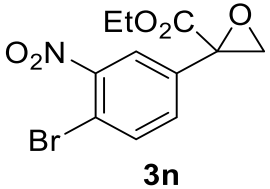

3n

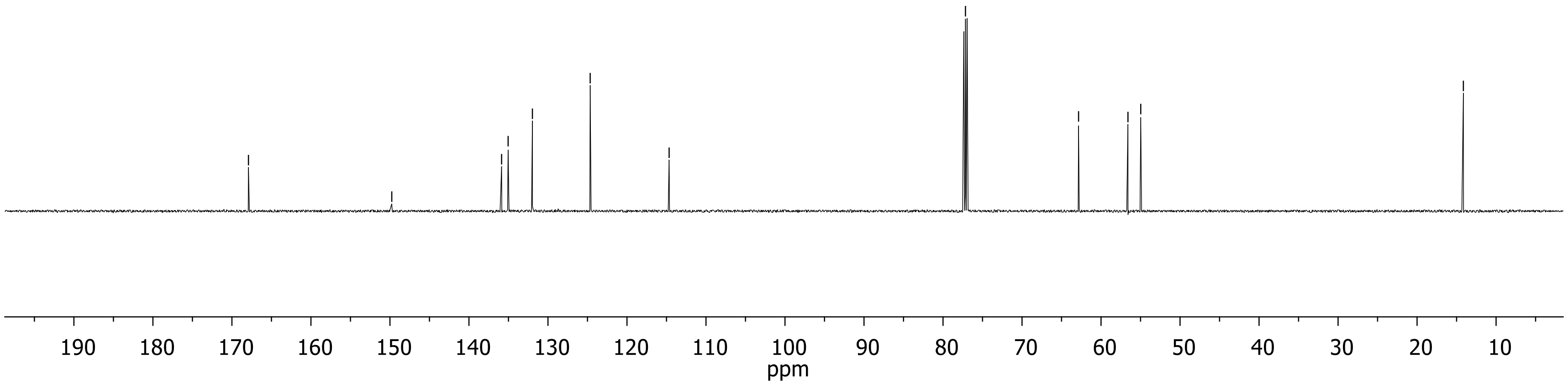

^{1}H NMR in $CDCl_3$ (300 MHz)

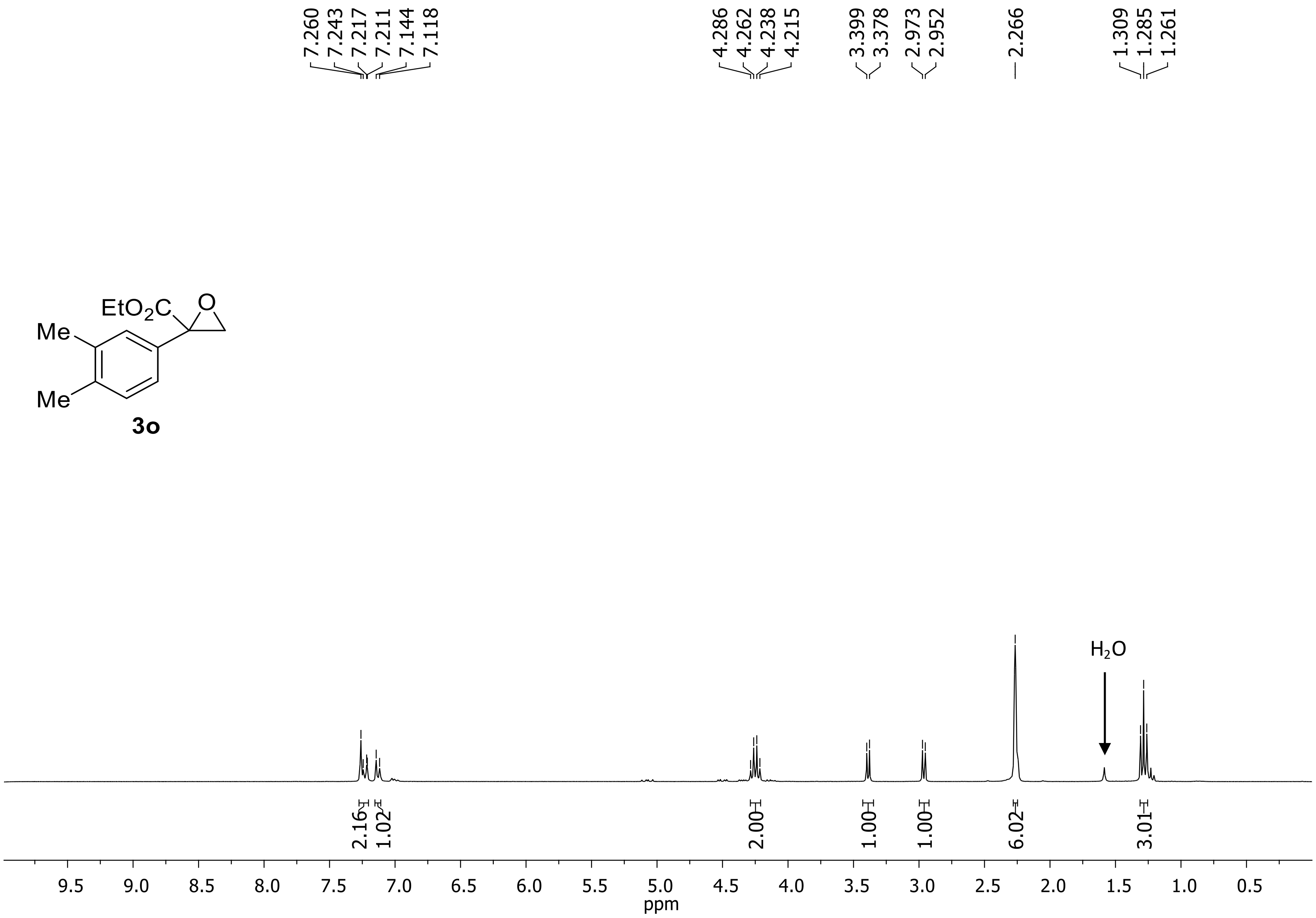

$^{13}C\ \{^{1}H\}$ NMR in $CDCl_3$ (62.5 MHz)

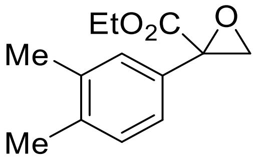

3o

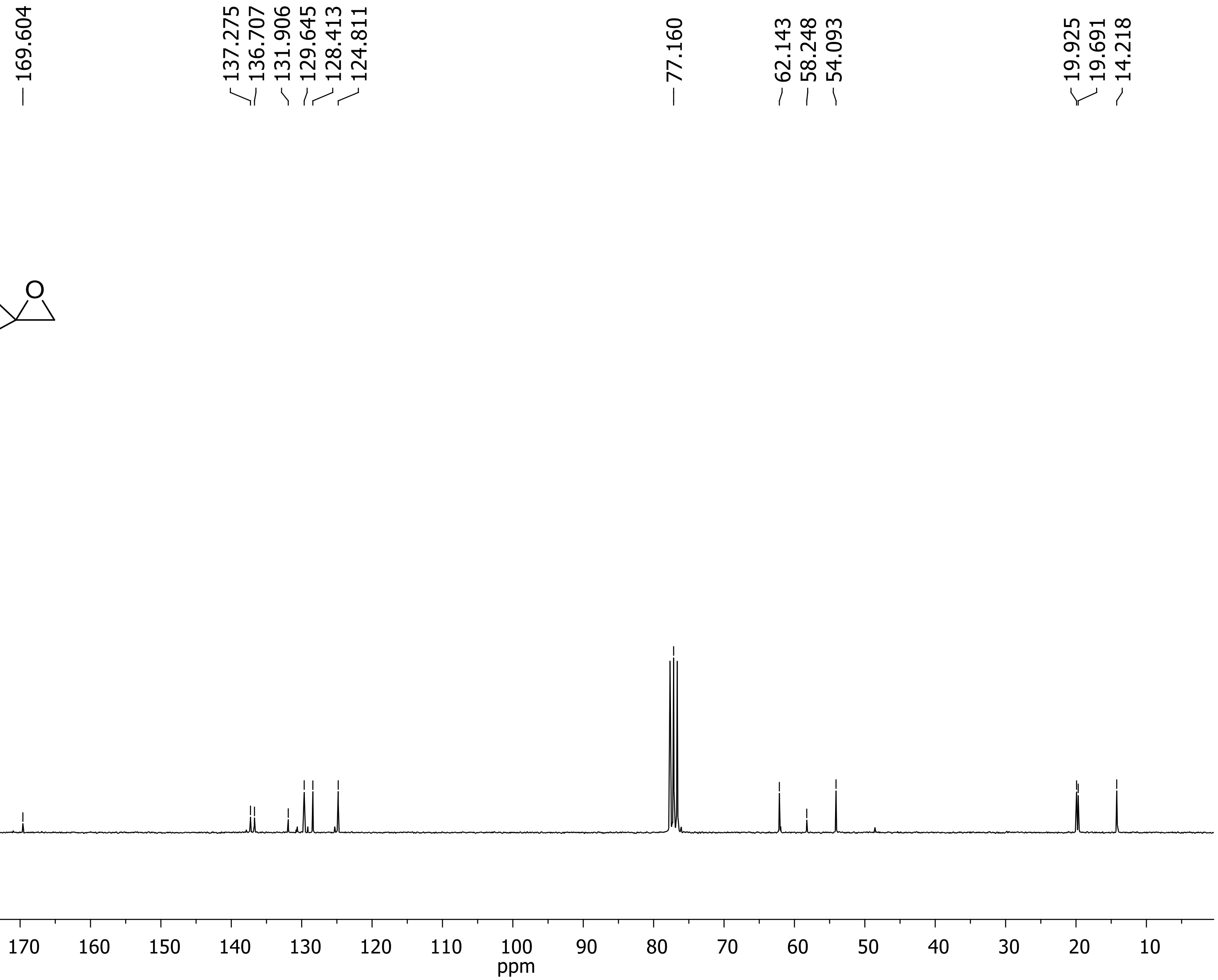

^{1}H NMR in $CDCl_3$ (600 MHz)

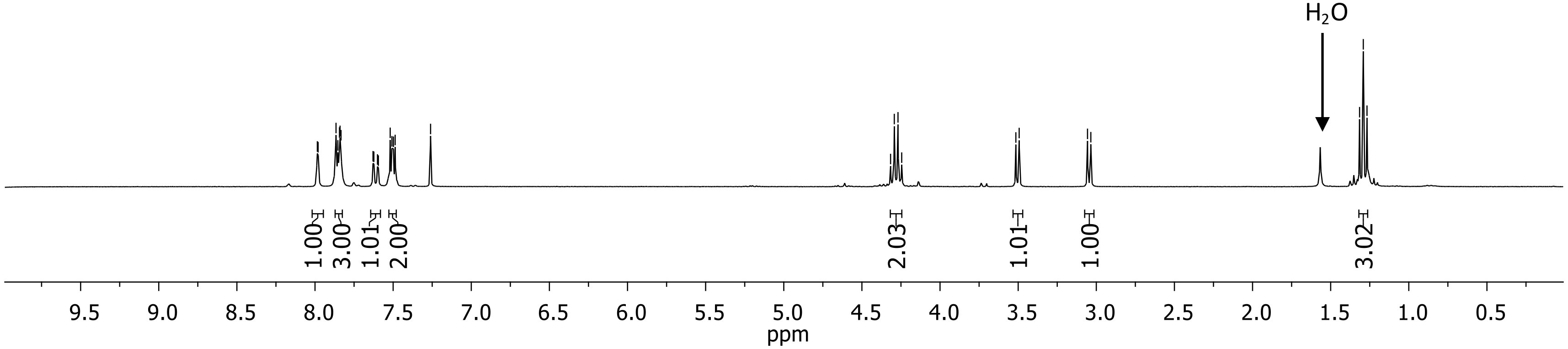

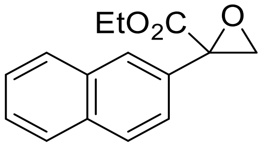

3p

$^{13}C\{^1H\}$ NMR in $CDCl_3$ (151 MHz)

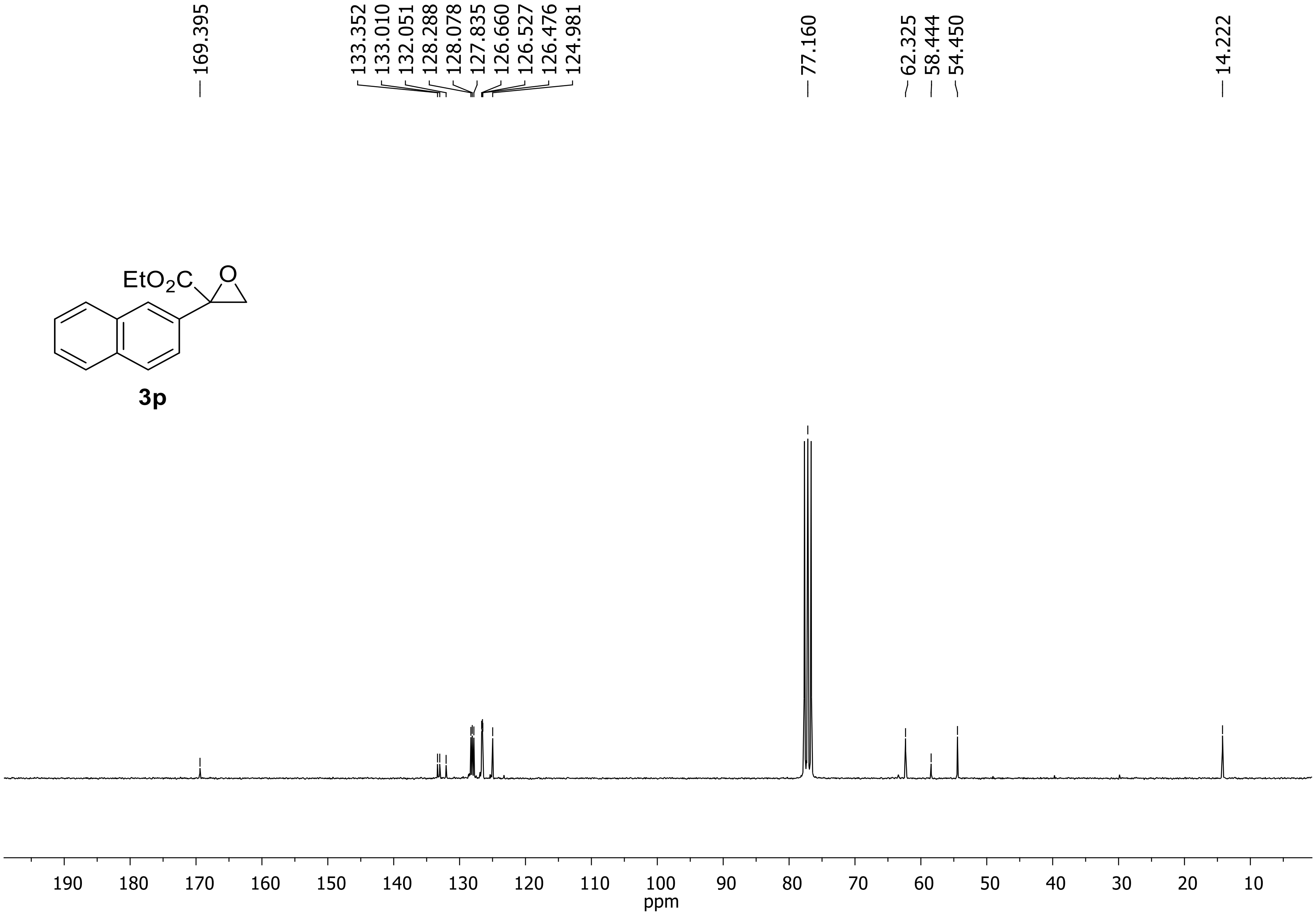

3p

^{1}H NMR in $CDCl_3$ (300 MHz)

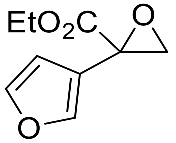

3q

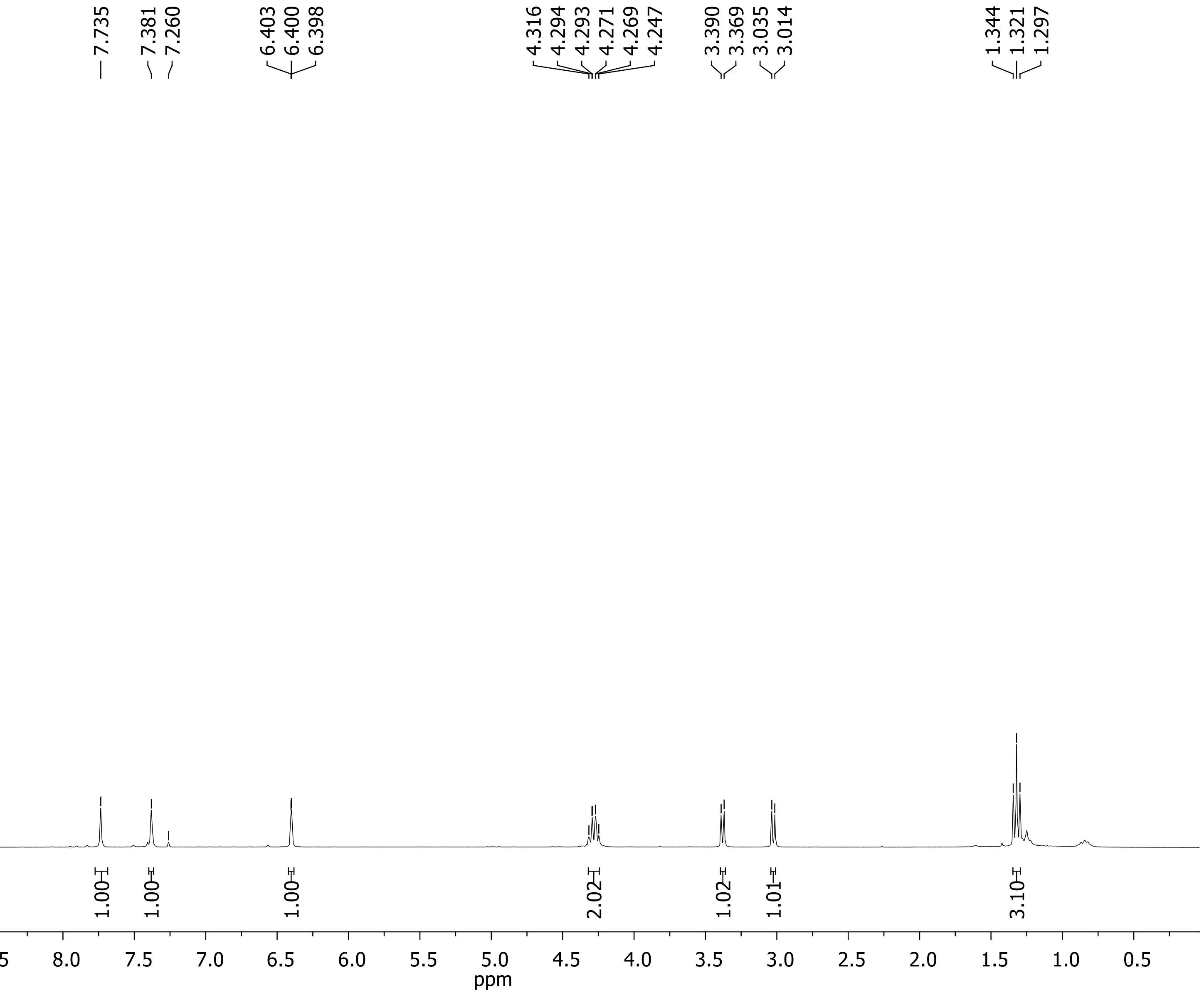

$^{13}C\ \{^{1}H\}$ NMR in $CDCl_3$ (151 MHz)

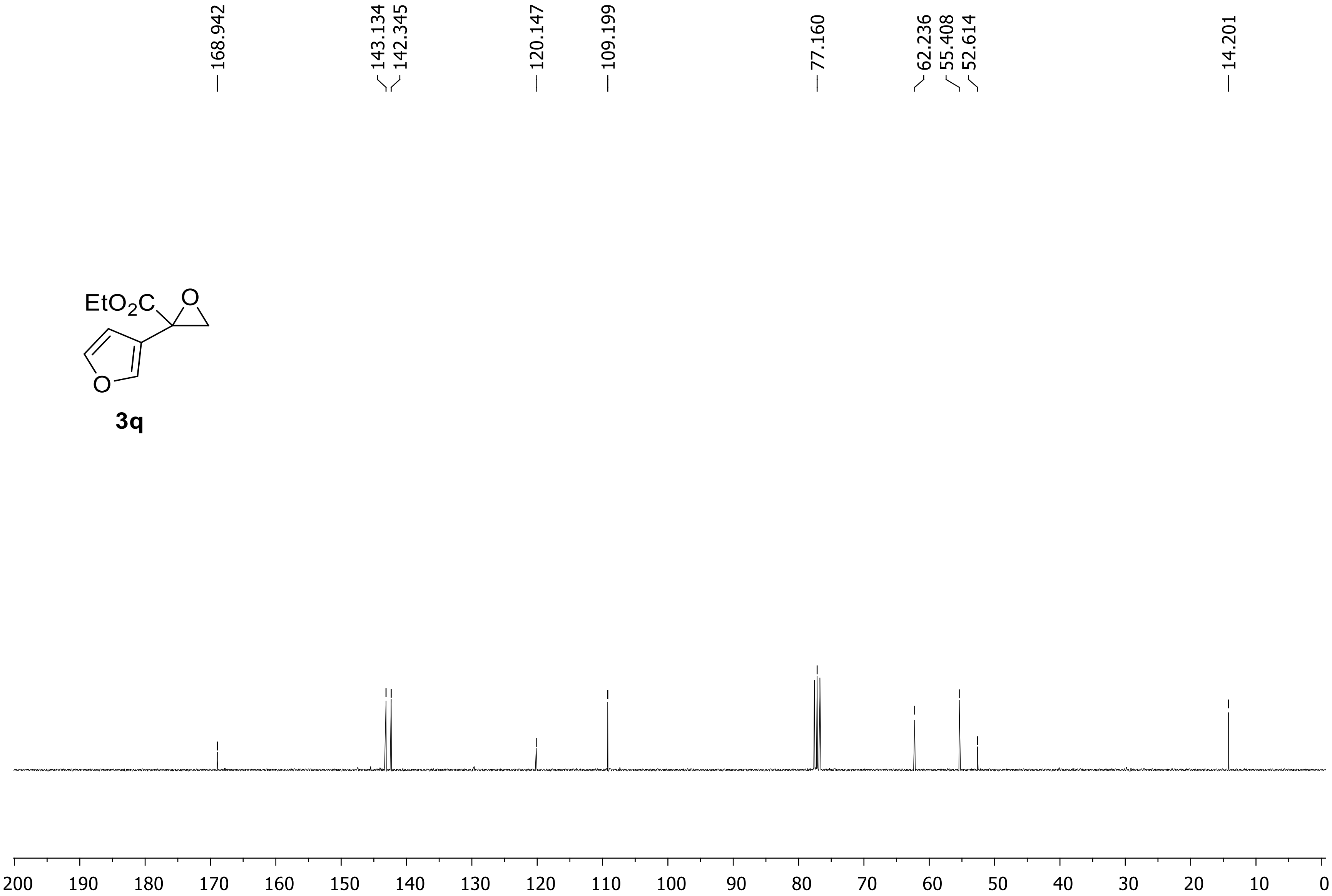

^1H NMR in $CDCl_3$ (300 MHz)

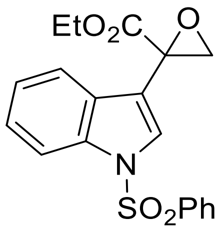

3r

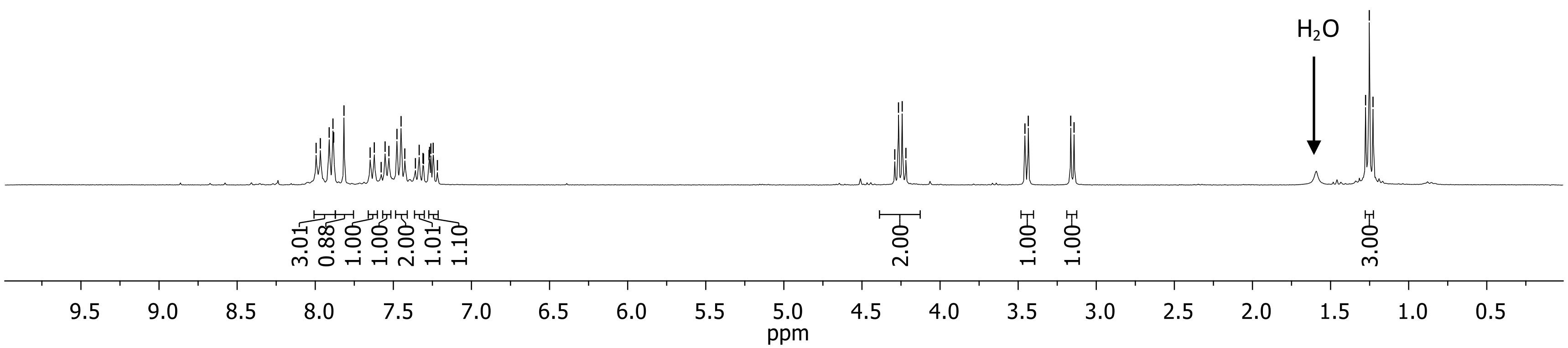

^{13}C {^1H} NMR in $CDCl_3$ (75 MHz)

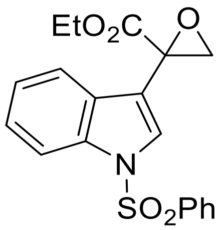

3r

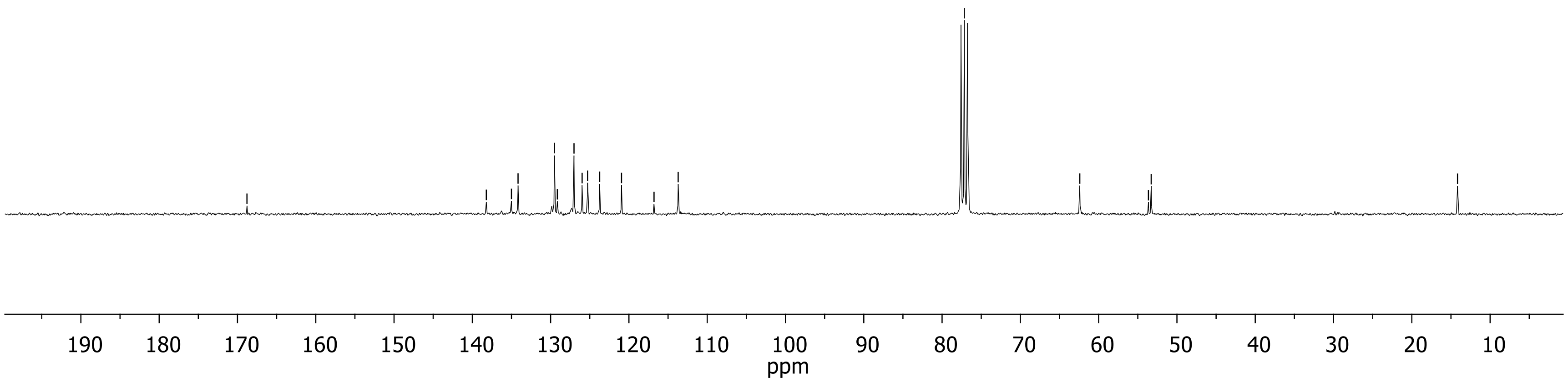

^{1}H NMR in $CDCl_3$ (600 MHz)

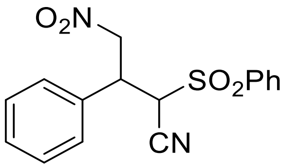

2a

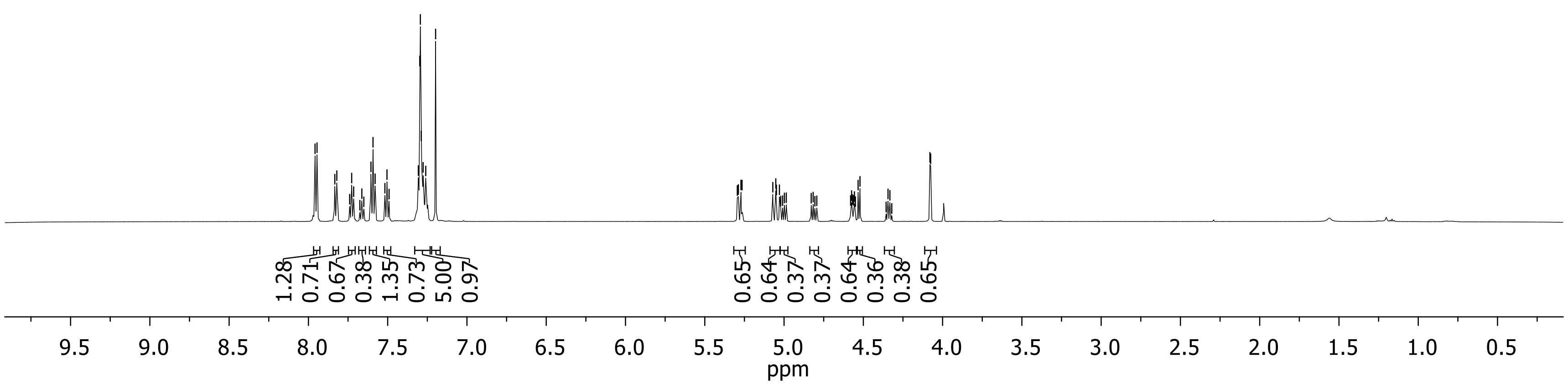

^{13}C {^{1}H} NMR in $CDCl_3$ (151 MHz)

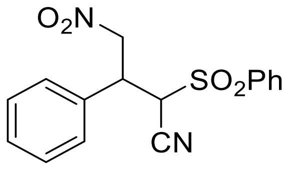

2a

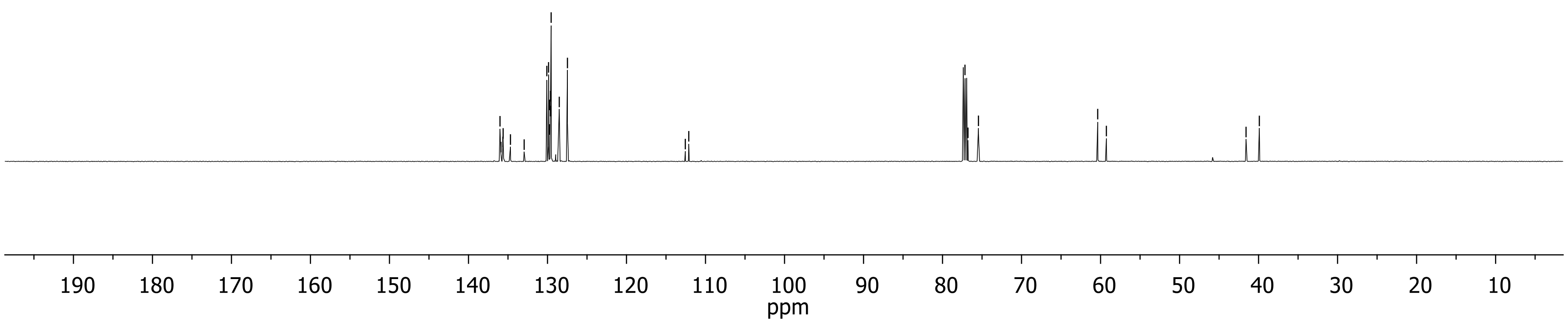

^1H NMR in $CDCl_3$ (300 MHz)

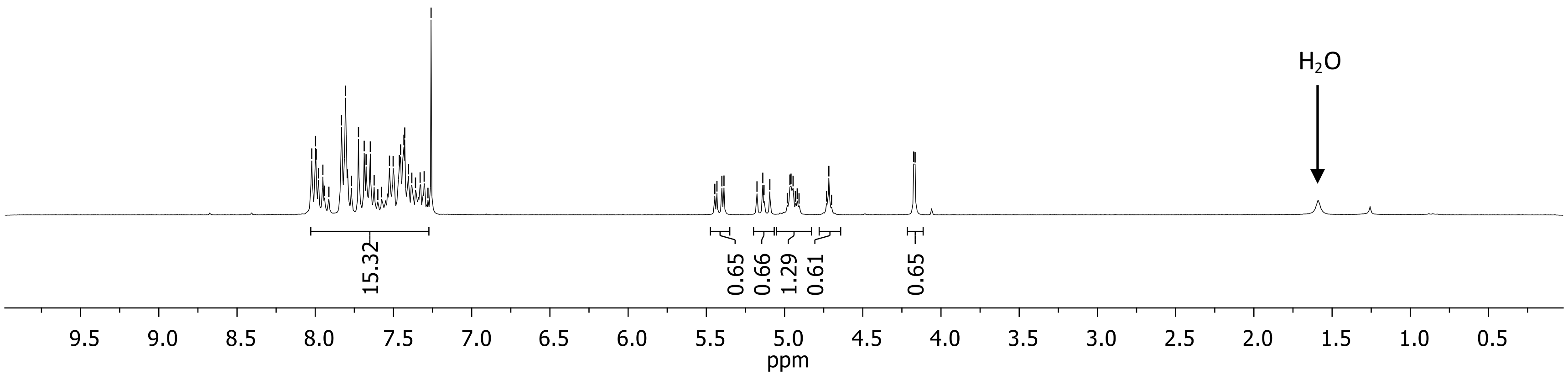

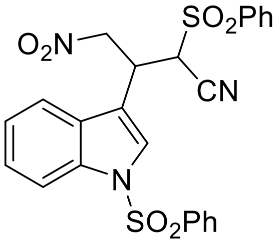

2r

$^{13}C\{^{1}H\}$ NMR in $CDCl_3$ (75 MHz)

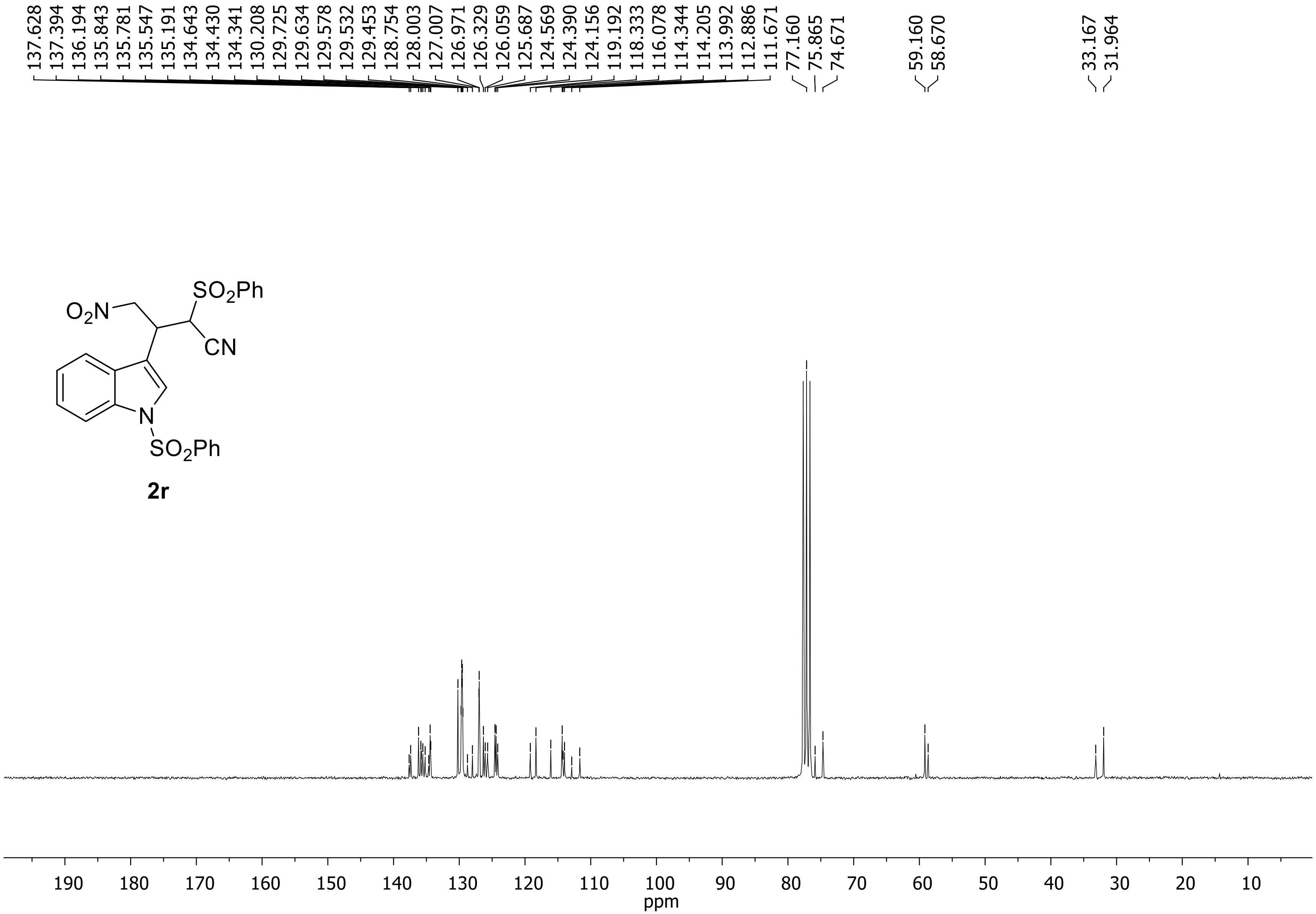

^{1}H NMR in $CDCl_3$ (600 MHz)

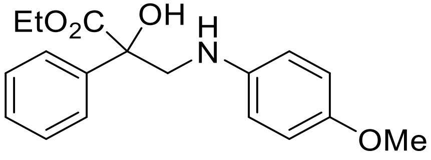

6a

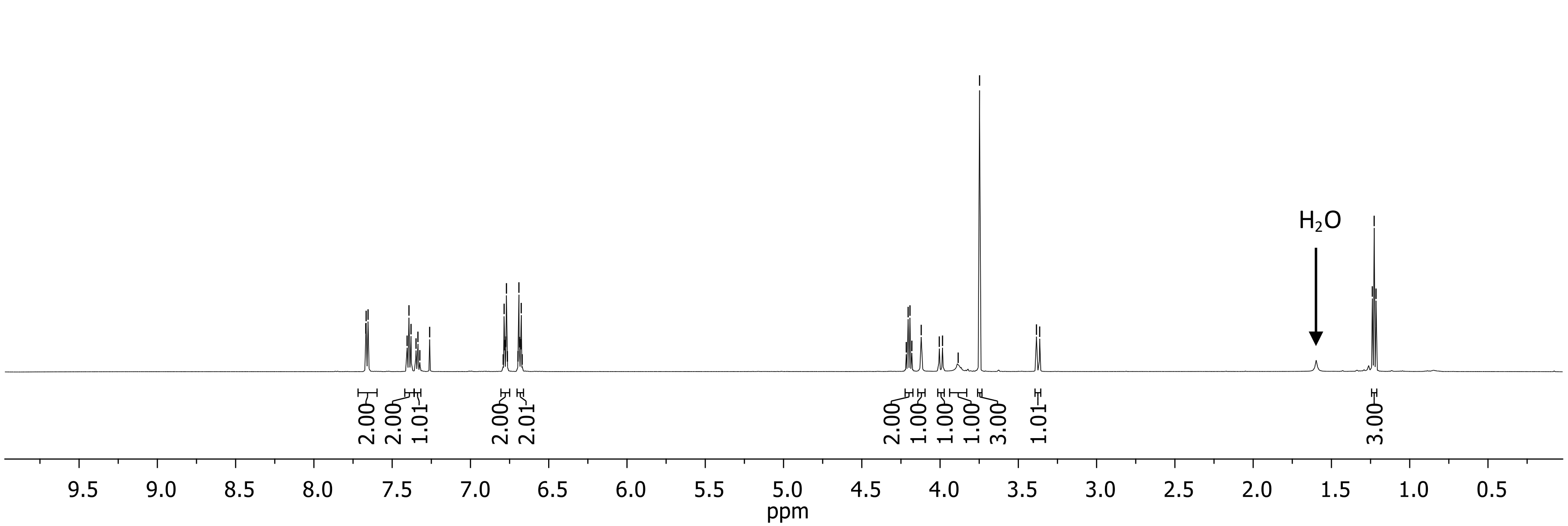

$^{13}C\ \{^1H\}$ NMR in $CDCl_3$ (151 MHz)

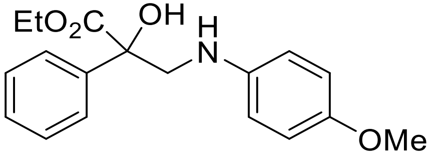

6a

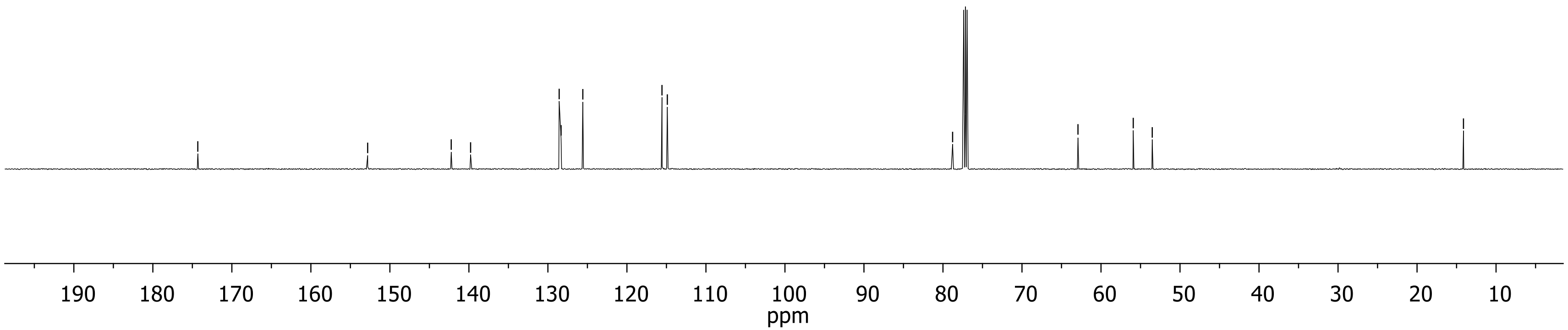

^{1}H NMR in $CDCl_3$ (600 MHz)

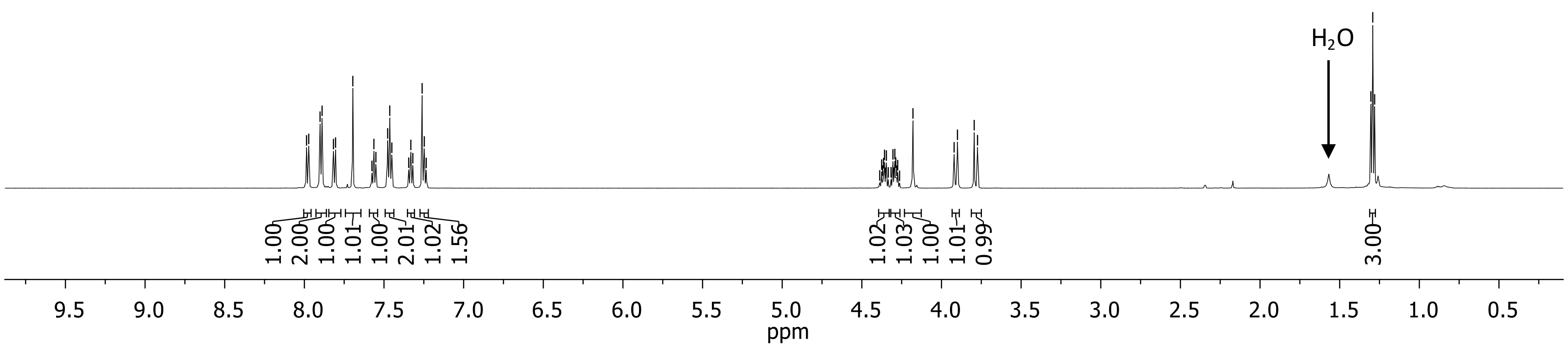

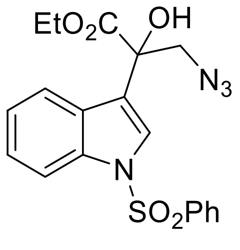

7r

$^{13}C\ \{^{1}H\}$ NMR in $CDCl_3$ (151 MHz)

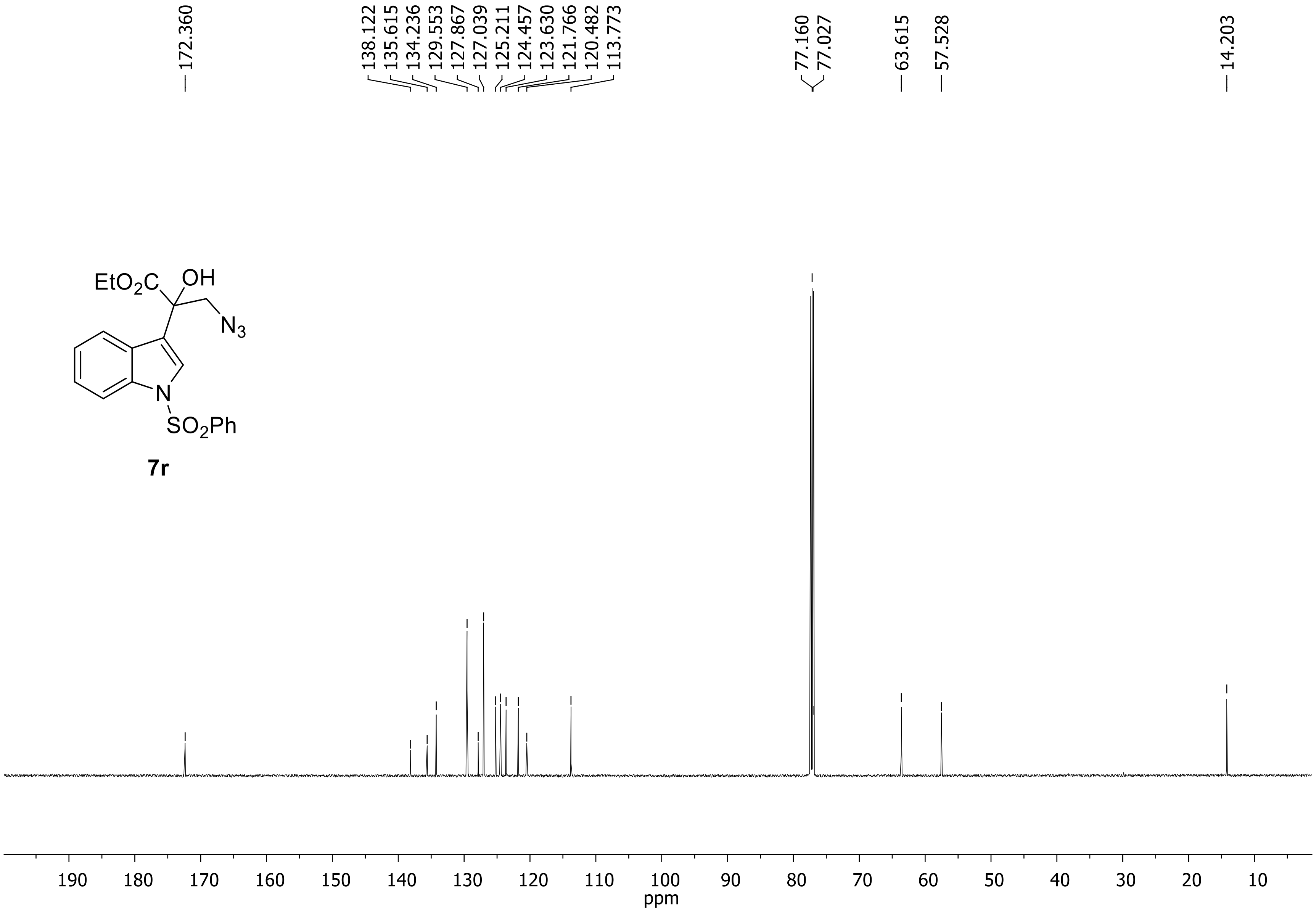

7r

^{1}H NMR in $CDCl_3$ (600 MHz)

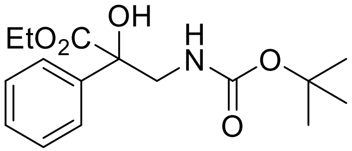

8a

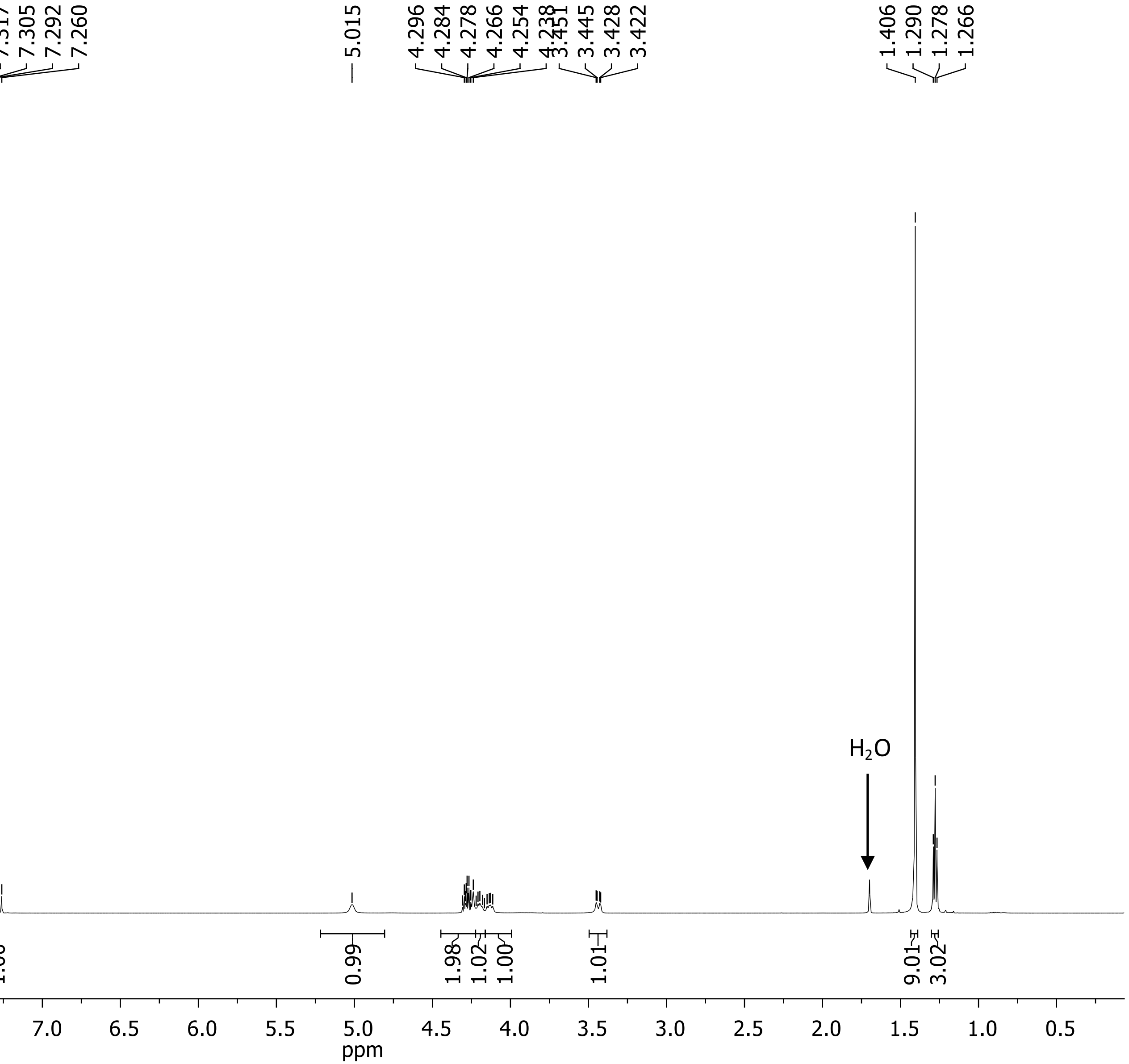

^{1}H NMR in $CDCl_3$ (600 MHz) (Expansion)

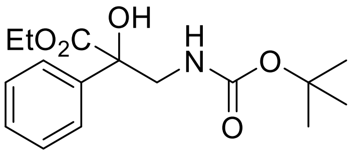

8a

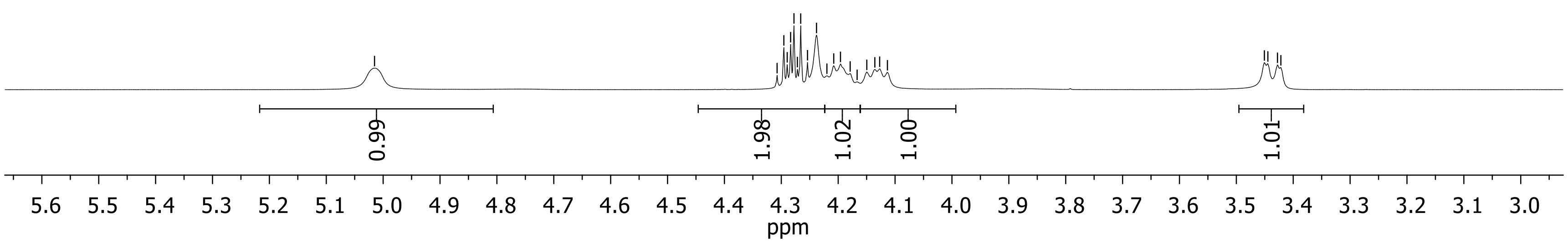

^{13}C {^{1}H} NMR in $CDCl_3$ (151 MHz)

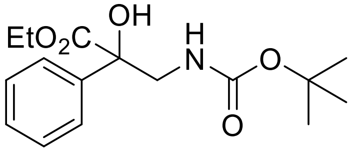

8a

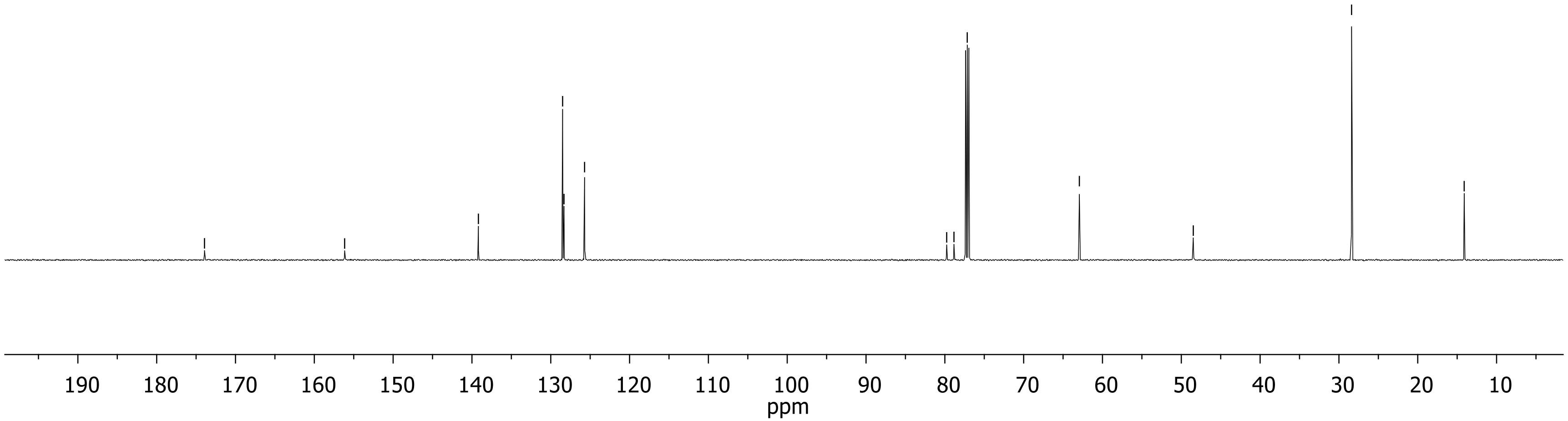

^{1}H NMR in $CDCl_3$ (600 MHz)

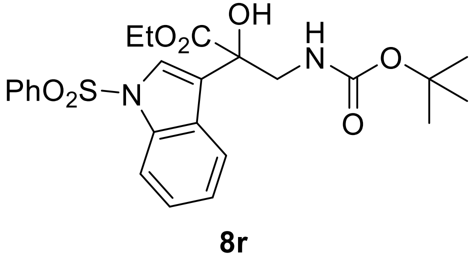

8r

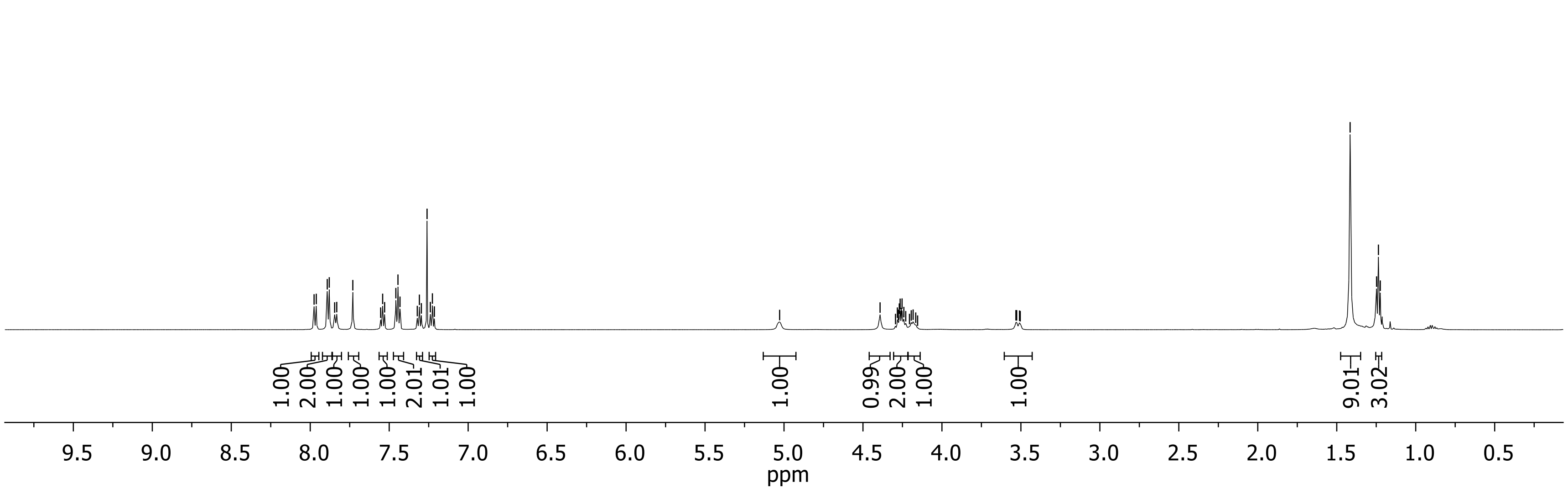

^{1}H NMR in $CDCl_3$ (600 MHz) (Expansion)

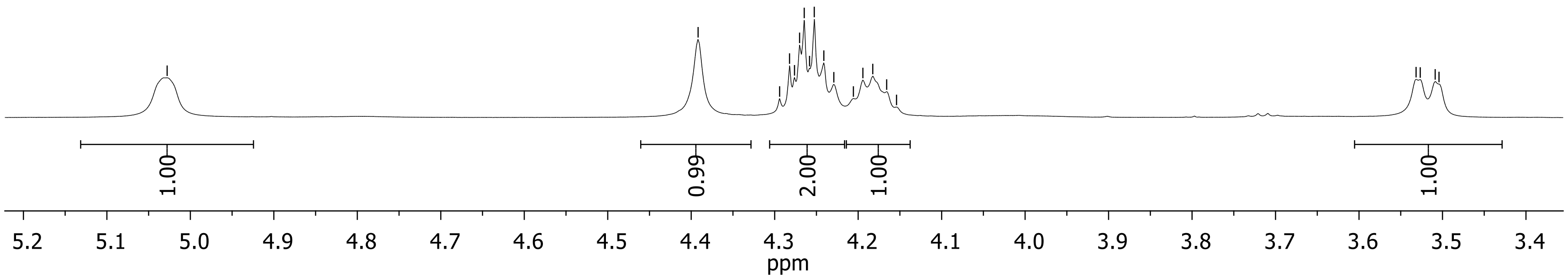

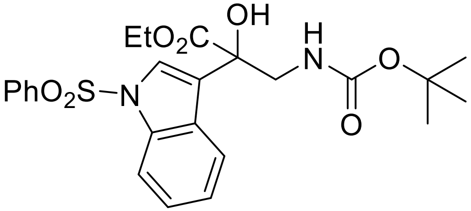

8r

$^{13}C\{^1H\}$ NMR in $CDCl_3$ (151 MHz)

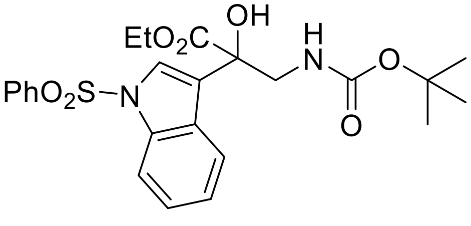

8r

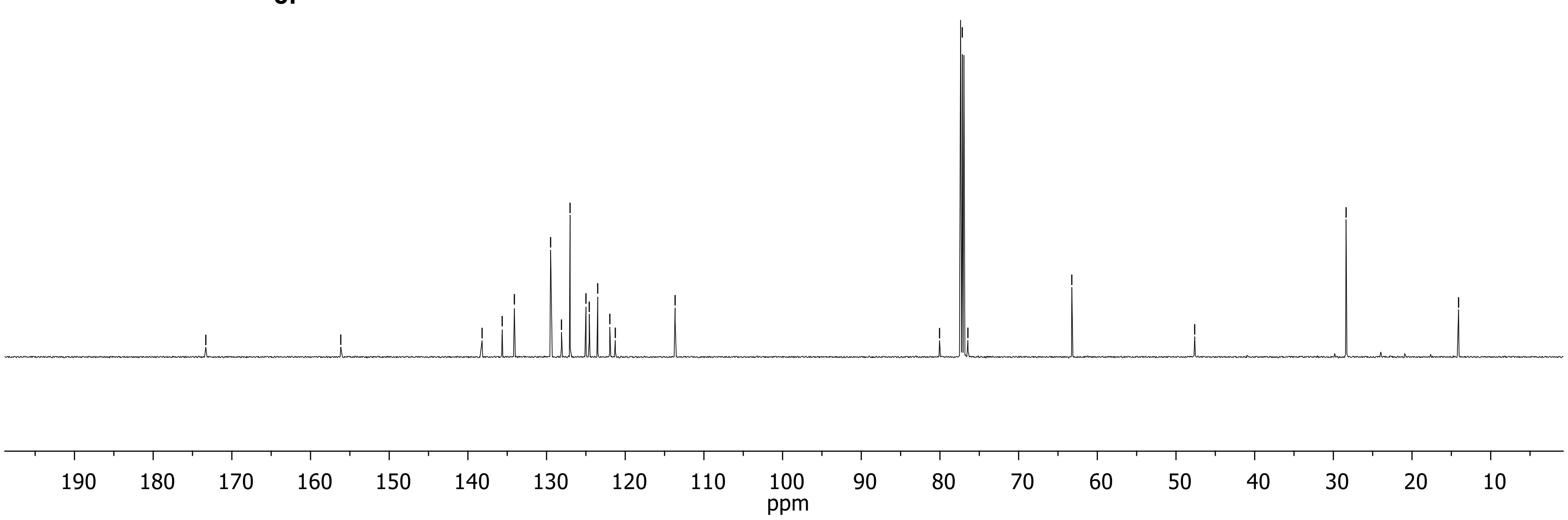

^{1}H NMR in $CDCl_3$ (600 MHz)

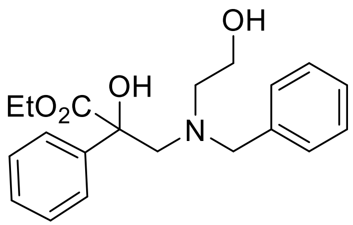

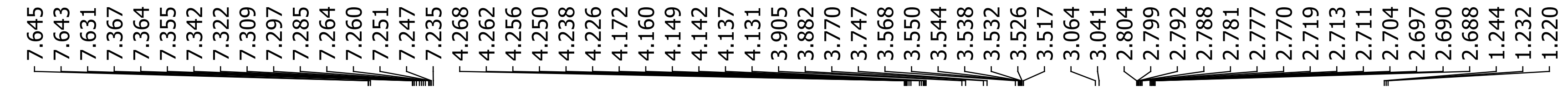

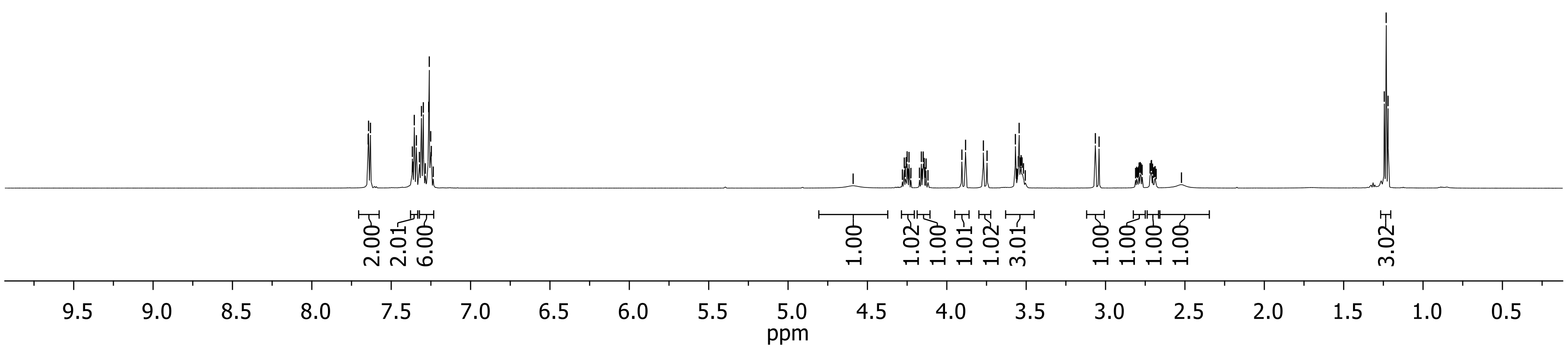

^{1}H NMR in $CDCl_3$ (600 MHz) (Expansion)

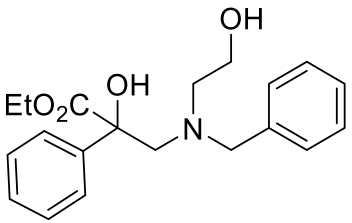

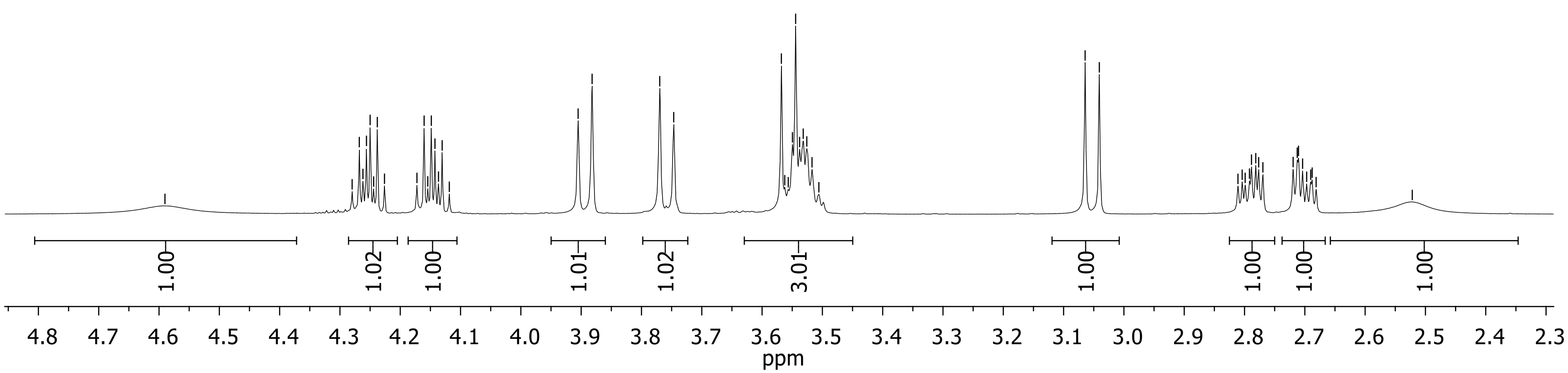

$^{13}C\ \{^{1}H\}$ NMR in $CDCl_3$ (151 MHz)

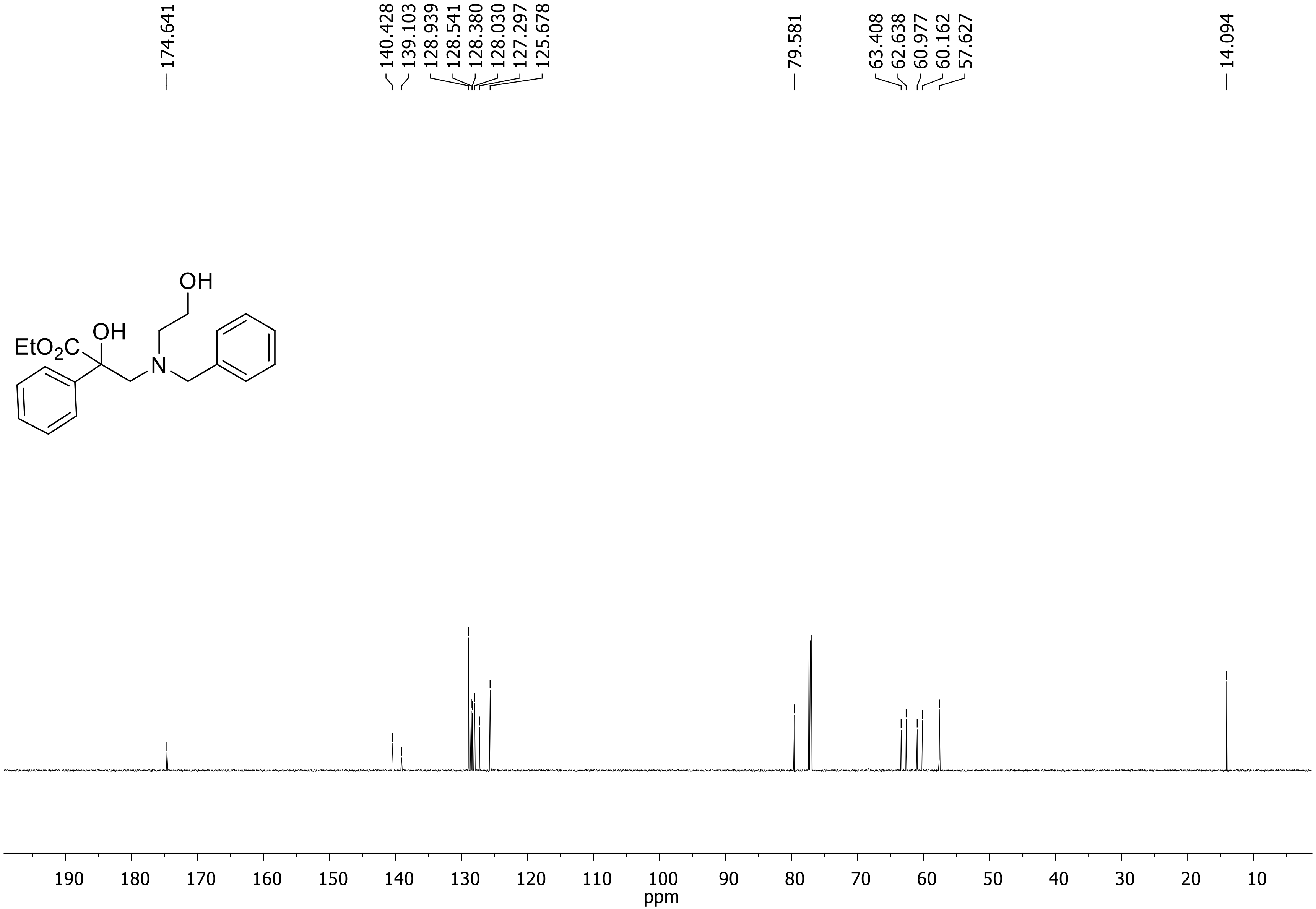

^{1}H NMR in $CDCl_3$ (600 MHz)

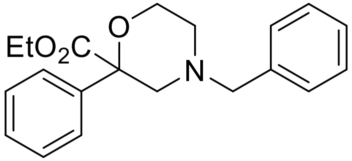

9a

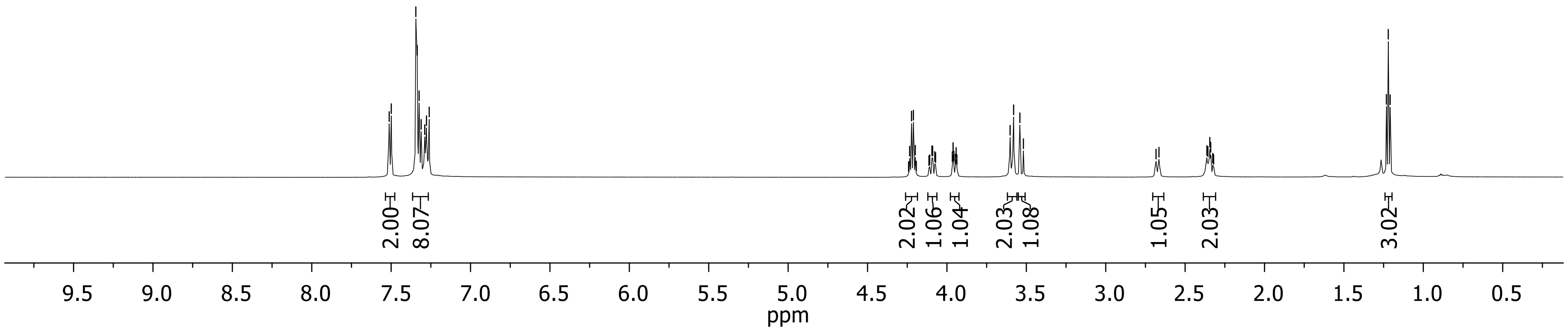

$^{13}C\ \{^1H\}$ NMR in $CDCl_3$ (151 MHz)

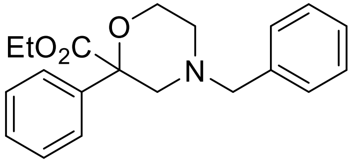

9a

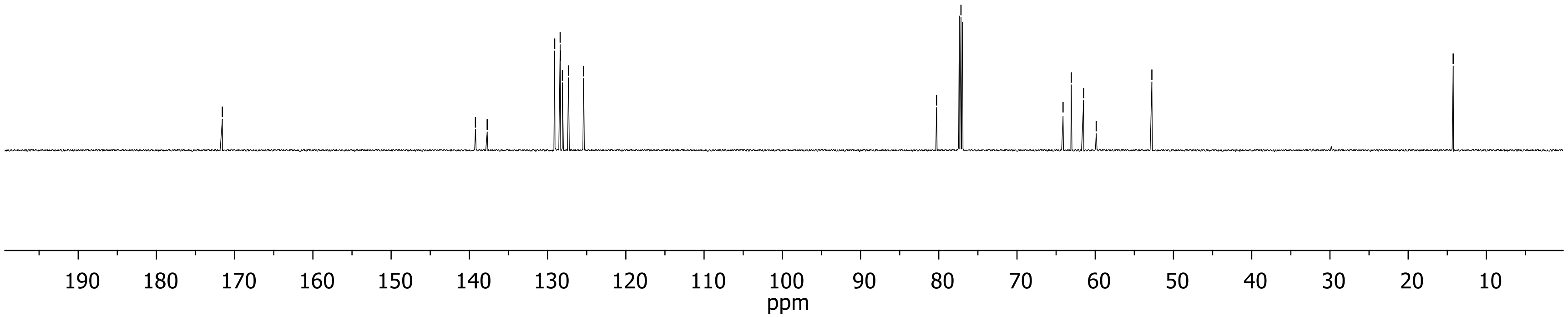

^{1}H NMR in $CDCl_3$ (600 MHz)

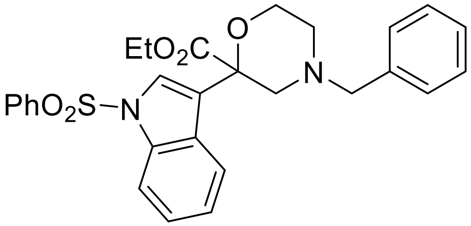

9r

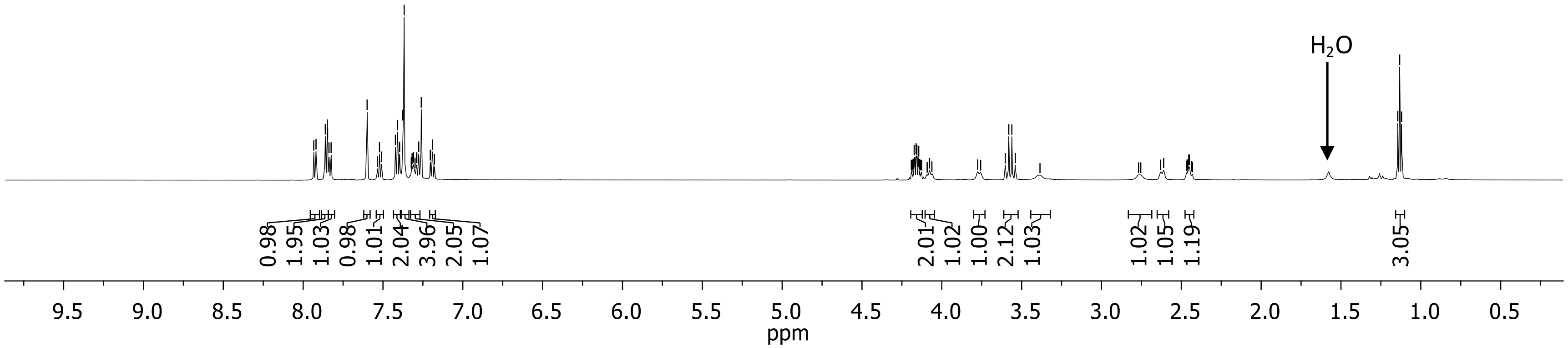

^{1}H NMR in $CDCl_3$ (600 MHz) (Expansion)

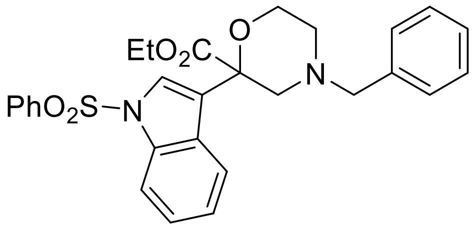

9r

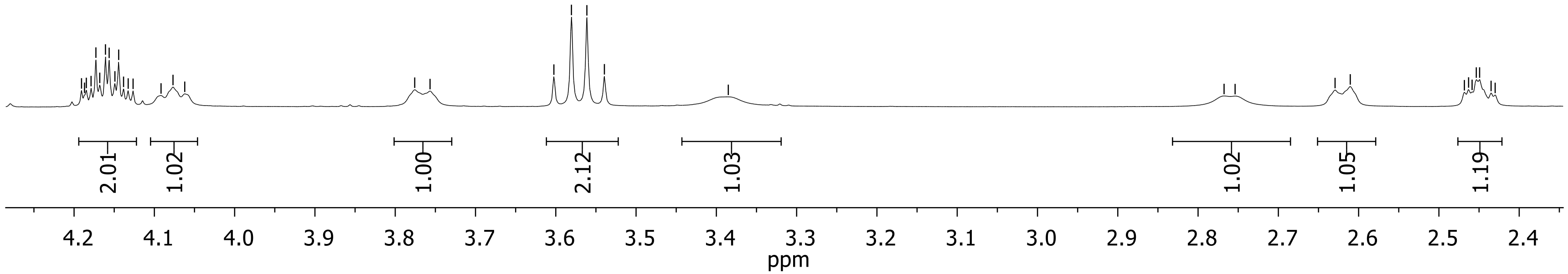

$^{13}C\ \{^{1}H\}$ NMR in $CDCl_3$ (151 MHz)

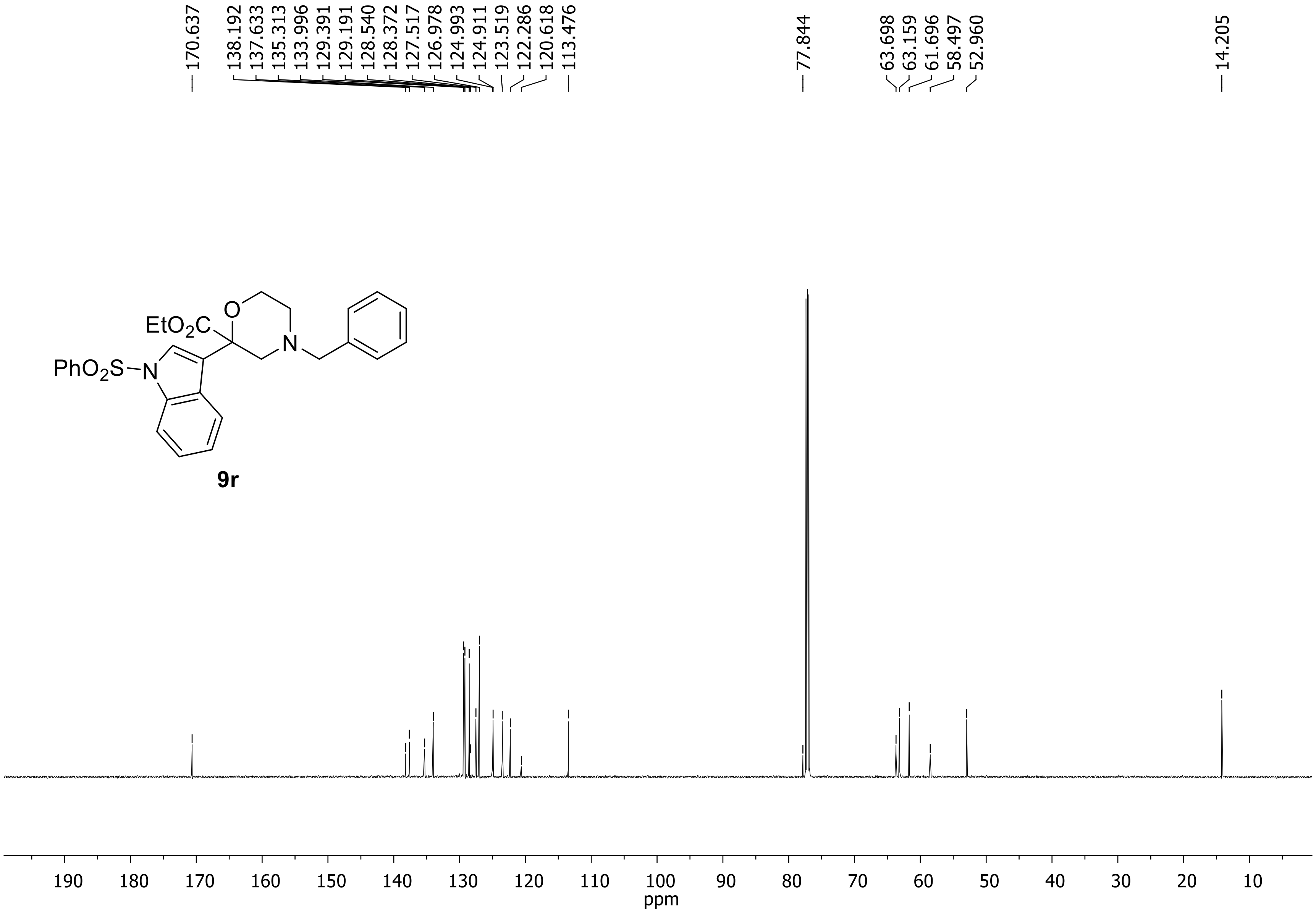

9r

References

1. (a) Volpe, C.; Meninno, S.; Crescenzi, C.; Mancinelli, M.; Mazzanti, A.; Lattanzi, A. *Angew. Chem. Int. Ed.* **2021**, *60*, 23819; (b) Pandit, K. S.; Kupwade, R. V.; Chavan, P. V.; Desai, U. V.; Wadgaonkar, P. P.; Kodam, K. M. *ACS Sust. Chem. Eng.* **2016**, *4*, 3450; (c) Rajkumar, S.; Shankland, K.; Goodman, J. M.; Cobb, A. J. A. *Org. Lett.* **2013**, *15*, 1386; (d) Nemcsok, T.; Rapi, Z.; Bagi, P.; Guan, Y. H.; Orbán, I.; Keglevich, G.; Bakó, P. *Tetrahedron* **2020**, *76*, 130965.

2. (a) Nicely, A. M.; Popov, A. G.; Wendlandt, H. C.; Trammel, G. L.; Kohler, D. G.; Hull, K. L. *Org. Lett.* **2023**, *25*, 5302. (b) Jalil, A. A.; Kurono, N.; Tokuda, M. *Synlett* **2001**, 1944.

3. Li, P.; Zhao, J.; Shi, L.; Wang, J.; Shi, X.; Li, F. *Nat. Commun.* **2018**, *9*, 1972.

4. Li, S.; Xiao, T.; Zhang, X. *Org. Lett.* **2015**, *17*, 3782.

5. Sang, R.; Hu, Y.; Razzaq, R.; Mollaert, G.; Atia, H.; Bentrup, U.; Sharif, M.; Neumann, H.; Junge, H.; Jackstell, R.; Maes, B. U. W., Beller, M. *Nat. Commun.* **2022**, *13*, 4432.

6. Früh, T.; Tombo G. M. R. *Synlett* **1994**, 727.

7. Froestl, W.; Mickel, S. J.; Sprecher, G.; Diel, P. J.; Hall, R. G.; Maier, L.; Strub, D.; Melillo, V.; Baumann, P. A.; Bernasconi, R.; Gentsch, C.; Hauser, K.; Jaekel, J.; Karlsson, G.; Klebs, K.; Maître, L.; Marescaux, C.; Pozza, M. F.; Schmutz, M.; Steinmann, M. W.; Riezen, H.; Vassout, A.; Mondadori, C.; Olpe, H.-R.; Waldmeier, P. C.; Bittiger, H. *J. Med. Chem.* **1995**, *38*, 3313-3331.

8. Attanasi, O. A.; Davoli, P.; Favi, G.; Filippone, P.; Forni, A.; Moscatelli, G.; Prati, F. *Org. Lett.* **2007**, 3461.

9. Shi, W.; Jiang, Z.; He, H.; Xiao, F.; Lin, F.; Sun, Y.; Hou, L.; Shen, L.; Han, L.; Zeng, M.; Lai, K.; Gu, Z.; Chen, X.; Zhao, T.; Guo, L.; Yang, C.; Li, J.; Chen, S. *ACS Med. Chem. Lett.* **2018**, *9*, 94.
